# Supplementary material for: Modeling of African population history using f-statistics is biased when applying all previously proposed SNP ascertainment schemes
Source: PLoS Genet. 2023 Sep 7;19(9):e1010931. doi: 10.1371/journal.pgen.1010931 (PMC10508636; doi:10.1371/journal.pgen.1010931)
Supplement: S1 Fig — 1) residual standard deviation (residual SE) of linear trends and 2) squared Pearson correlation coefficient (R2) for two admixture graph fit metrics (worst f4-statistic residuals, WR, or log-likelihood scores, LL) calculated on unascertained vs. ascertained data. Five thousand best-fitting graphs (according to LL on all sites) of 32,745 possible graphs were selected, and correlation of LL (left-hand panels) or WR (right-hand panels) was explored for graphs fitted on all sites and on ascertained datasets. Results for ascertainment on variants common in Africans (either those having no detectable West Eurasian ancestry or all Africans in the SGDP dataset) are circled in red. As a starting point for generating different ascertained datasets, we used 11,706,773 sites (with no missing data at the group level) polymorphic in a set of 48 archaic and African groups composed of 97 individuals (S1 Table). Thirty-eight site subsampling schemes were explored (see a list in the legend for Fig 2). The size of the resulting SNP panels is coded by point size, and ten broad ascertainment types are coded by color according to the legend in the upper right corner. The 97.5th (in the case of residual SE) or 2.5th (in the case of R2) LL or WR percentiles of all the thinned replicates combined, including those on all sites and AT/GC sites, are marked by brown lines. Areas of the plots where ascertainments are considered biased according to these thresholds are highlighted in red on the left-hand side of the plots. Scatterplots illustrating effects of selected ascertainment schemes (marked with numbers 1 to 7) on LL or WR are shown in the middle of the figure. Each dot on these scatterplots corresponds to a distinct admixture graph topology. (PDF) [file pgen.1010931.s001.pdf]

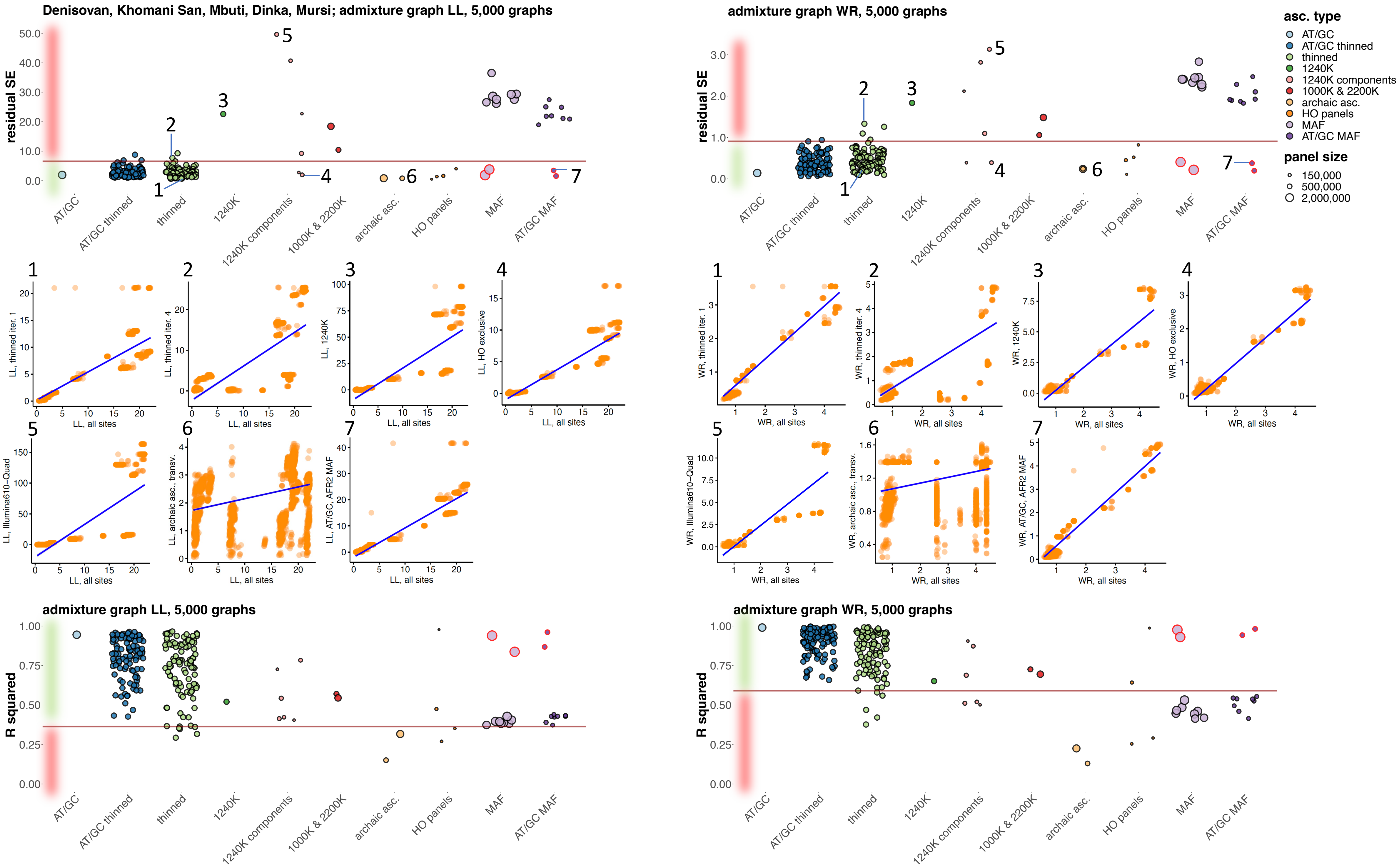

Denisovan, Khomani San, Mbuti, Dinka, Mursi; admixture graph LL, 5,000 graphs
admixture graph WR, 5,000 graphs
residual SE
R squared
asc. type
AT/GC
AT/GC thinned
thinned
1240K
1240K components
1000K & 2200K
archaic asc.
HO panels
MAF
AT/GC MAF
panel size
150,000
500,000
2,000,000
LL, all sites
LL, thinned iter. 1
LL, thinned iter. 4
LL, 1240K
LL, HO exclusive
LL, Illumina610−Quad
LL, archaic asc., transv.
LL, AT/GC, AFR2 MAF
WR, all sites
WR, thinned iter. 1
WR, thinned iter. 4
WR, 1240K
WR, HO exclusive
WR, Illumina610−Quad
WR, archaic asc., transv.
WR, AT/GC, AFR2 MAF
admixture graph LL, 5,000 graphs
admixture graph WR, 5,000 graphs
