# Supplementary figures and images for: Modeling of African population history using f-statistics is biased when applying all previously proposed SNP ascertainment schemes
Source: PLoS Genet. 2023 Sep 7;19(9):e1010931. doi: 10.1371/journal.pgen.1010931 (PMC10508636; doi:10.1371/journal.pgen.1010931)

a

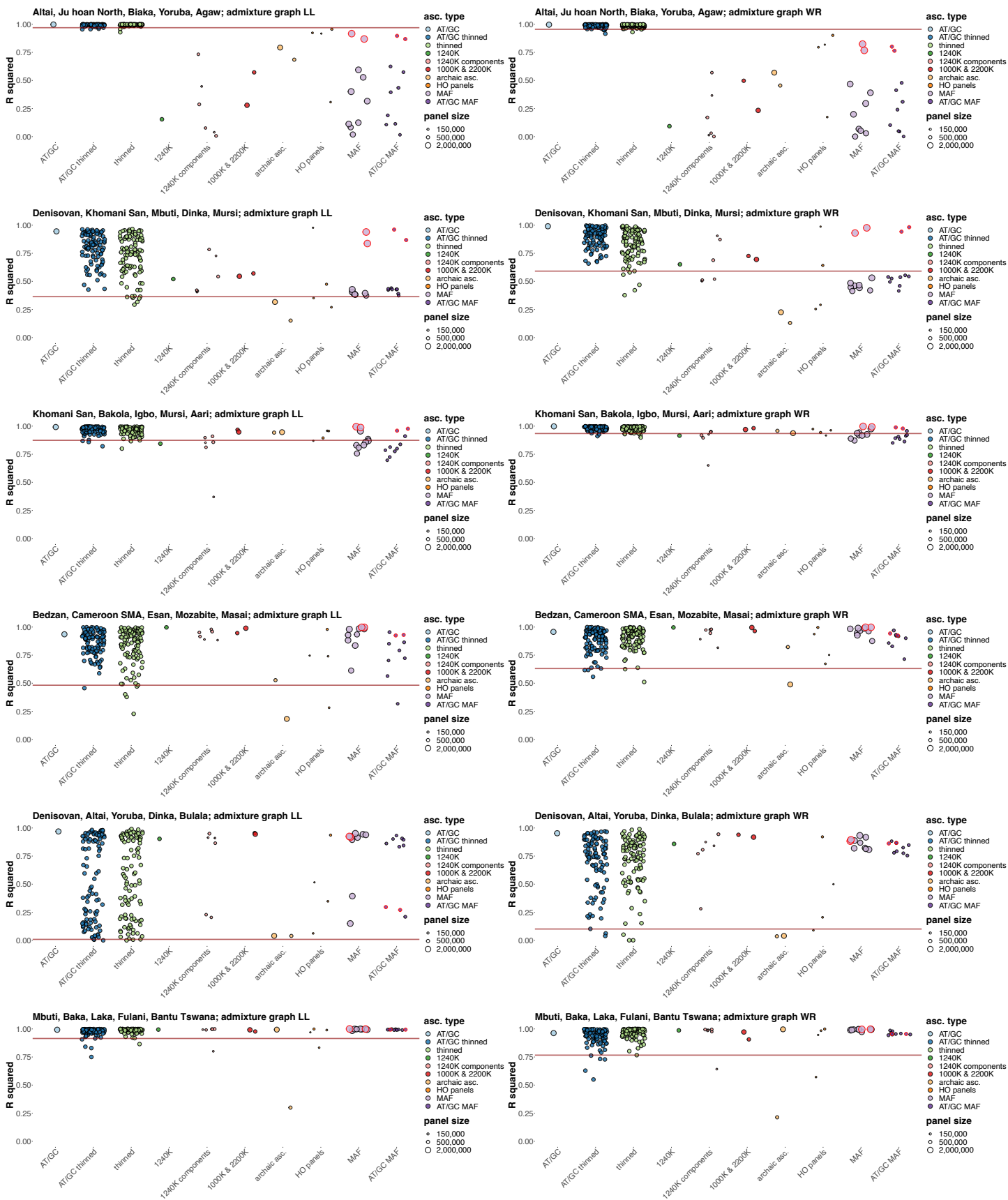

b

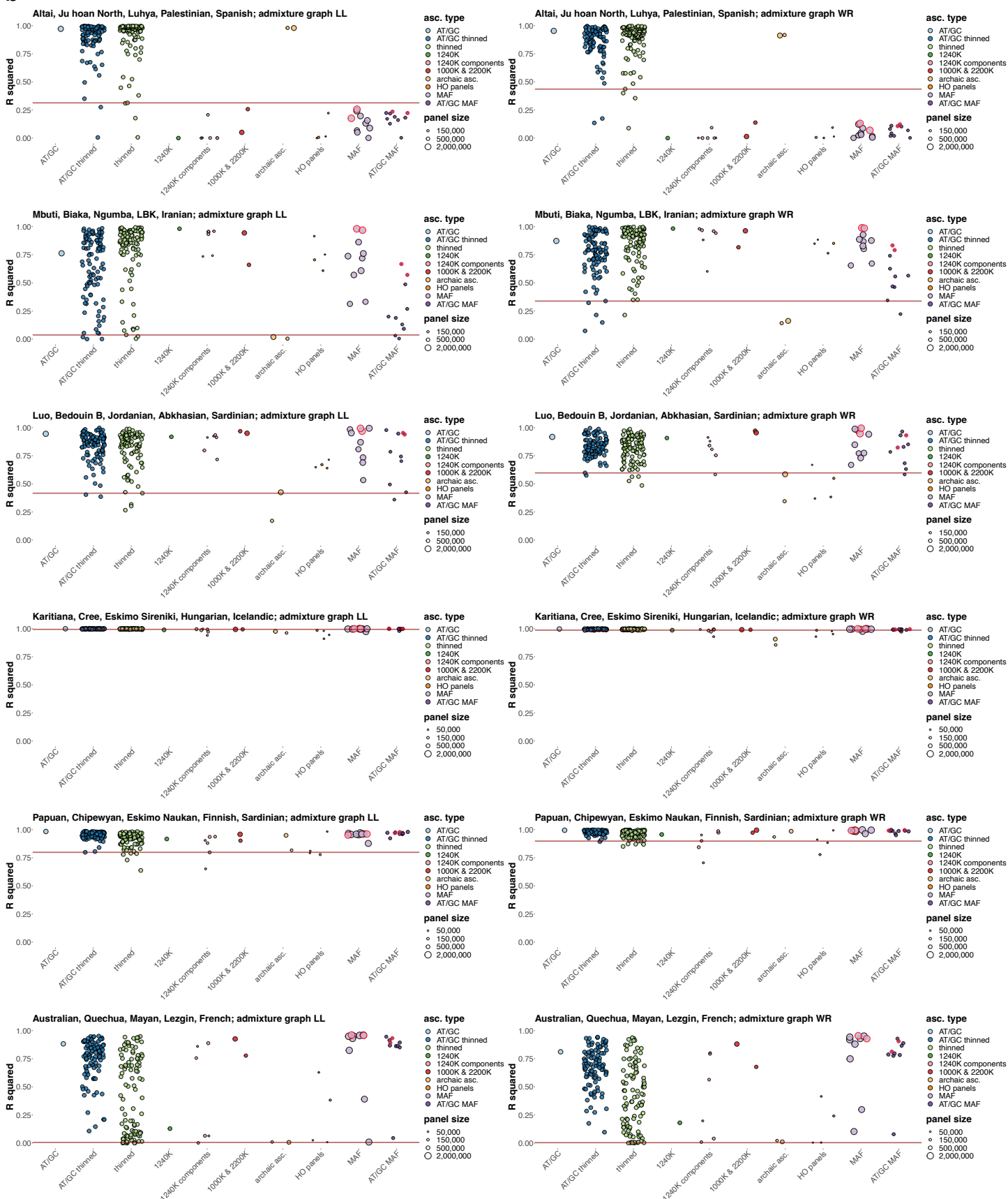

Supplement: S2 Fig — Five thousand best-fitting graphs (according to log-likelihood scores on all sites) of 32,745 graphs were selected for each combination of populations, and correlation of admixture graph log-likelihood scores (LL) or worst f4-statistic residuals (WR) was explored for graphs fitted to unascertained vs. ascertained datasets. Results are shown for twelve population combinations indicated in plot titles (panels a, b). Results for ascertainment on variants common in Africans (either those having no detectable West Eurasian ancestry or on all Africans in the SGDP dataset) are circled in red. As a starting point for generating different ascertainments, we used either 11,706,773 sites (with no missing data at the group level) polymorphic in a set of 48 archaic and African groups composed of 97 individuals in total, or 10,051,585 such sites in 59 archaic, African, European, and Middle Eastern groups composed of 120 individuals, or 5,296,653 such sites in 51 Papuan, Native American/Siberian, European, Anatolian, and Caucasian groups composed of 112 individuals (S1 Table). Thirty eight site subsampling schemes were explored (see a list in the legend for Fig 2). The size of the resulting SNP panels is coded by point size, and ten broad ascertainment types are coded by color according to the legends. R2 values for LL are plotted in the left-hand panels, and R2 values for WR are plotted in the right-hand panels. The 2.5th LL or WR percentiles of all the thinned replicates combined, including those on all sites and AT/GC sites, are marked by brown lines. (PDF) [file pgen.1010931.s002.pdf]

a

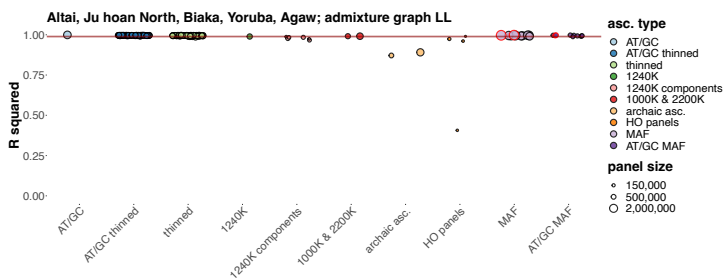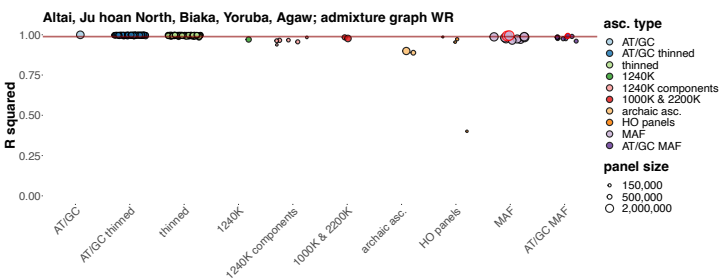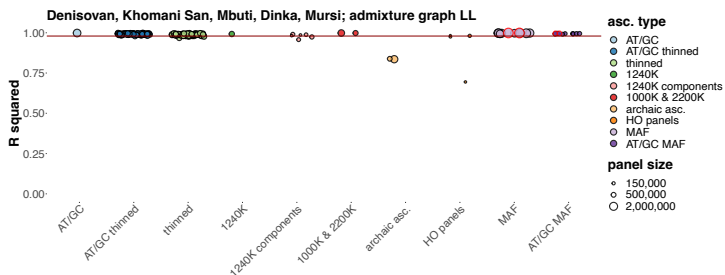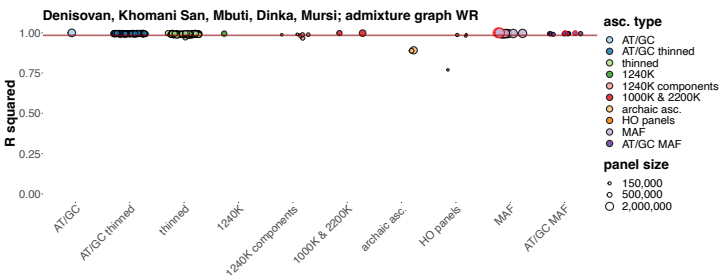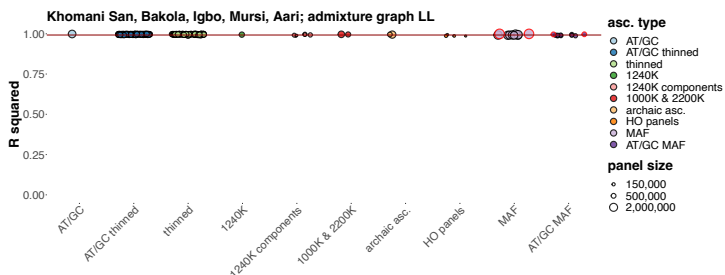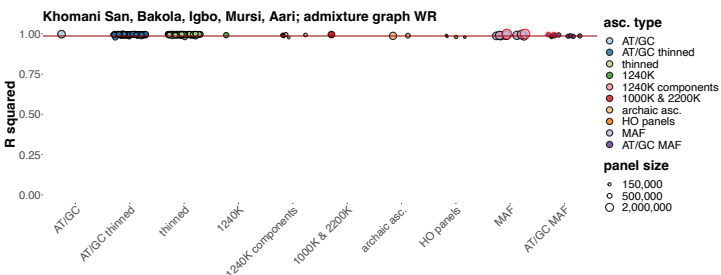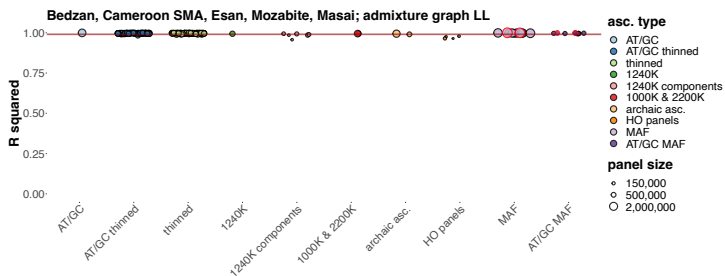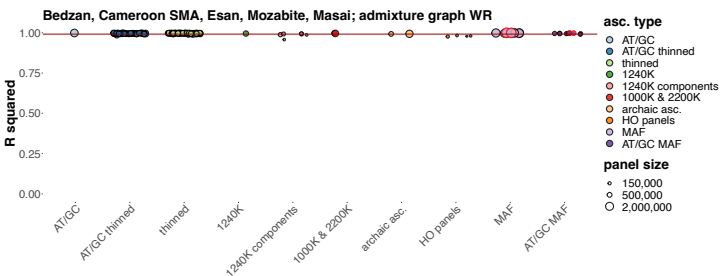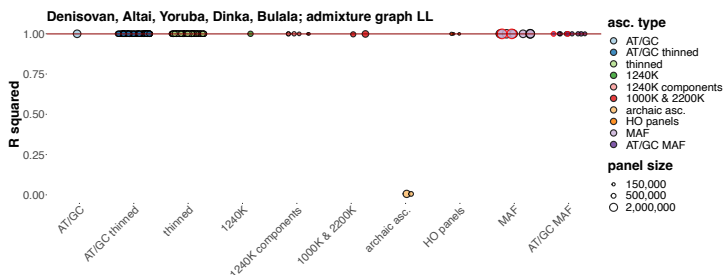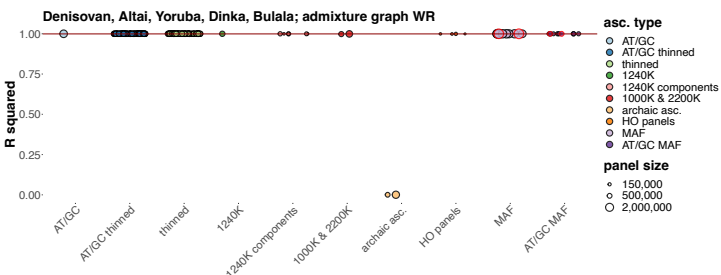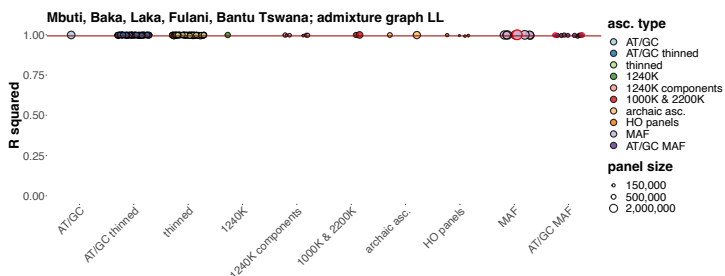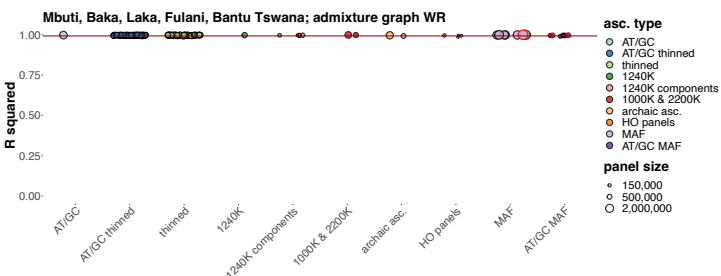

b

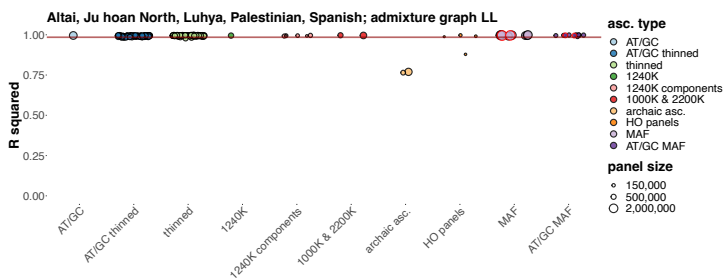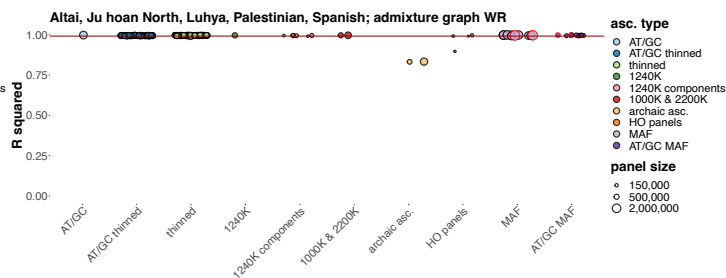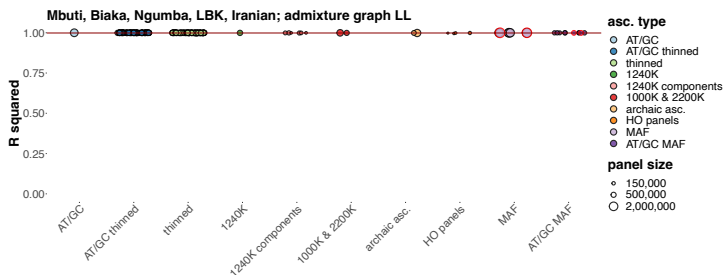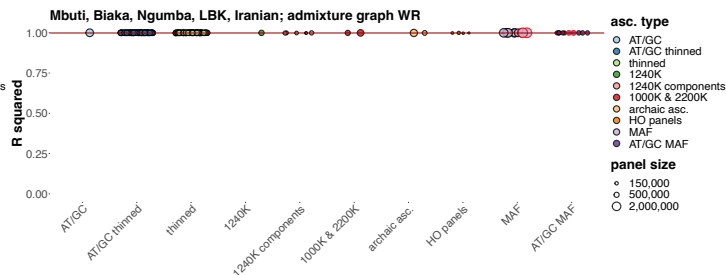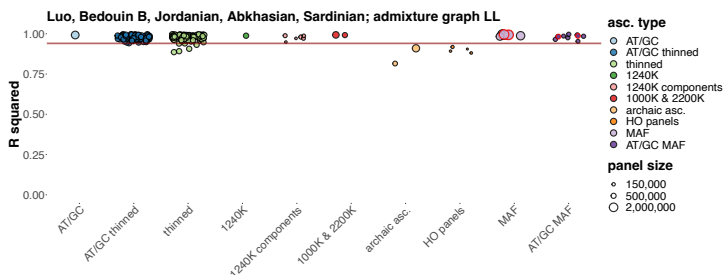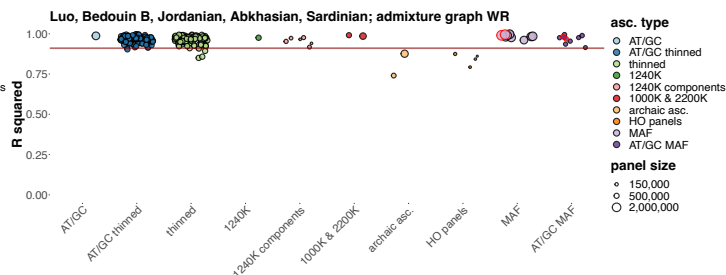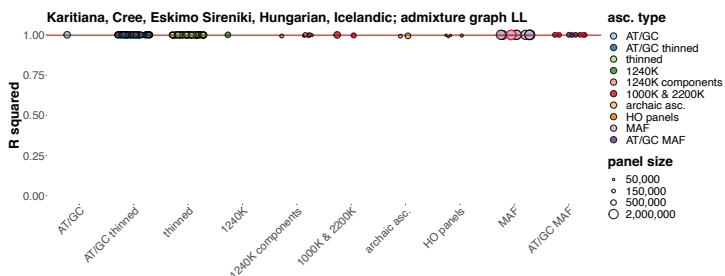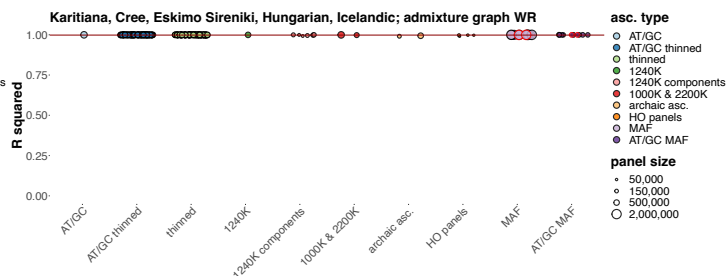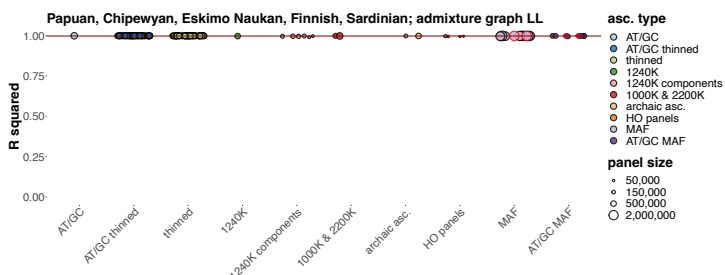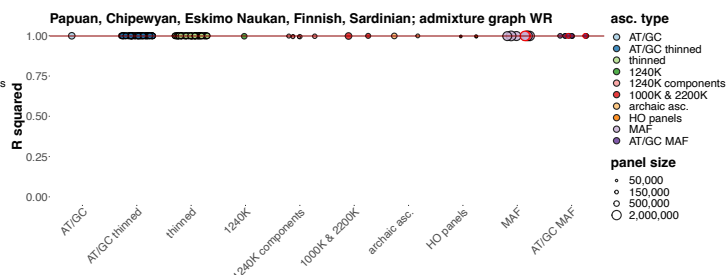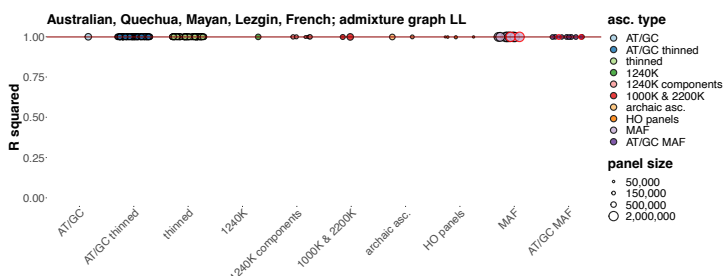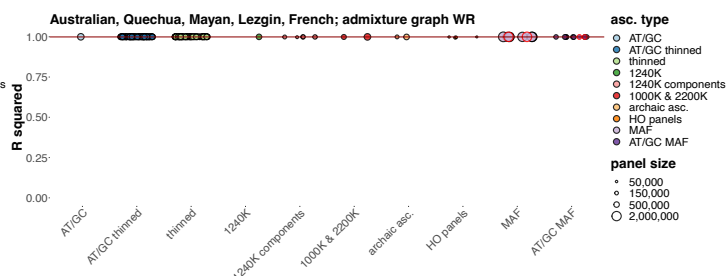

Supplement: S3 Fig — Correlation of admixture graph log-likelihood scores (LL) or worst f4-statistic residuals (WR) was explored for graphs fitted to unascertained vs. ascertained datasets. Results are shown for twelve population combinations indicated in plot titles (panels a, b). Results for ascertainment on variants common in Africans (either those having no detectable West Eurasian ancestry or on all Africans in the SGDP dataset) are circled in red. As a starting point for generating different ascertainments, we used either 11,706,773 sites (with no missing data at the group level) polymorphic in a set of 48 archaic and African groups composed of 97 individuals, or 10,051,585 such sites in 59 archaic, African, European, and Middle Eastern groups composed of 120 individuals, or 5,296,653 such sites in 51 Papuan, Native American/Siberian, European, Anatolian, and Caucasian groups composed of 112 individuals (S1 Table). Thirty eight site subsampling schemes were explored (see a list in the legend for Fig 2). The size of the resulting SNP panels is coded by point size, and ten broad ascertainment types are coded by color according to the legends. R2 values for LL are plotted in the left-hand panels, and R2 values for WR are plotted in the right-hand panels. The 2.5th LL or WR percentiles of all the thinned replicates combined, including those on all sites and AT/GC sites, are marked by brown lines. (PDF) [file pgen.1010931.s003.pdf]

**a**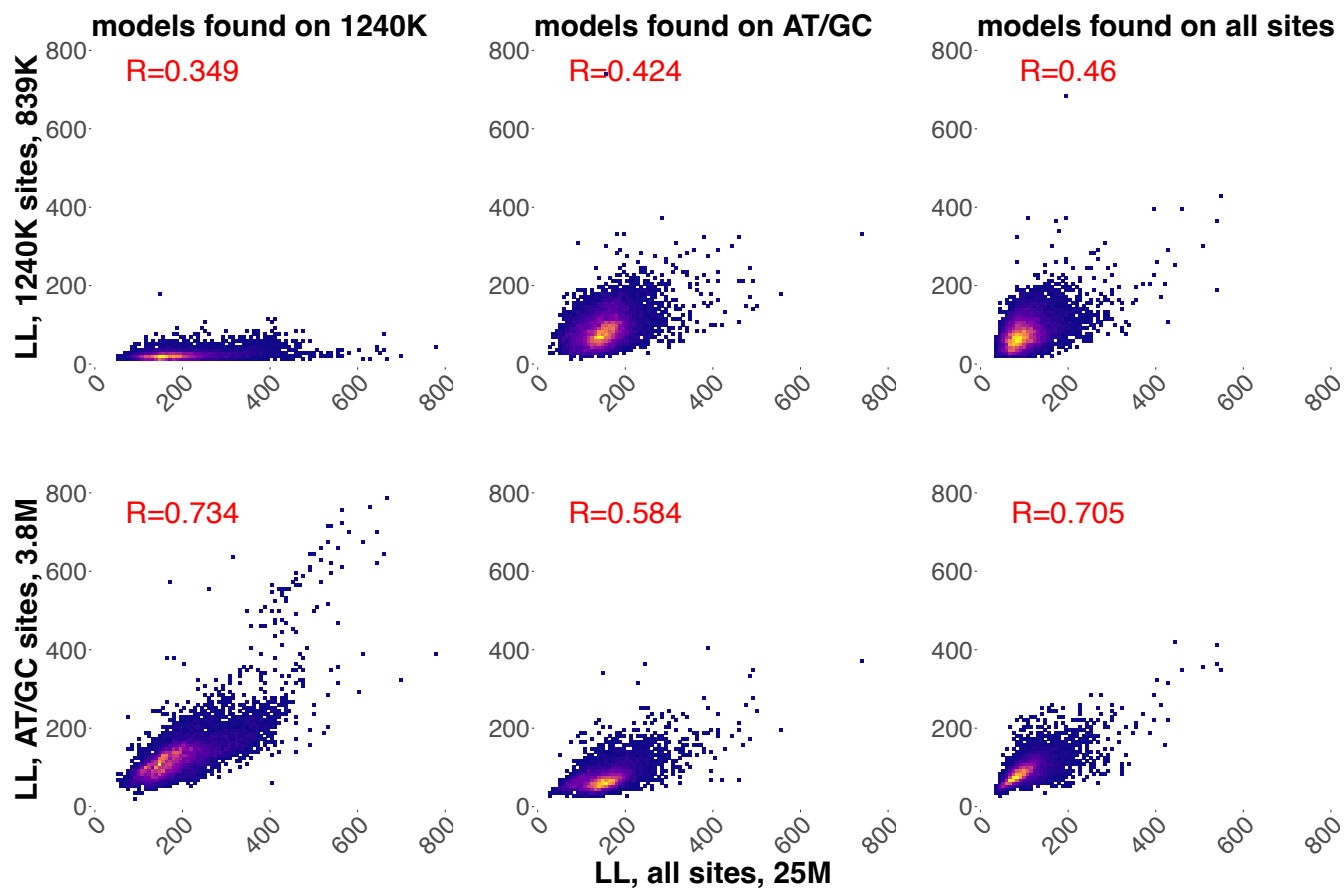**b**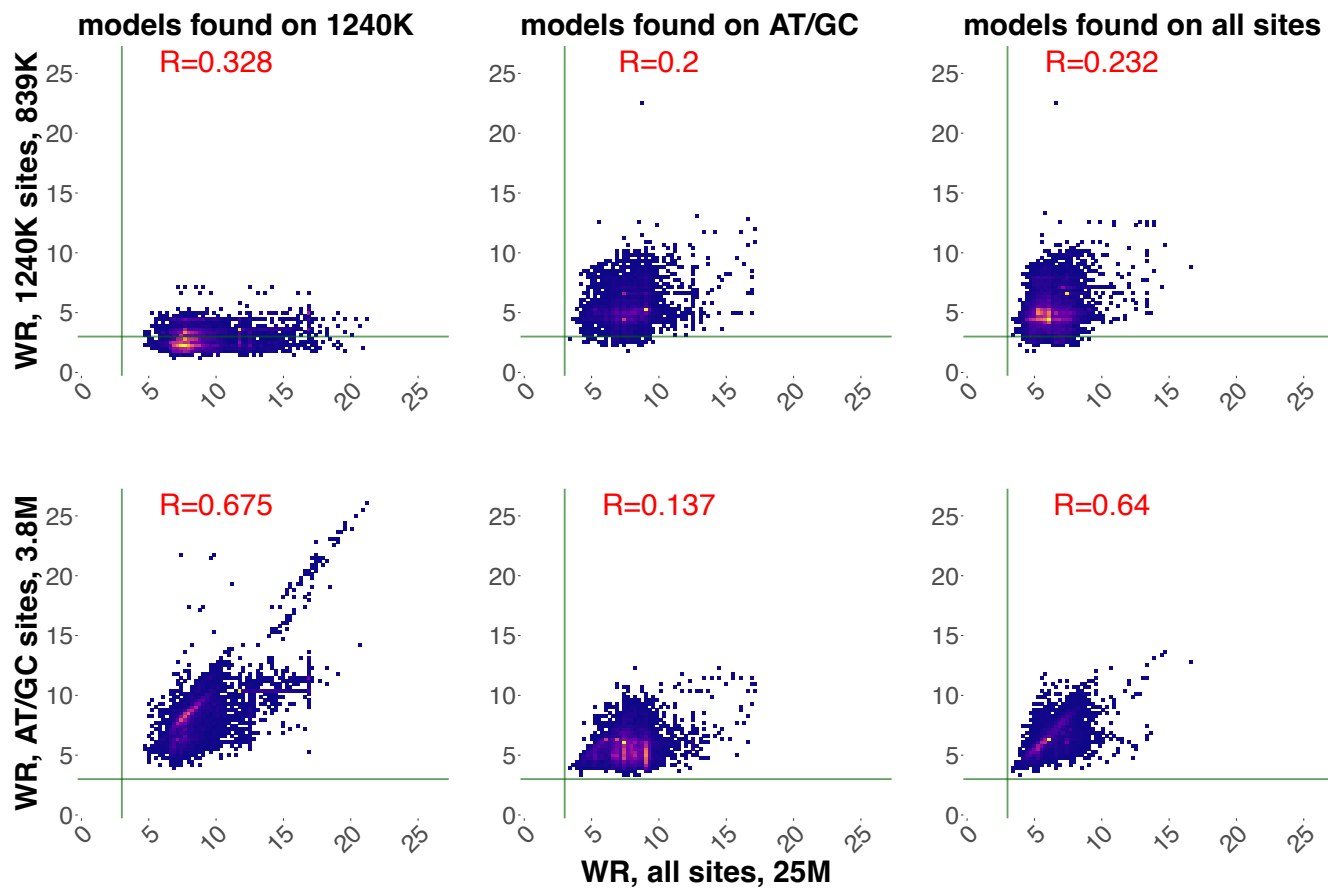

c

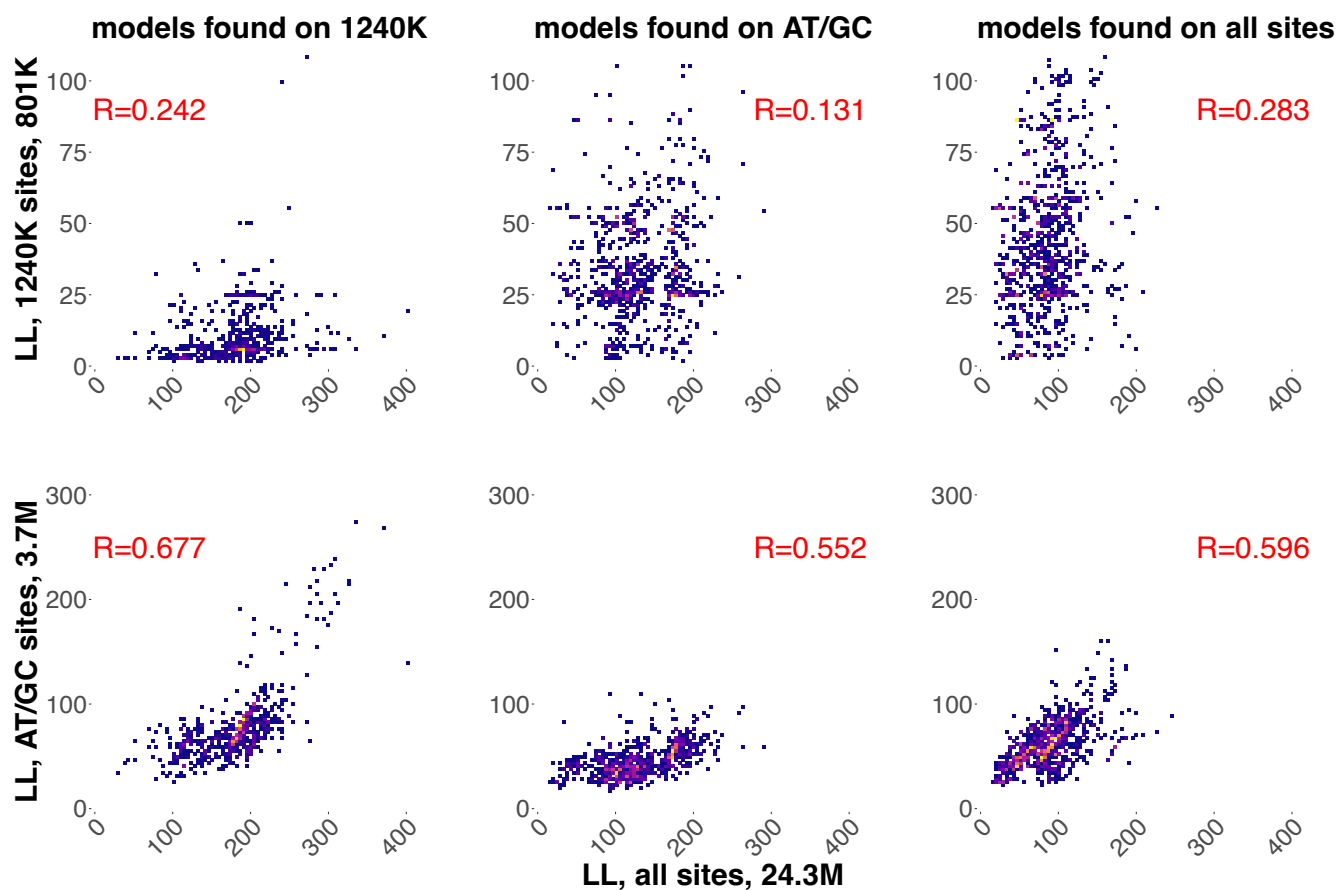

d

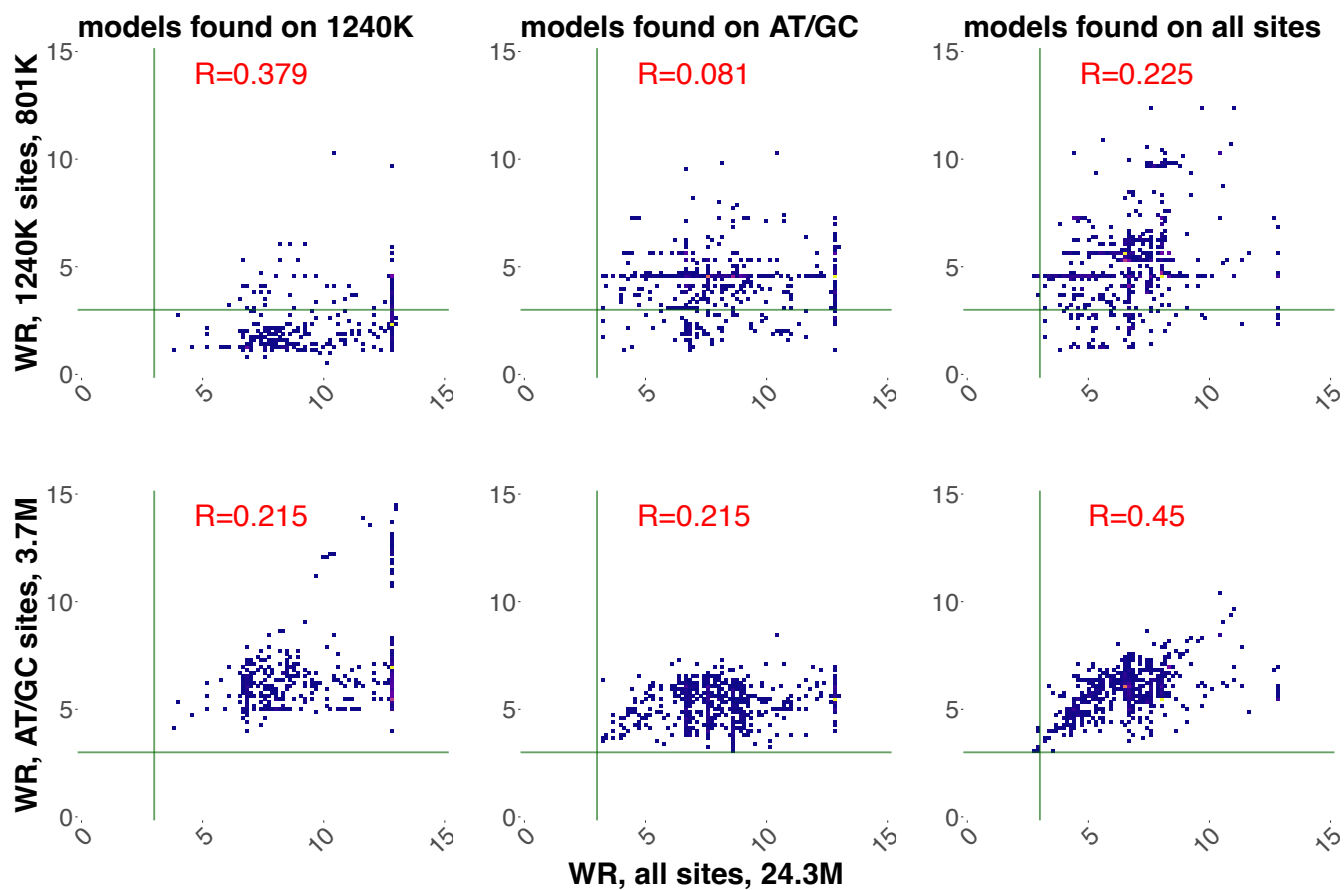

Supplement: S5 Fig — (a, b) Between 9,927 and 9,990 unique topologies including 10 groups and 8 admixture events were outcomes of 10,000 independent runs of the findGraphs algorithm on three datasets (shown in three columns). (c, d) Between 779 and 971 unique topologies including 7 groups and 4 admixture events were outcomes of 2,000 independent runs of the findGraphs algorithm on the three datasets. On x-axes LL or WR of admixture graphs fitted to all sites are shown. LL or WR of admixture graphs fitted to the 1240K SNP set are shown in the upper row on the y-axes, and LL or WR of admixture graphs fitted to AT/GC sites are shown in the lower row. Pearson correlation coefficients for these two sets of admixture graph fit metrics are displayed beside each plot in red. The WR threshold used often for fitting models (3 SE) is marked with green vertical and horizontal lines. (PDF) [file pgen.1010931.s005.pdf]

**a**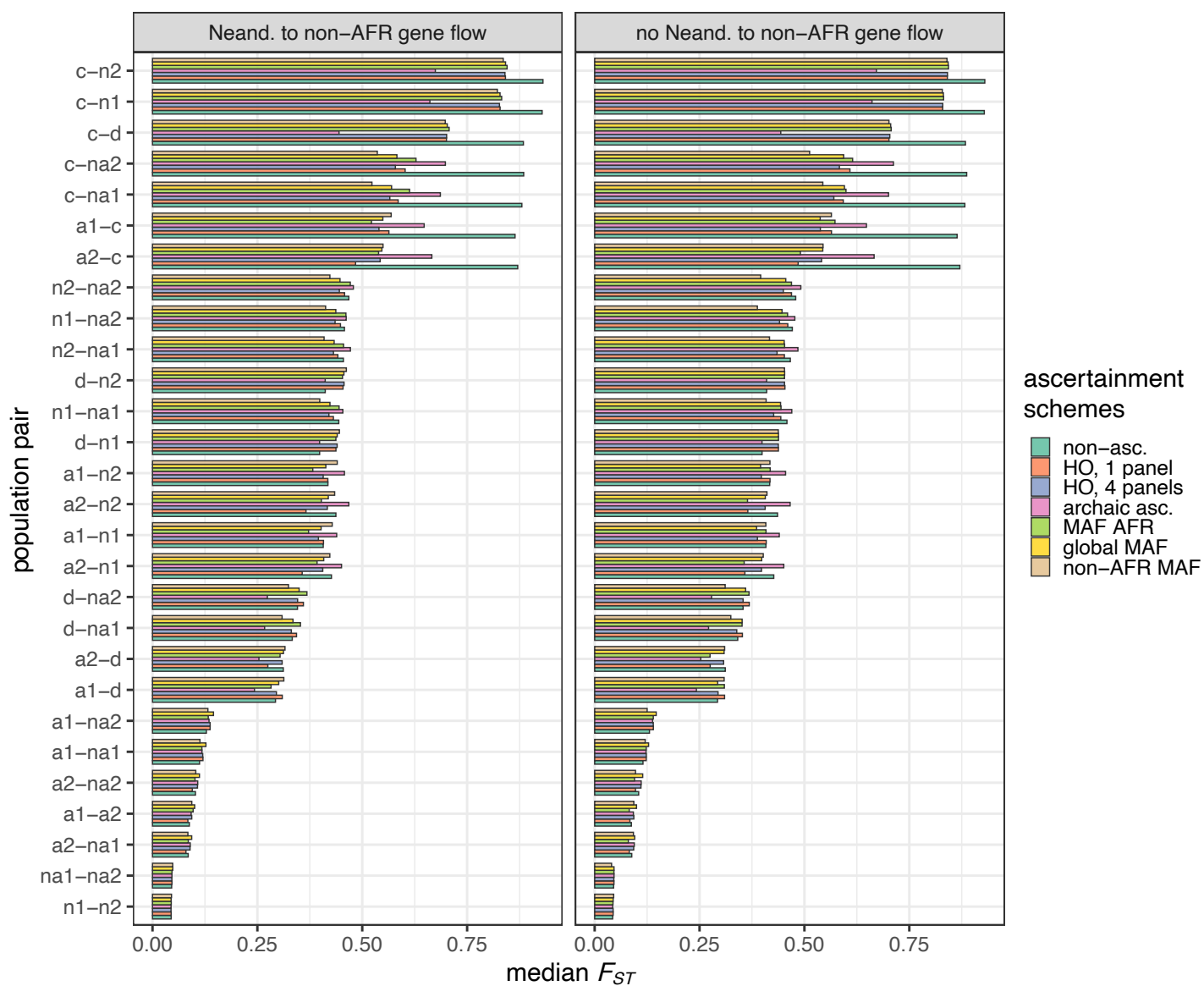

**b**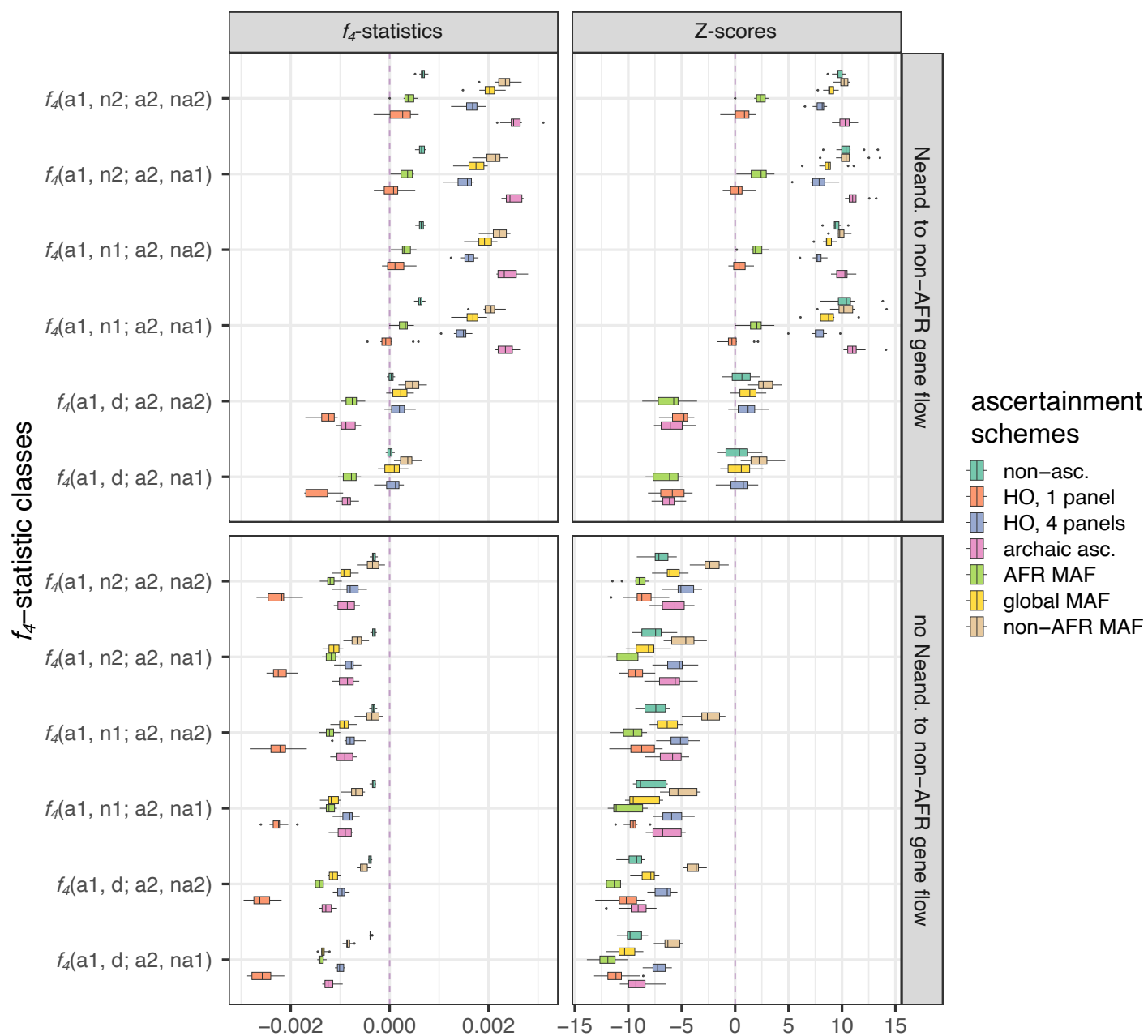

c

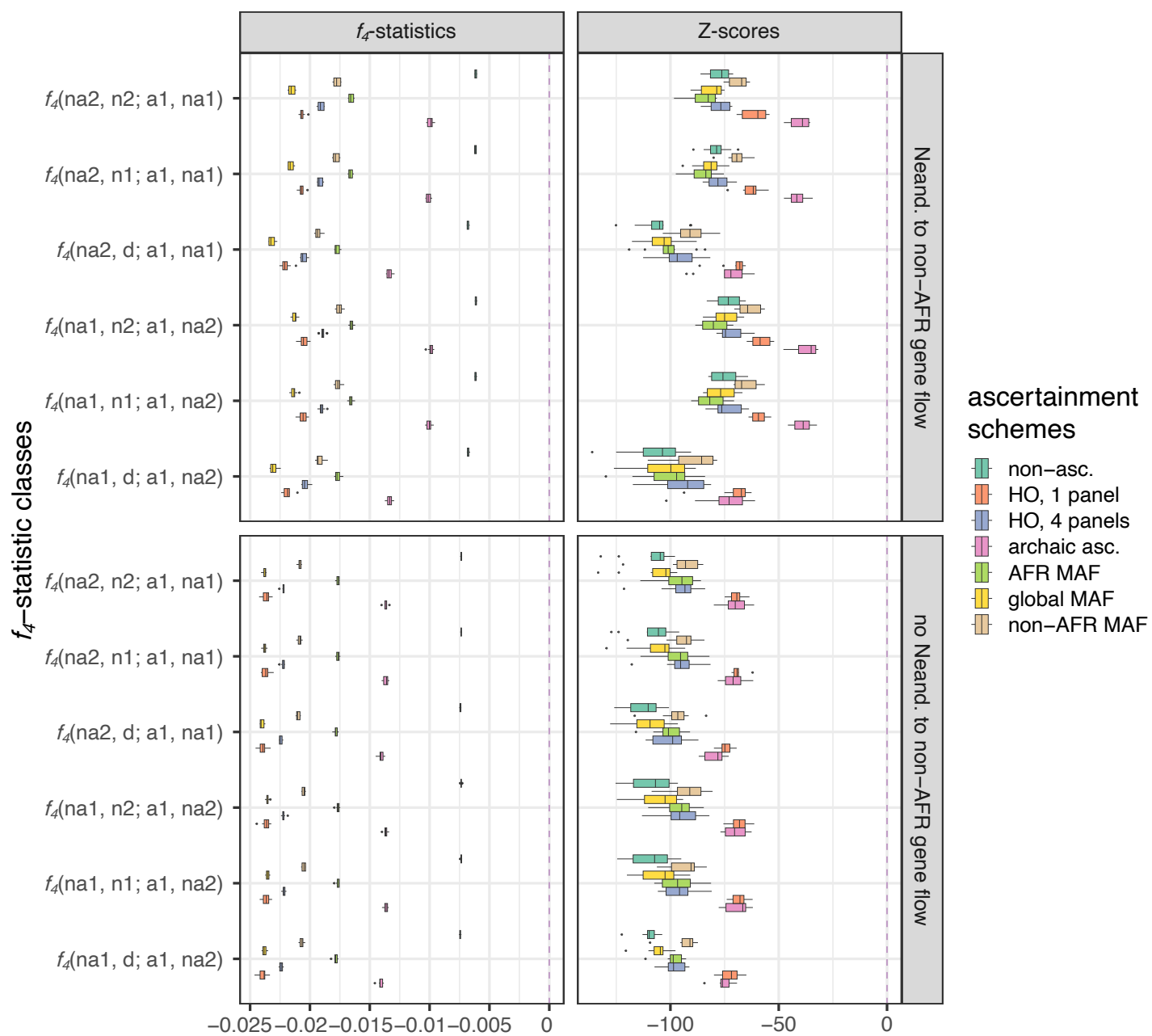

d

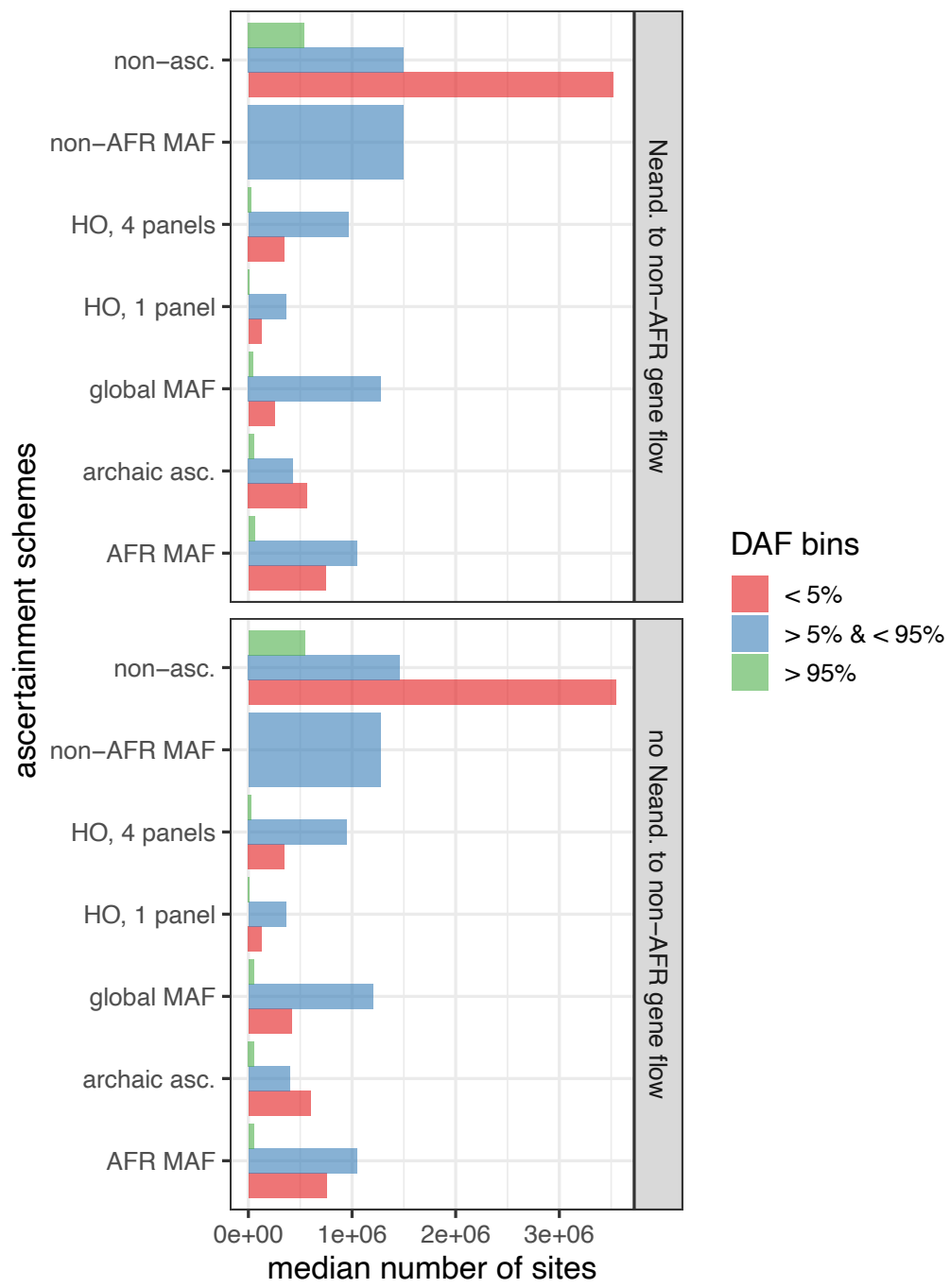

e

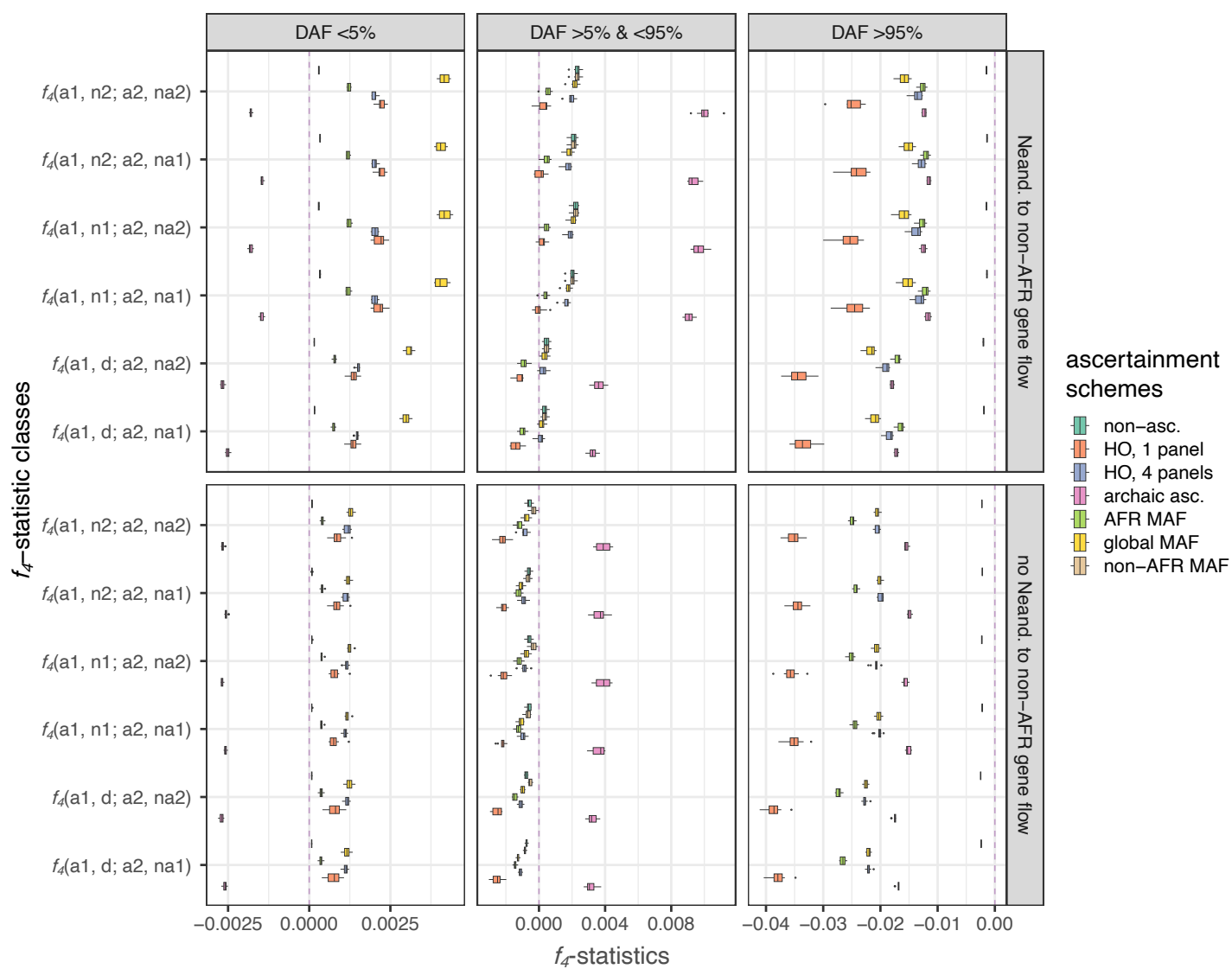

f

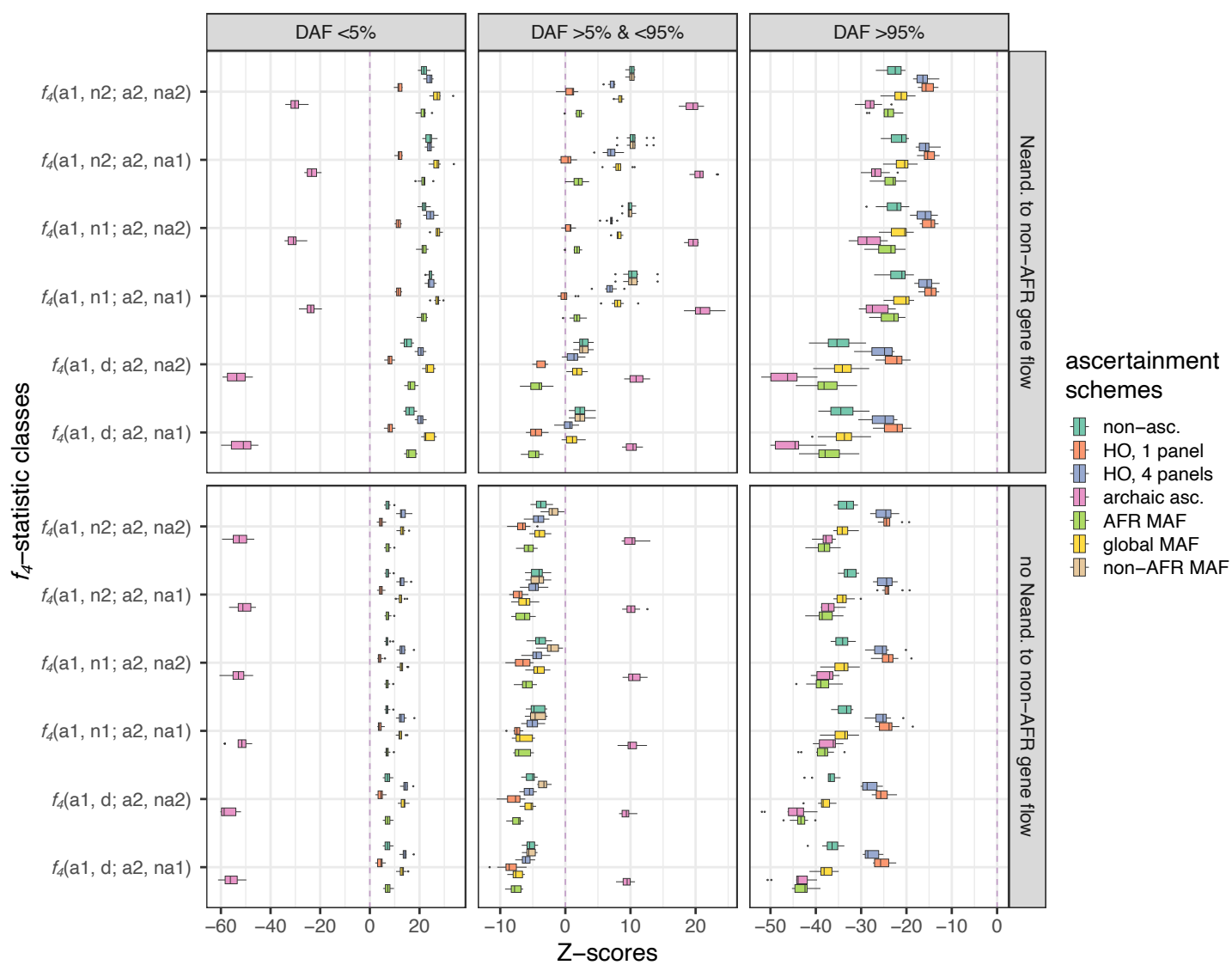

Supplement: S6 Fig — (a) Influence of ascertainment on FST across all population pairs on data simulated with or without the “Neanderthal” admixture in “non-Africans”. Median FST values are shown across 10 simulation iterations. Results for seven types of SNP sets are presented: 1) unascertained sites (on average 5.55M polymorphic sites without missing data); 2) HO one-panel ascertainment, based on the “African 2” group (500K sites on average across simulation iterations); 3) HO four-panel ascertainment (on the “African 1”, “African 2”, “non-African 1”, and “non-African 2” groups, 1.34M sites on average); 4) archaic ascertainment (1.05M sites on average); 5) AFR MAF ascertainment, that is restricting to sites with MAF >5% in the union of “African 1” and “African 2” groups (1.66M sites on average); 6) global MAF ascertainment on the union of “African 1”, “African 2”, “non-African 1”, “non-African 2” (2.75M sites on average); 7) non-African MAF ascertainment (1.04M sites on average). (b) Boxplots summarizing various f4-statistics of the form f4(“African 1”, “archaic”; “African 2”, “non-African”) for the two simulated topologies, on unascertained and ascertained data across 10 simulation runs. f4-statistics are shown on the left and their Z-scores are shown on the right. (c) Boxplots summarizing various f4-statistics of the form f4(“non-African 1 or 2”, “archaic”; “African 1”, “non-African 2 or 1”) for the two simulated topologies, on unascertained and ascertained data across 10 simulation runs. f4-statistics are shown on the left and their Z-scores are shown on the right. (d) Simplified derived allele frequency (DAF) spectra for the unascertained and ascertained datasets. DAF was defined on the union of groups “non-African 1” and “non-African 2”, and three allele frequency bins were defined: <5%, > = 5% & < = 95%, >95%. Median site counts across 10 simulation iterations are presented. Similar results on real data are shown in S10 Table. (e and f) Boxplots summarizing DAF-stratified f4-sta [file pgen.1010931.s006.pdf]

**a**

**a.** Sim=1; nleaf=8; nadmix=4

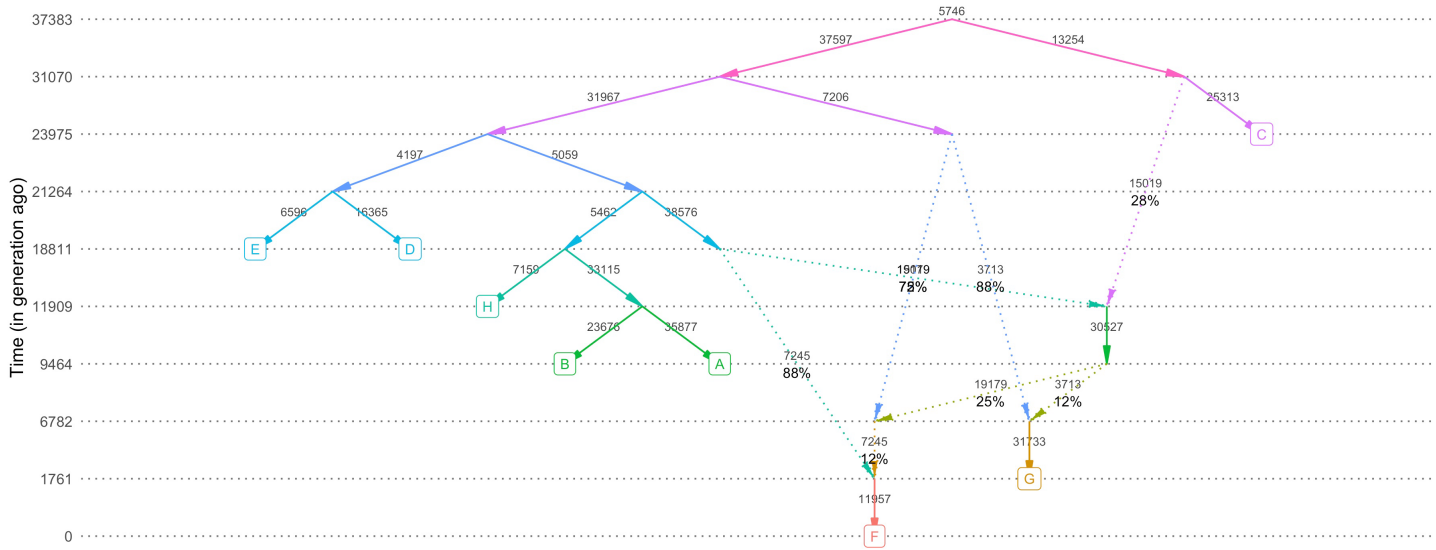

**b.**

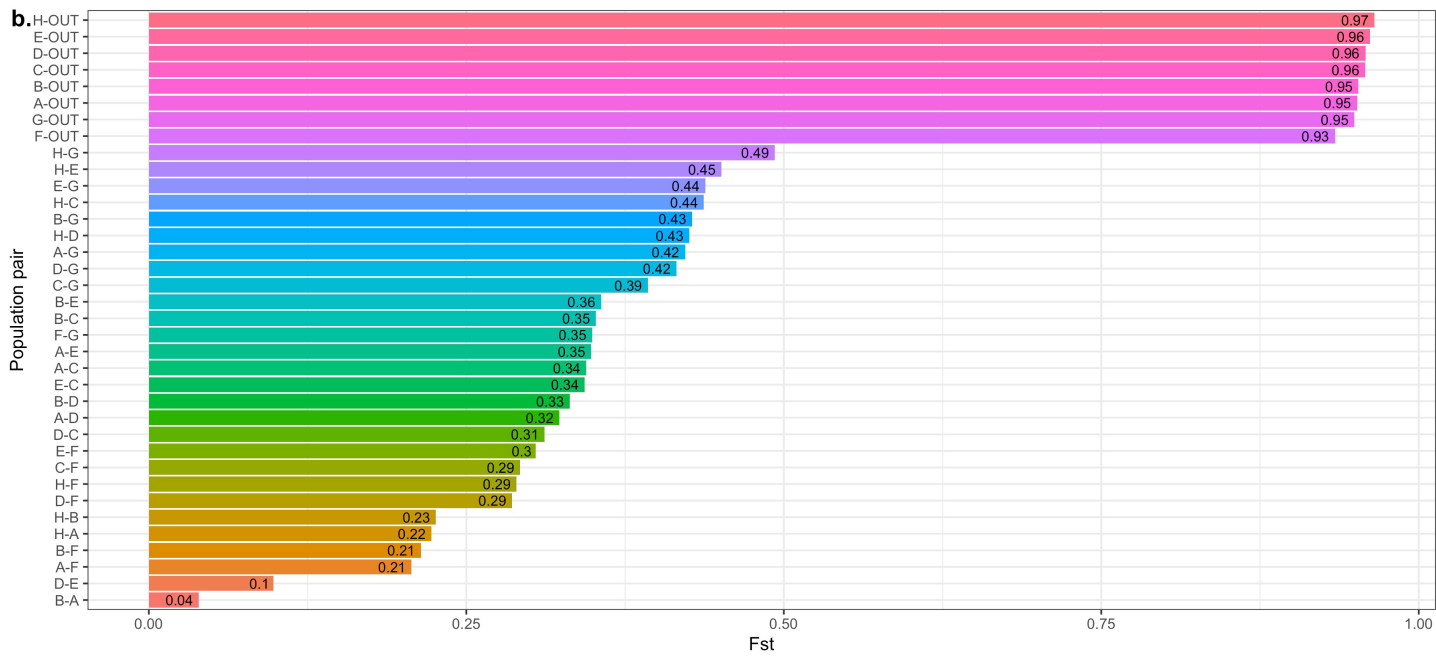

**b**

**a.** Sim=1; nleaf=8; nadmix=5

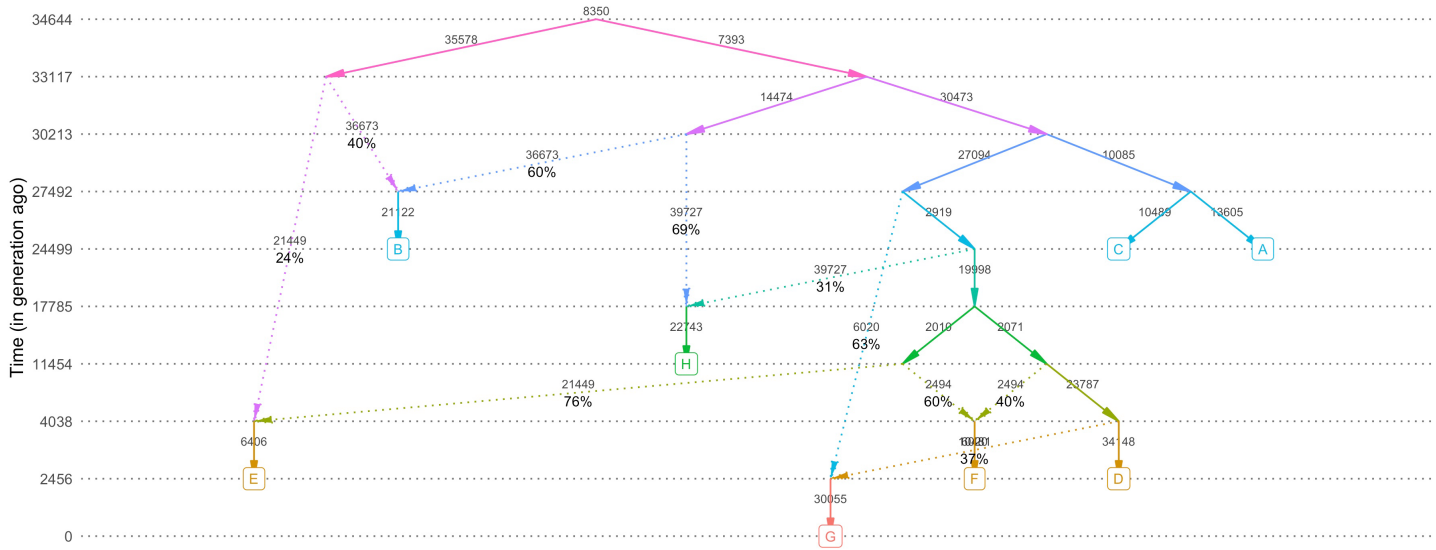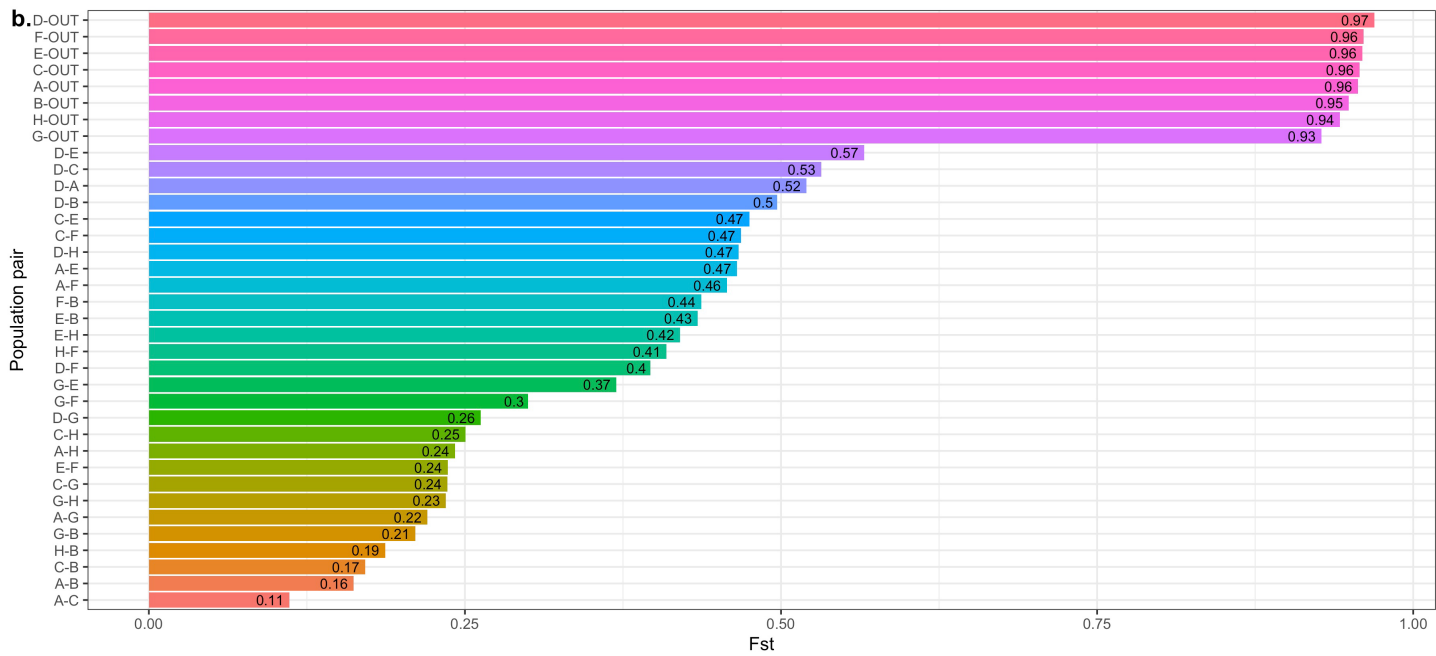

**C**

**a.** Sim=1; nleaf=9; nadmix=4

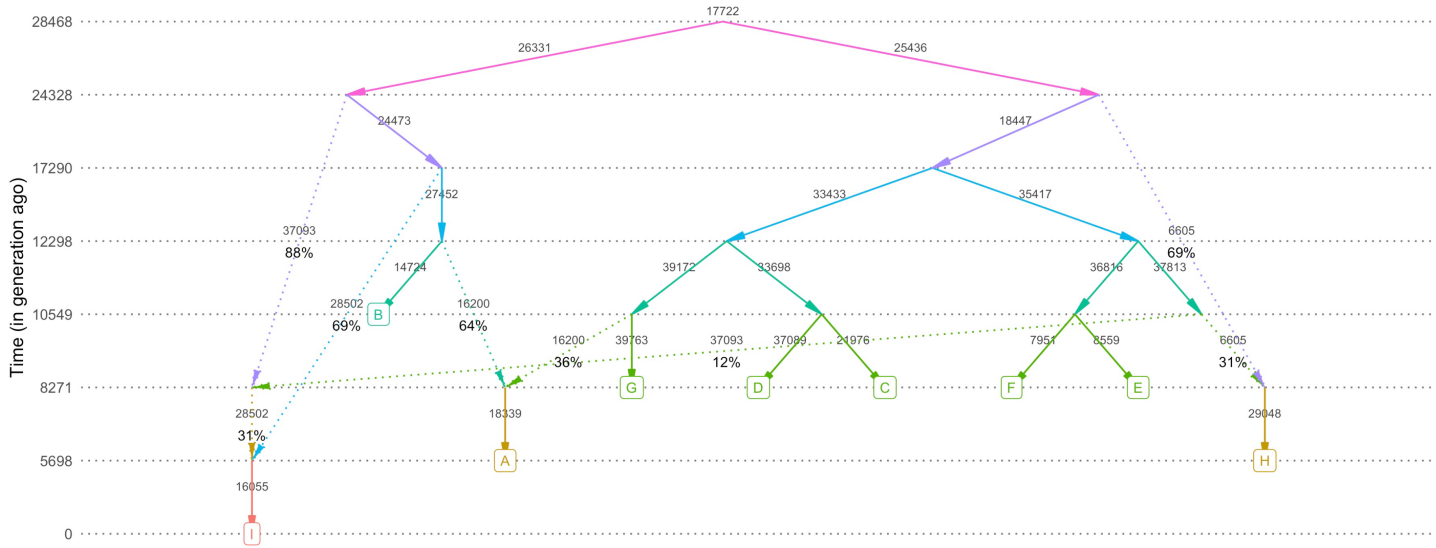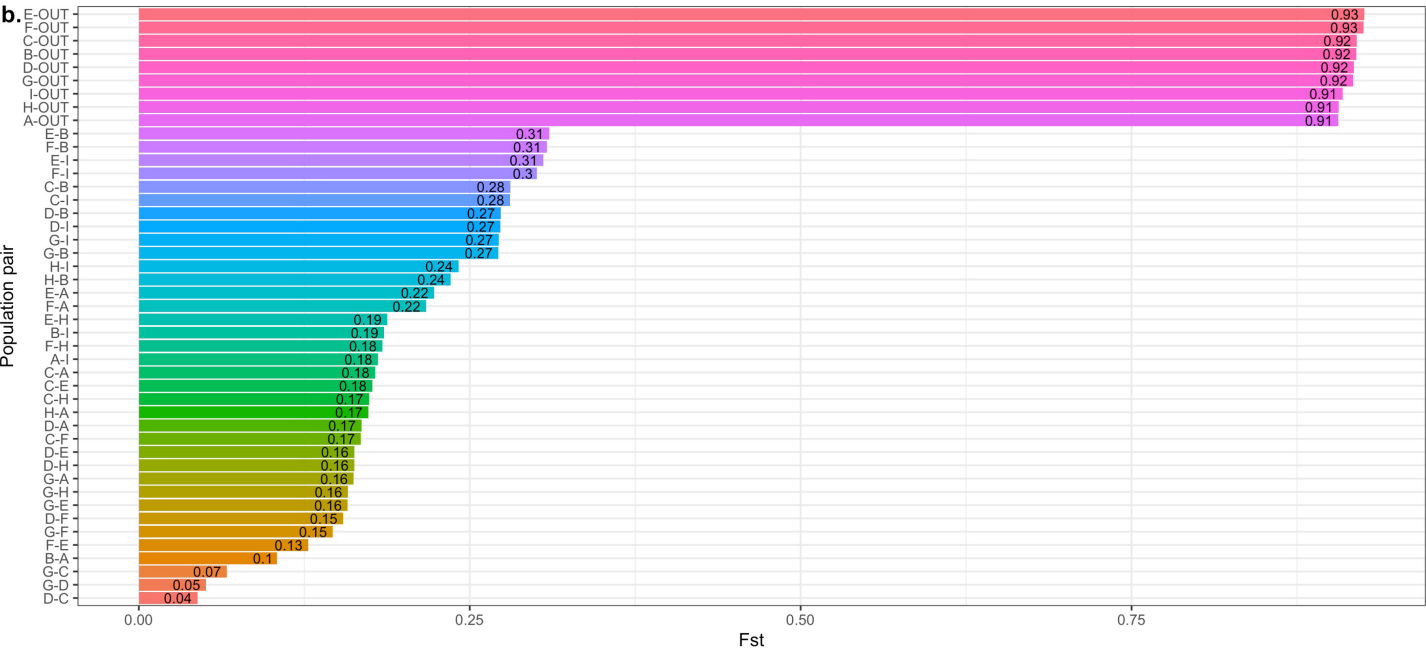

d

a. Sim=1; nleaf=9; nadmix=5

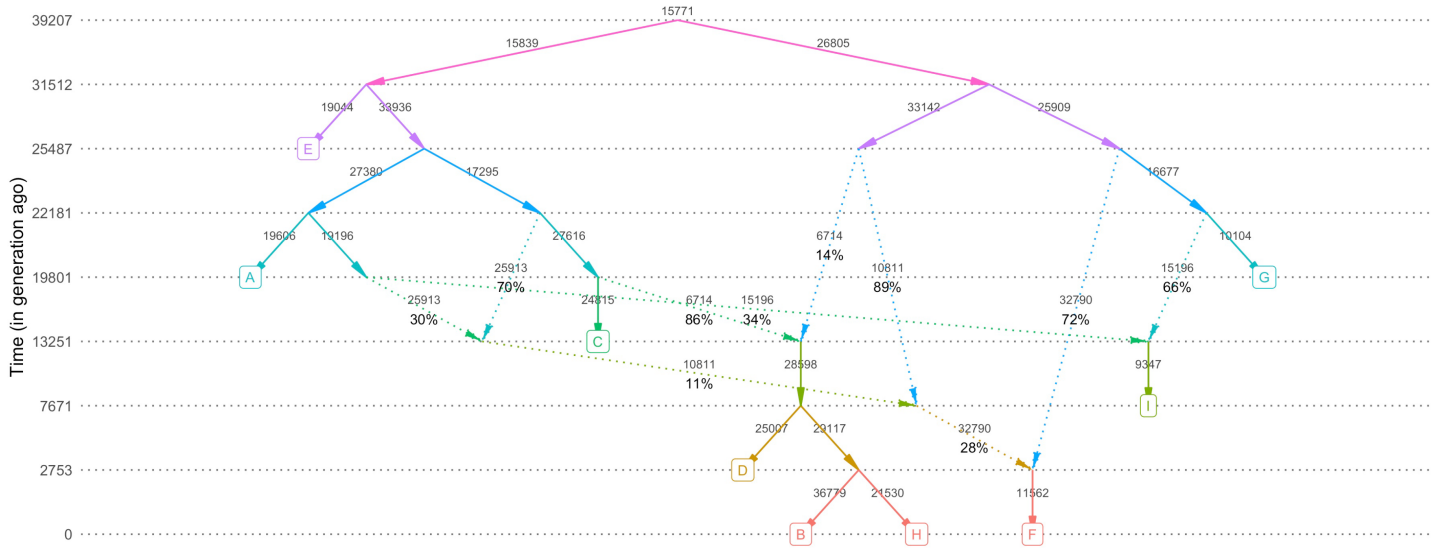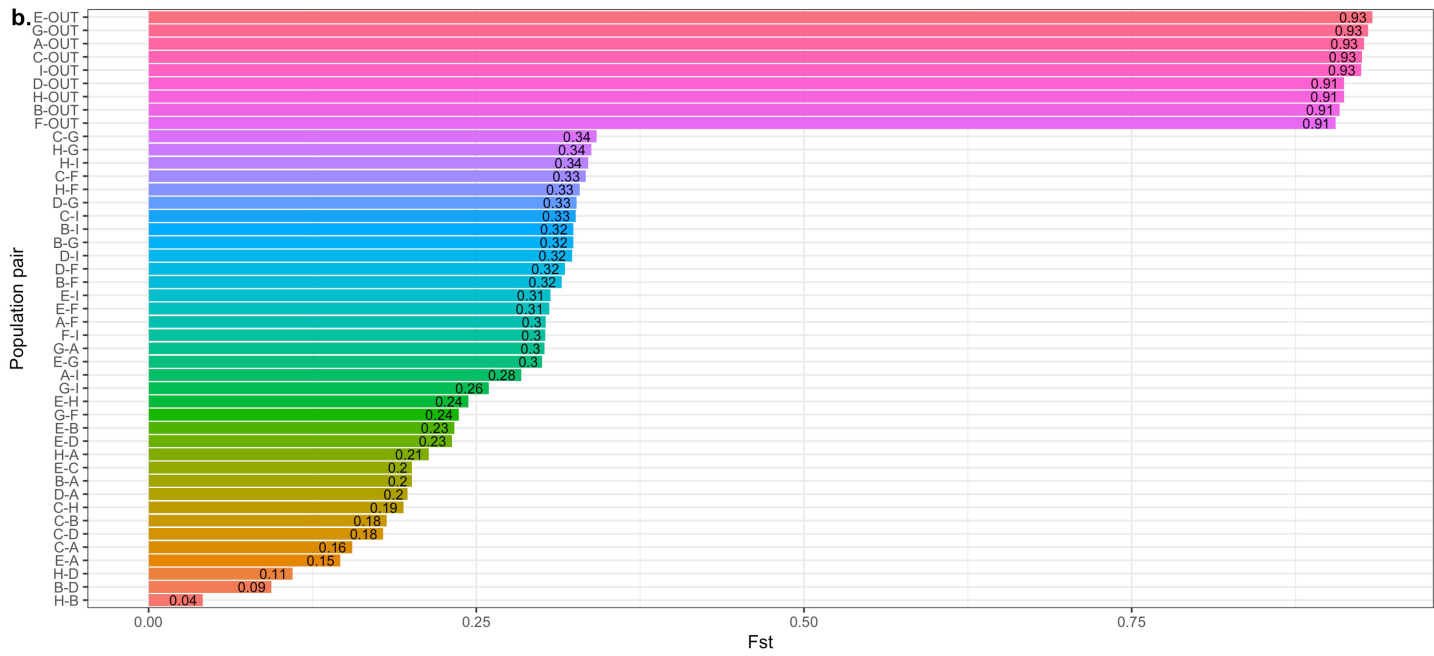

Supplement: S7 Fig — One topology per graph complexity class is shown as an example, along with FST values for all population pairs. Time in generations is shown on the y-axis (for visual clarity, points on this axis are not spaced proportionately). Effective population sizes (in diploid individuals) are shown beside each edge. Sampled populations are labelled by letters in squares. Outgroups are not visualized; their divergence was placed at 40,000 generations ago for all the simulated histories, and their effective population size was 100,000 or alternatively 1,000 diploid individuals (both series of simulations were prepared). The color gradients have no special meaning and are used for visual clarity only. (PDF) [file pgen.1010931.s007.pdf]

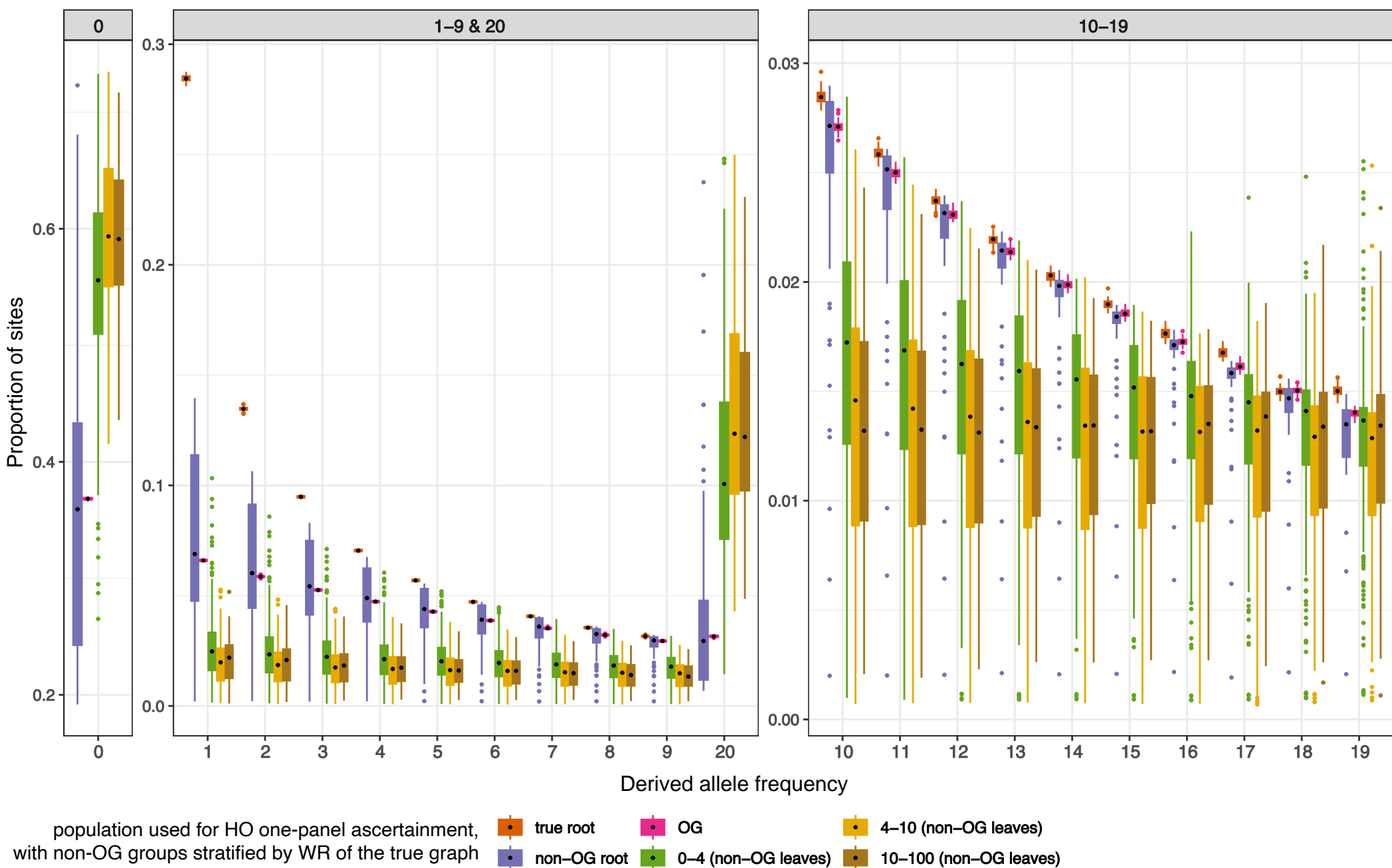

Supplement: S8 Fig — The spectra shown here are based only on sites polymorphic in a sample of 20 chromosomes drawn at the root of the simulation. We note that outgroups had an effective population size of 100,000 diploid individuals and were included in the fitted admixture graphs; in other words, they were co-analyzed with the other populations. Populations sampled at branch tips ("non-OG groups”) are binned by worst f4-statistic residual (WR) of the true graph under HO one-panel ascertainment based on that population: 0 to 4, 4 to 10, and 10 to 100 SE. The boxplots summarize DAF across all the simulated admixture graph topologies. DAF bins are shown in three separate panels with different y-axis ranges: 0 derived alleles; 1 to 9 and 20 derived alleles; 10 to 19 derived alleles. (PDF) [file pgen.1010931.s008.pdf]

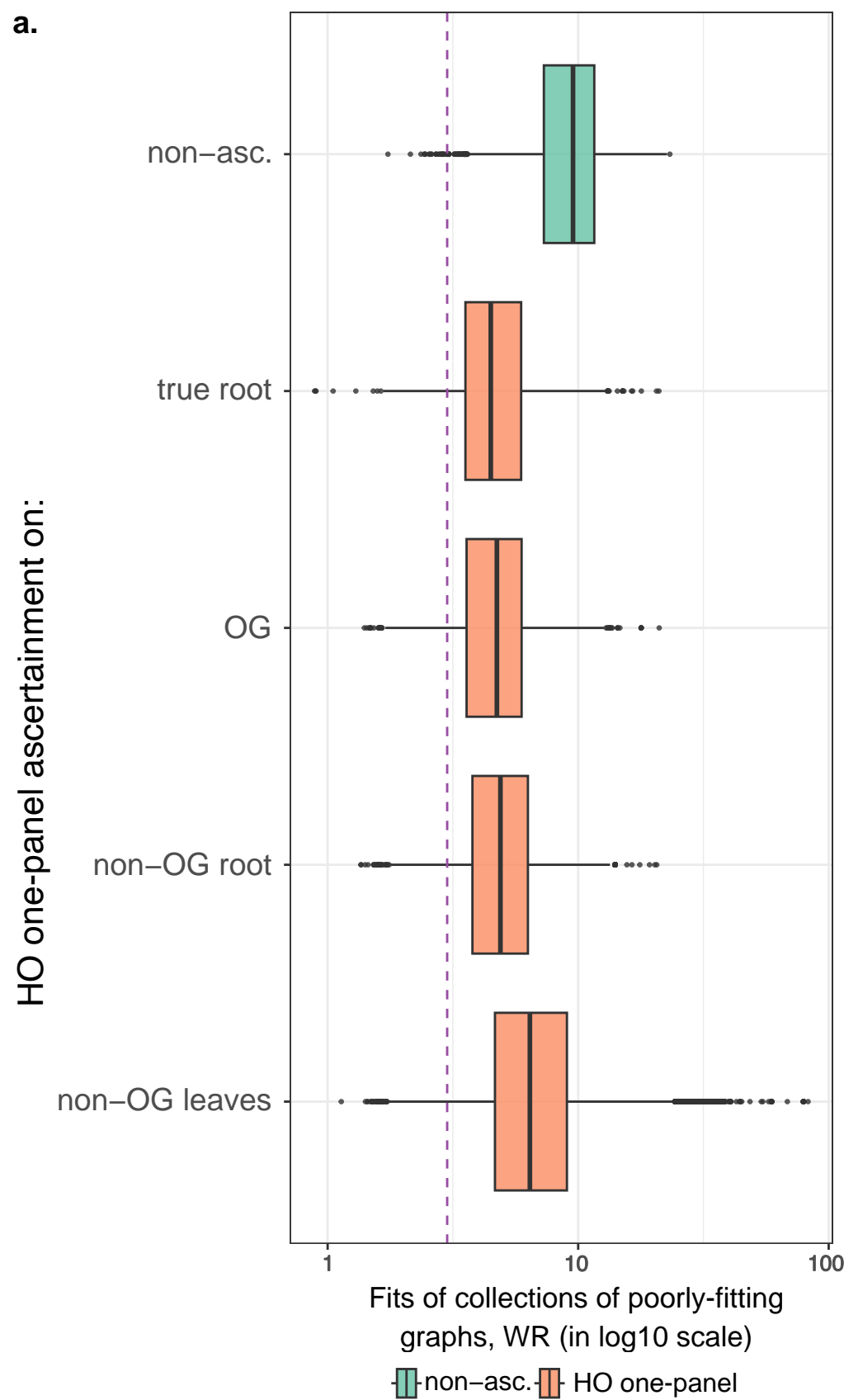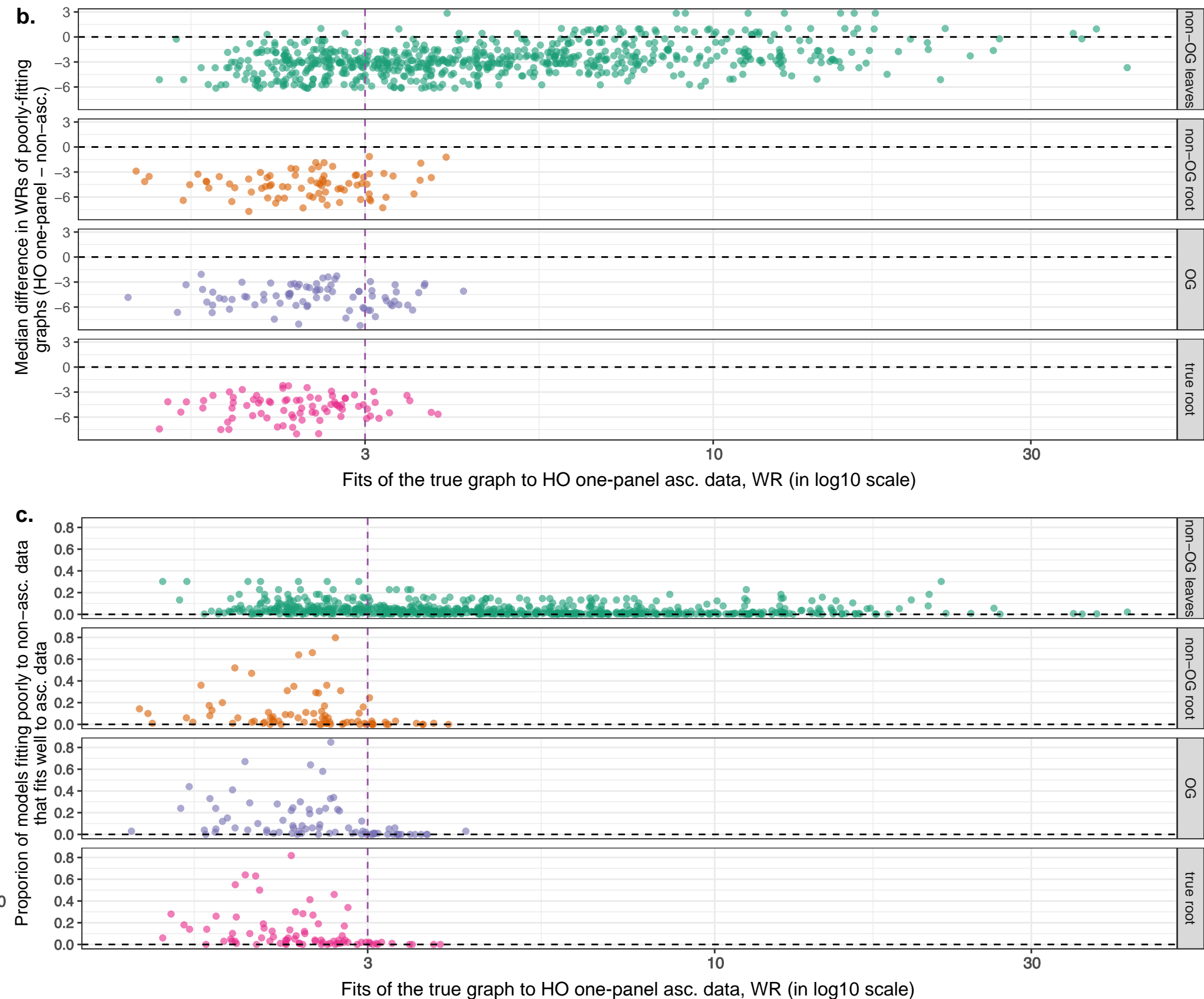

Supplement: S9 Fig — A set of 100 topologically diverse poorly-fitting graphs was generated for each simulated random topology (see Methods) and fitted to both non-ascertained data and to all SNP sets ascertained using the HO one-panel scheme. (a) Boxplots summarizing distributions of worst f4-statistic residuals (WR) of incorrect graphs fitted to datasets of several types: non-ascertained, ascertained on the outgroup with an effective population size of 100,000 that was co-modelled with the other populations (“OG”), on the root population sample that was not co-modelled with the other populations (“true root”), on the root of all non-outgroup populations that was not co-modelled with the other populations (“non-OG root”), and on non-outgroup populations co-modelled with the other populations (“non-OG leaves”). Results for all the simulated topologies were pooled. (b) SNP sets generated by HO one-panel ascertainment on simulated data in the “bias” vs. “power” coordinates. WR of the true simulated graph serves as a measure of ascertainment bias (on the x-axis), and median difference in WRs of 100 poorly-fitting graphs fitted to ascertained and non-ascertained data (on the y-axis) serves as a measure of power to reject incorrect models. Results are shown separately for SNP sets ascertained on OG, true root, non-OG root, and non-OG groups. Each dot represents an ascertained SNP set. (c) This series of plots is similar to that in panel b, but another measure of statistical power is used: the proportion of graphs which fit non-ascertained data poorly (WR >3 SE) but fit ascertained data well (WR <3 SE). (PDF) [file pgen.1010931.s009.pdf]

**a.**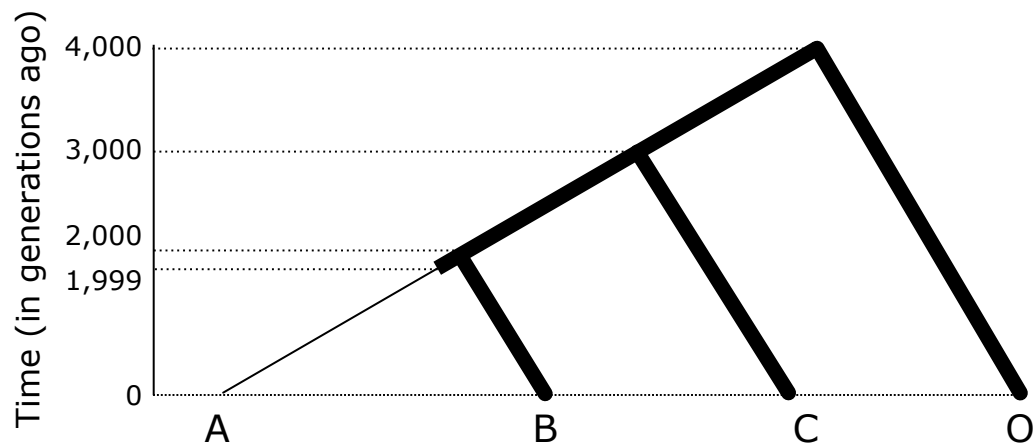**b.**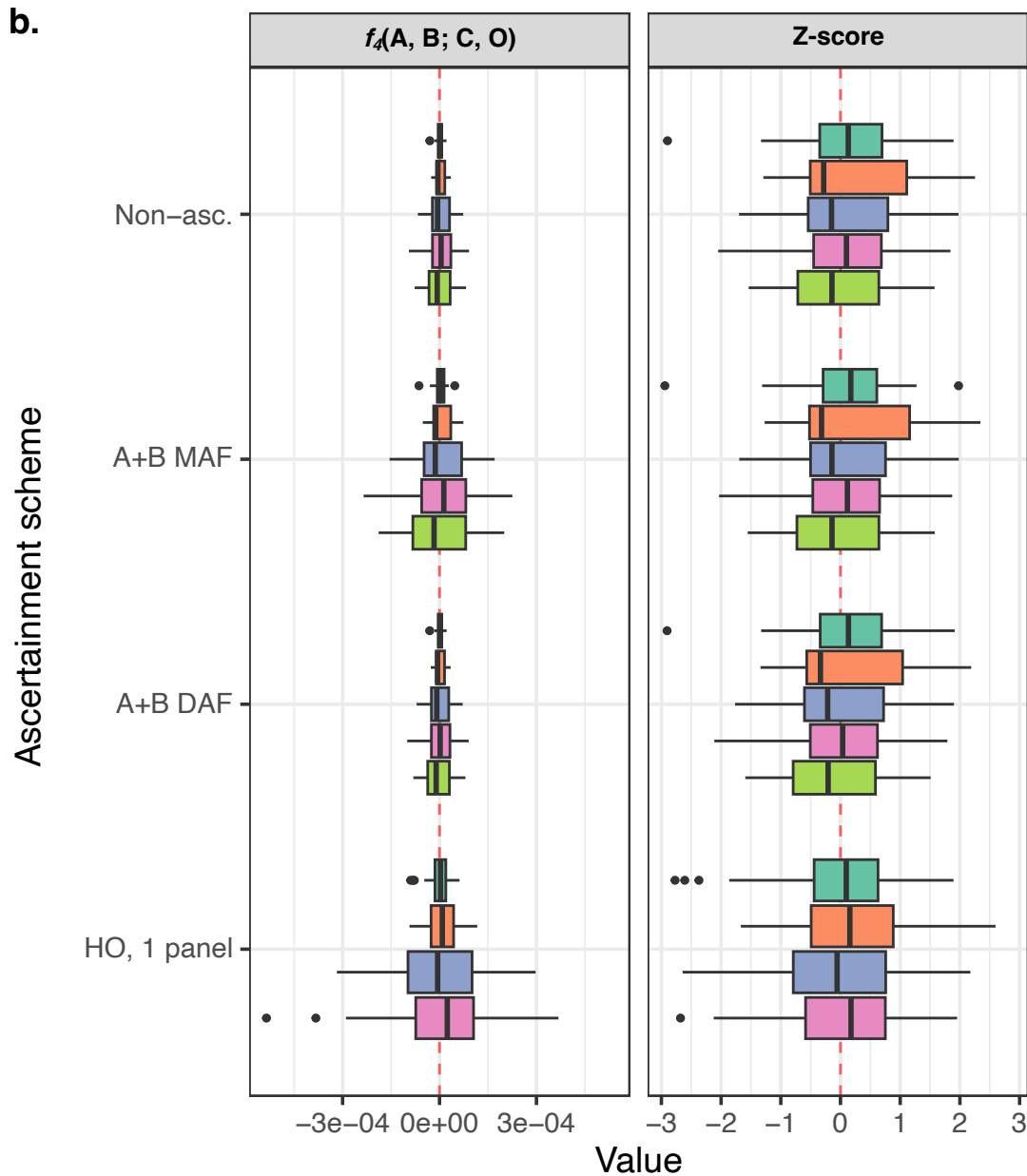

Population size  
after bottleneck

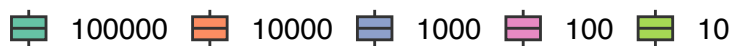

Supplement: S10 Fig — The influence of ascertainment on f4-statistic cladality tests in the case of a simple tree (O, (C, (A, B))) shown in (a). Effective population size was constant across the tree, except for a drop in group A’s size at 1,999 generations in the past: from 100,000 to 10,000, 1,000, 100, or 10 diploid individuals. There was also a set of control simulations without any reduction in effective population size. In panel (b), f4-statistics f4(A, B; C, O) and their Z-scores on 20 non-ascertained simulated datasets per bottleneck class and on datasets ascertained in various ways are summarized with boxplots. The ascertainment schemes are as follows: 1) keeping sites with MAF >5% in the union of groups A and B (abbreviated as “A+B MAF”); 2) keeping sites with derived allele frequency <95% in the union of groups A and B (abbreviated as “A+B DAF”); 3) HO one-panel ascertainment on either group A, B, C, or O (results pooled for all ascertainment groups are shown). Very similar results were obtained for other MAF (2.5%, 10%) and DAF thresholds (90%, 97.5%) used for ascertainment; these results are not shown for brevity. In the case of HO ascertainment, no results are shown for simulations with the smallest effective size of group A since too few SNPs were available due to rapid fixation of variants in the population with the extremely low effective size. (PDF) [file pgen.1010931.s010.pdf]

a

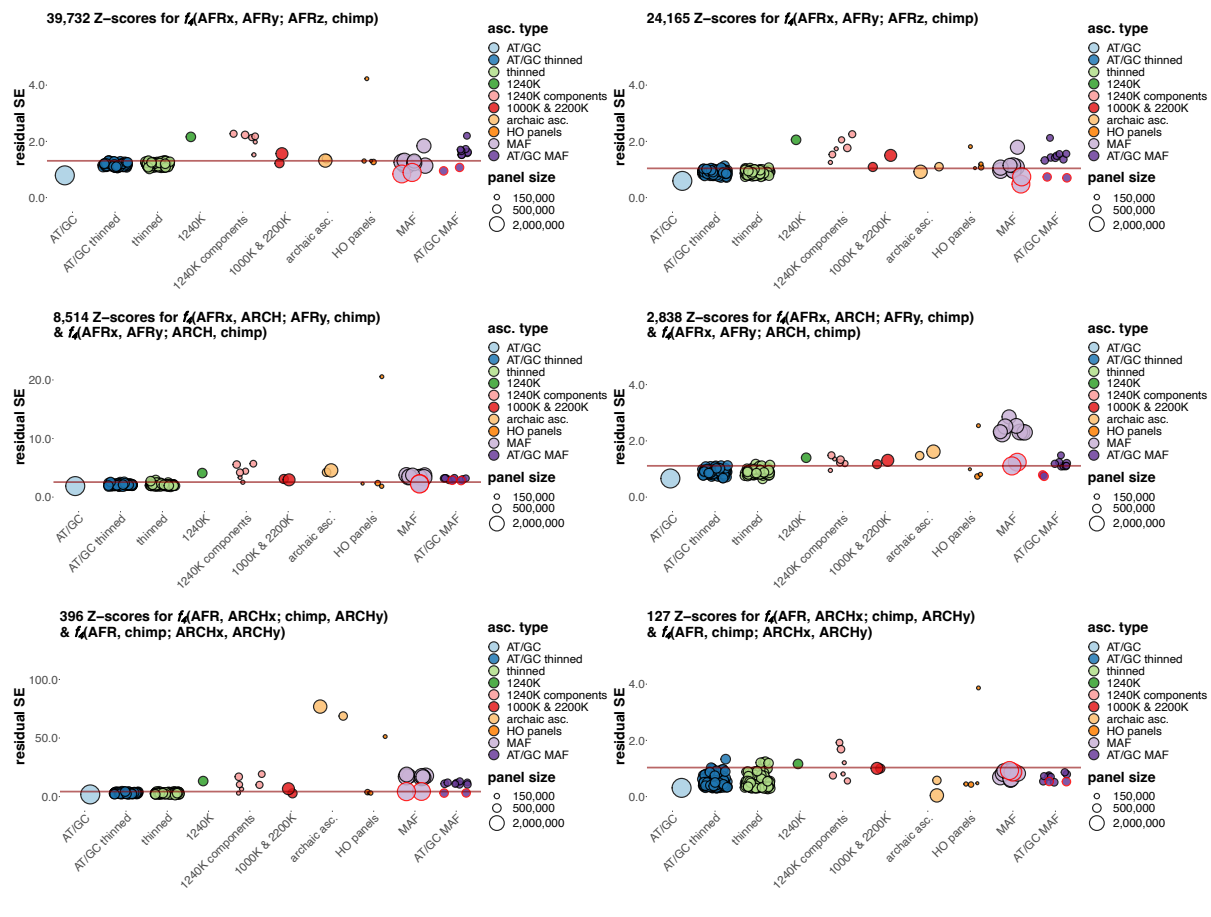

b

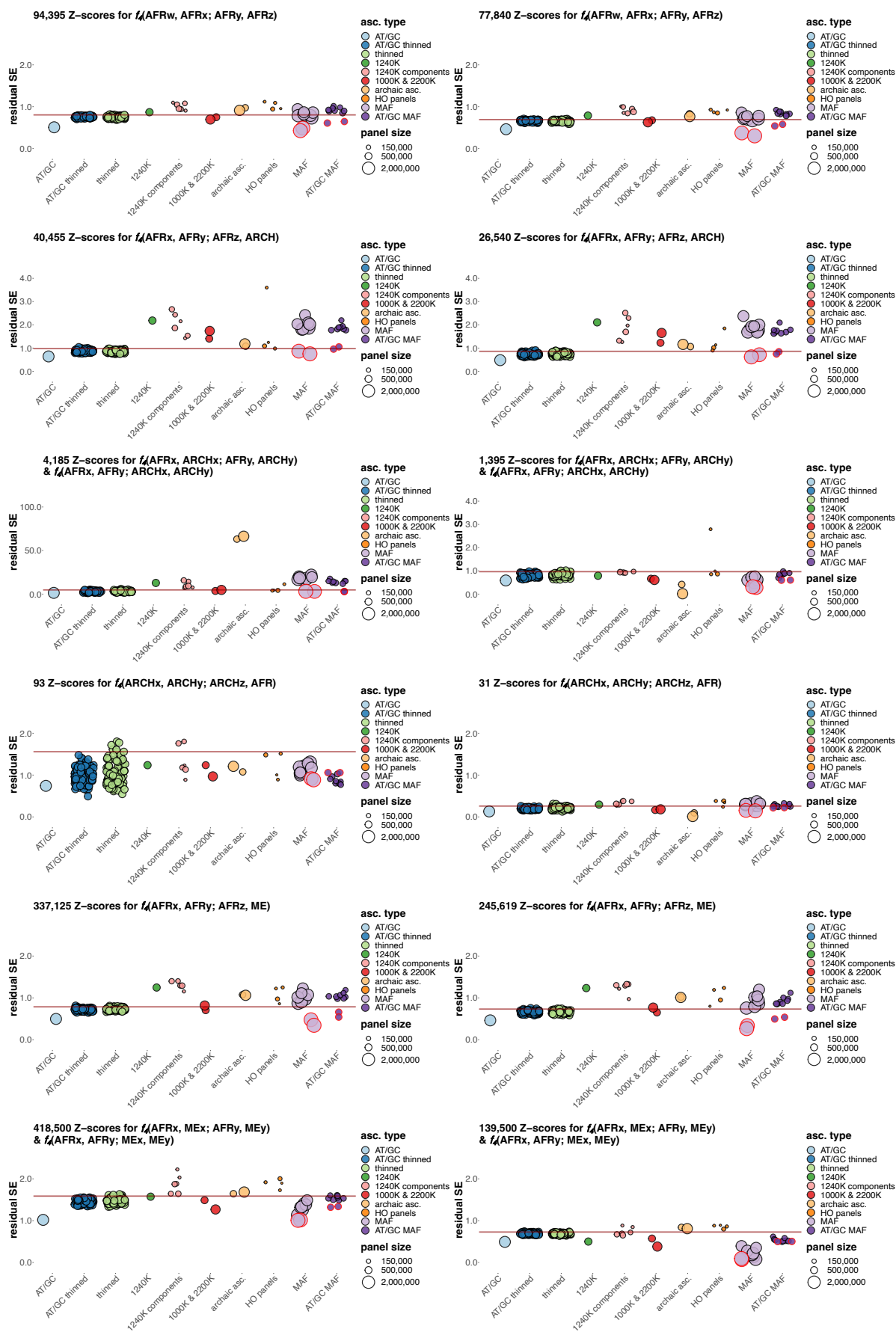

C

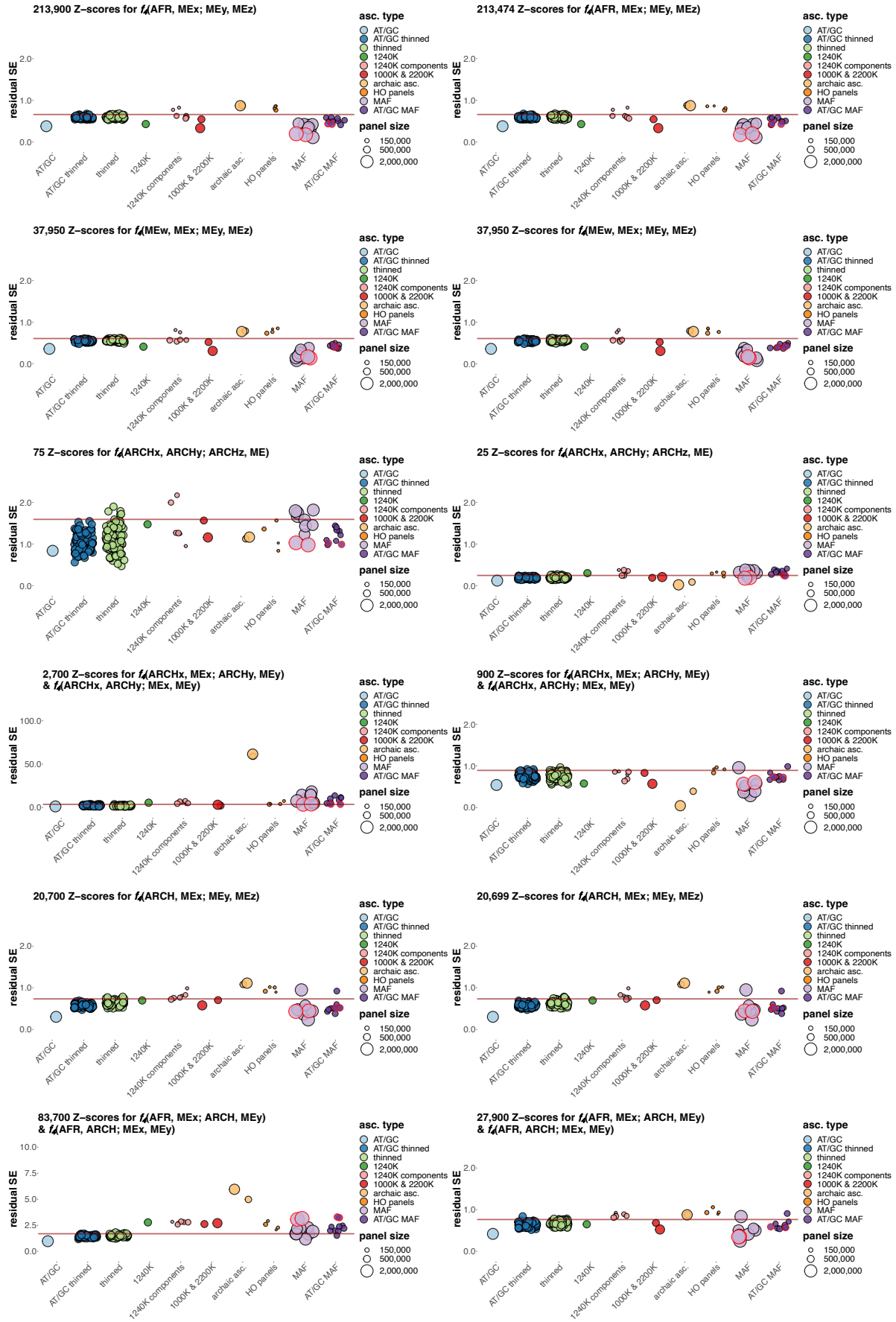

d

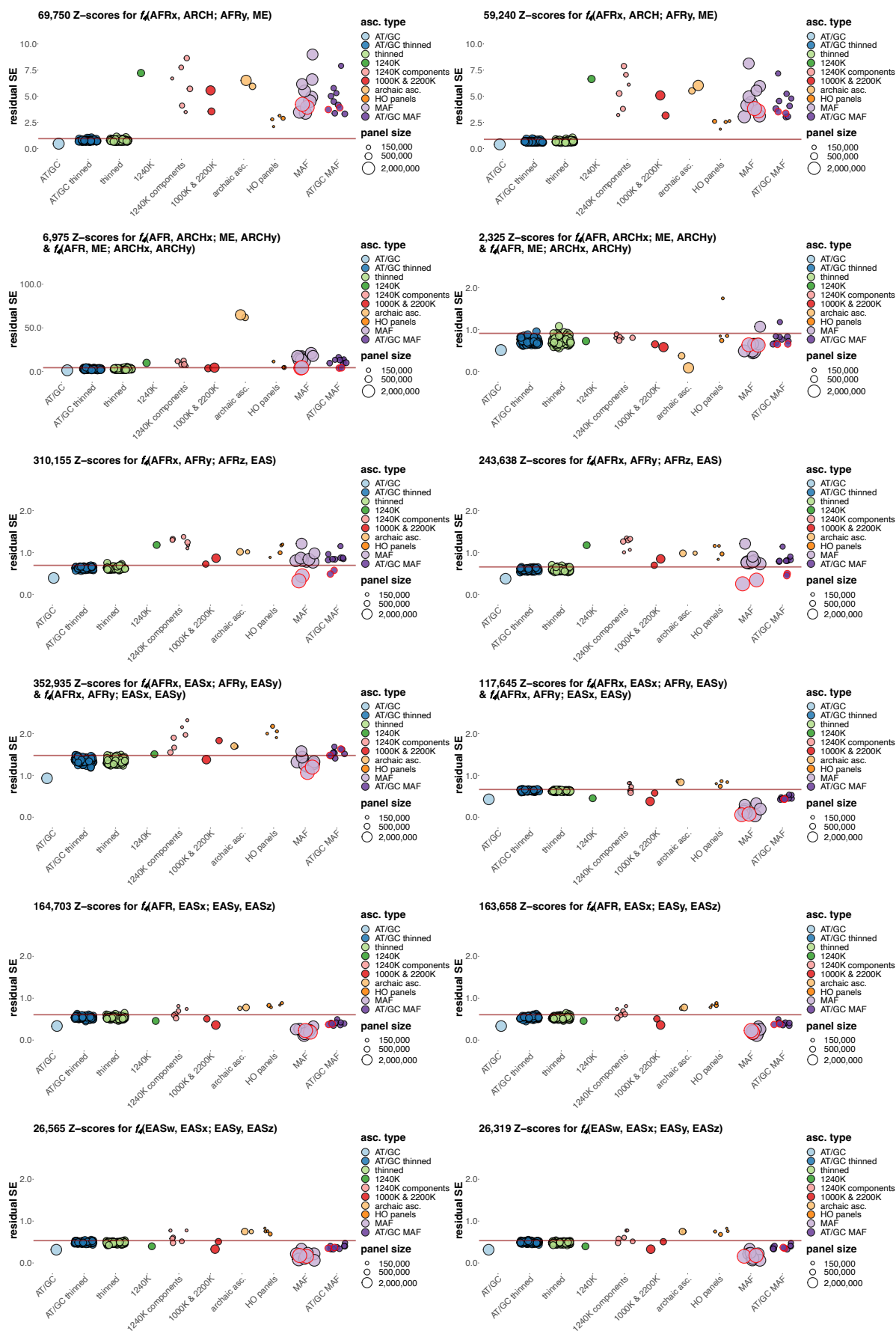

e

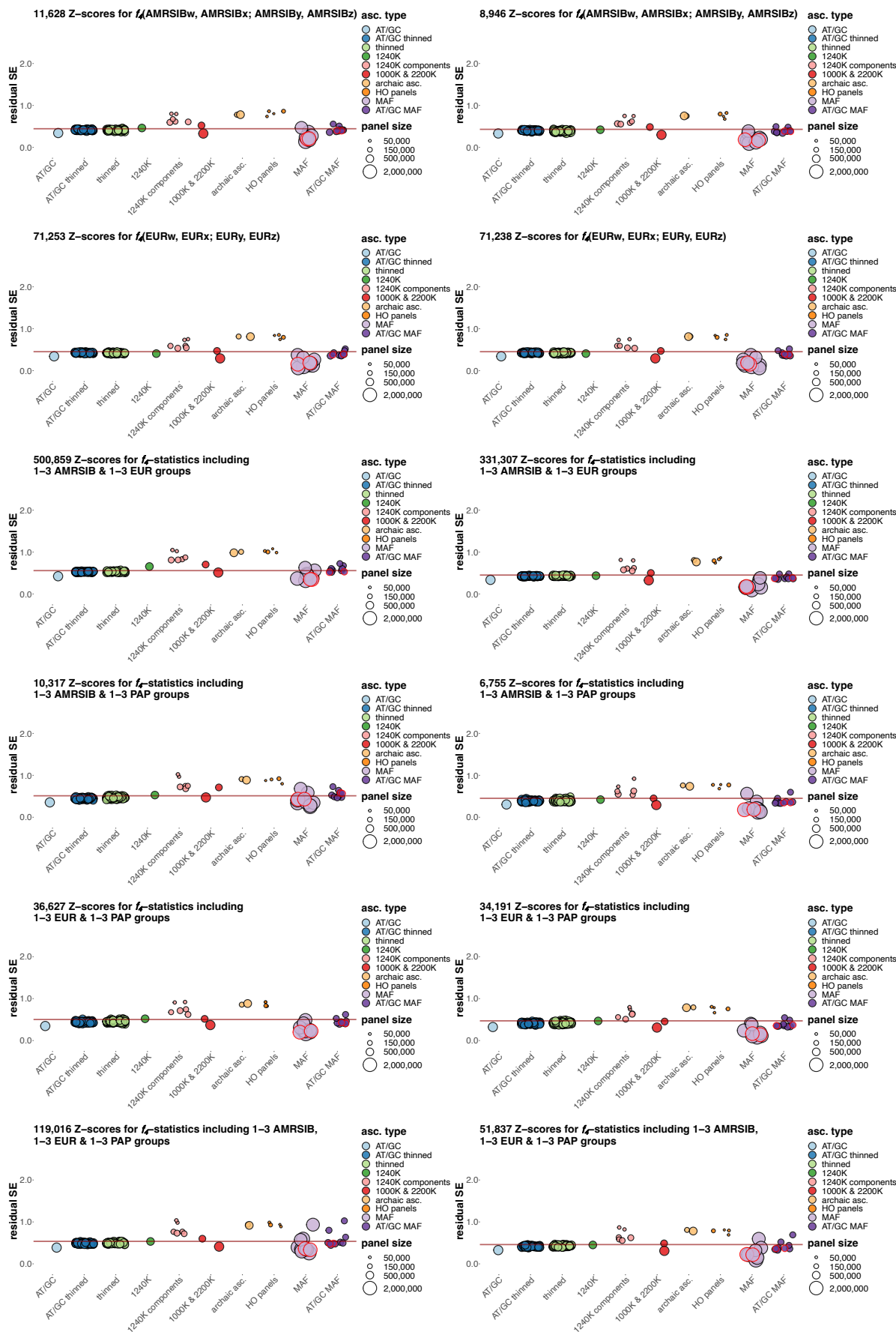

Supplement: S11 Fig — Results for ascertainment on variants common in Africans (either those having no detectable West Eurasian ancestry according to Fan et al. [67] or on all Africans in the SGDP dataset) are circled in red. Results are shown for 27 classes of f4-statistics indicated in plot titles in panels a-e. The following abbreviations are used for naming the f4-statistics classes: AFR, African populations; AMRSIB, Native American and Siberian populations; ARCH, archaic human individuals (Neanderthals and Denisovans); chimp, chimpanzee; EAS, East Asian populations; EUR, European populations; ME, Mediterranean and Middle Eastern populations; PAP, Papuan and Australian populations. Residual SE values for f4-statistic Z-scores lying not far from 0 (absolute Z-scores on all sites < 15) are plotted in the right-hand panels, and residual SE values for all Z-scores are plotted in the left-hand panels. The 97.5% percentiles of all the thinned replicates combined, including those on all sites and AT/GC sites, are marked by the brown lines. Most y-axis scales are the same (from 0 to 2.5 SE), except for few cases in panels a, b, c, and d. Size of the resulting SNP panels is coded by point size, and ten broad ascertainment types are coded by color according to the legends. Thirty eight site subsampling schemes were explored (see a list in the legend for Fig 2). (PDF) [file pgen.1010931.s011.pdf]

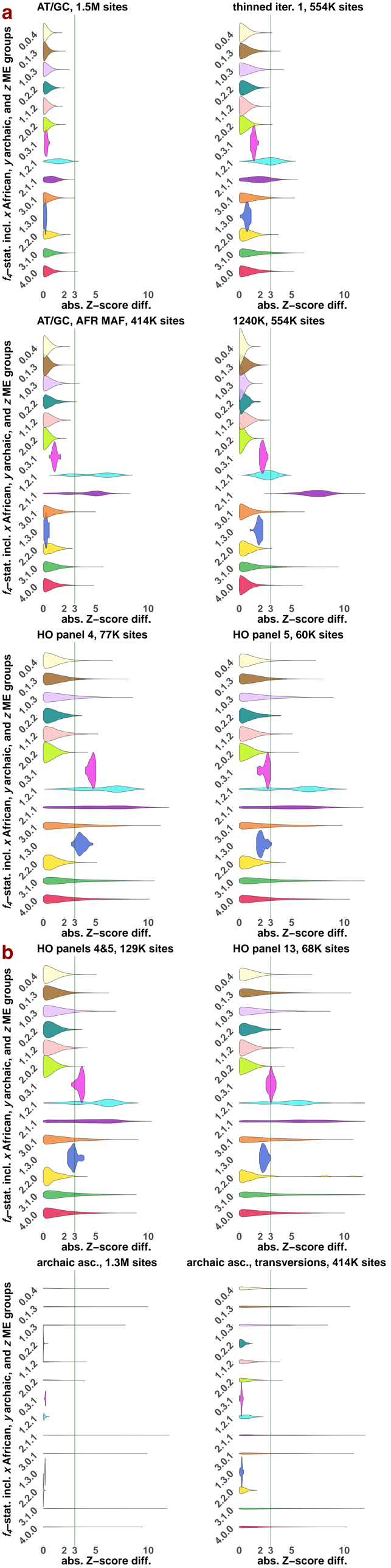

Supplement: S12 Fig — For brevity, f4-statistic classes are labelled on y-axes as x,y,z. For instance, “2.1.1” stands for all possible f4-statistics including two African, one archaic, and one Mediterranean/Middle Eastern groups. Z-score difference was calculated as the Z-score on all sites (ca. 10 million sites) minus the Z-score on an ascertained dataset, and the distributions of absolute difference values are visualized. Ascertainment types and site counts are shown in plot titles. Only statistics with absolute Z-scores below 15 on all sites were considered for this analysis. (PDF) [file pgen.1010931.s012.pdf]

**a**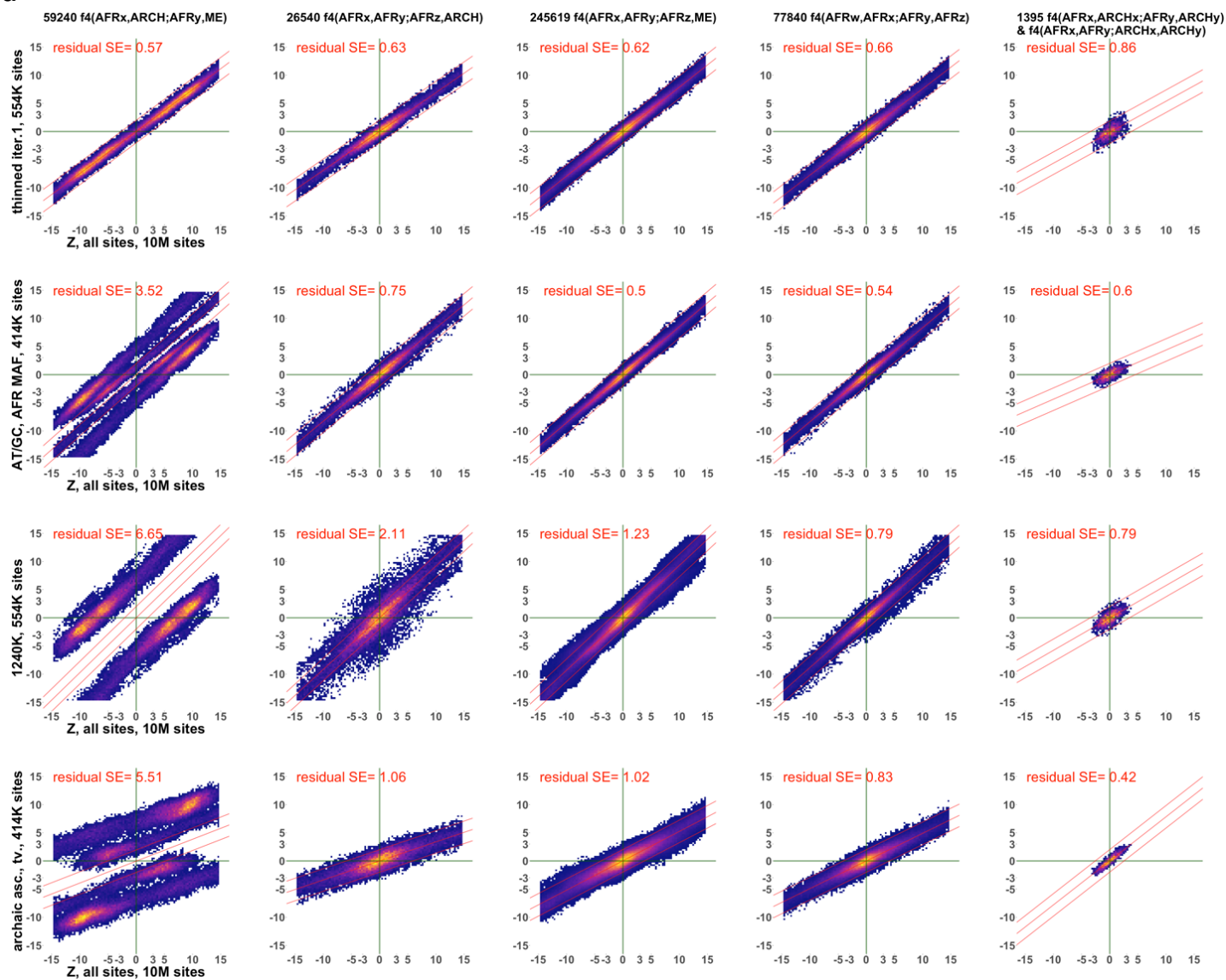

**b**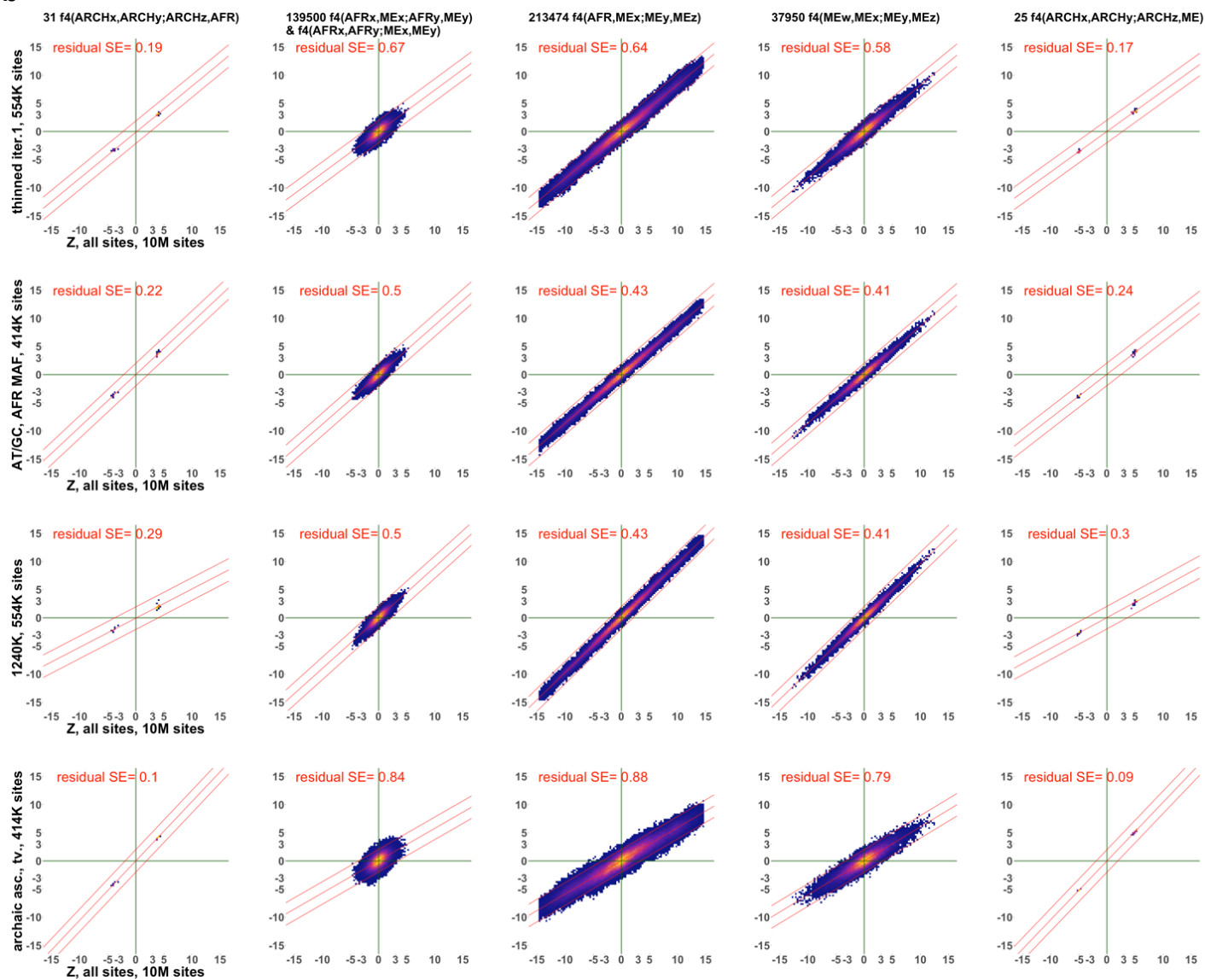

**C**

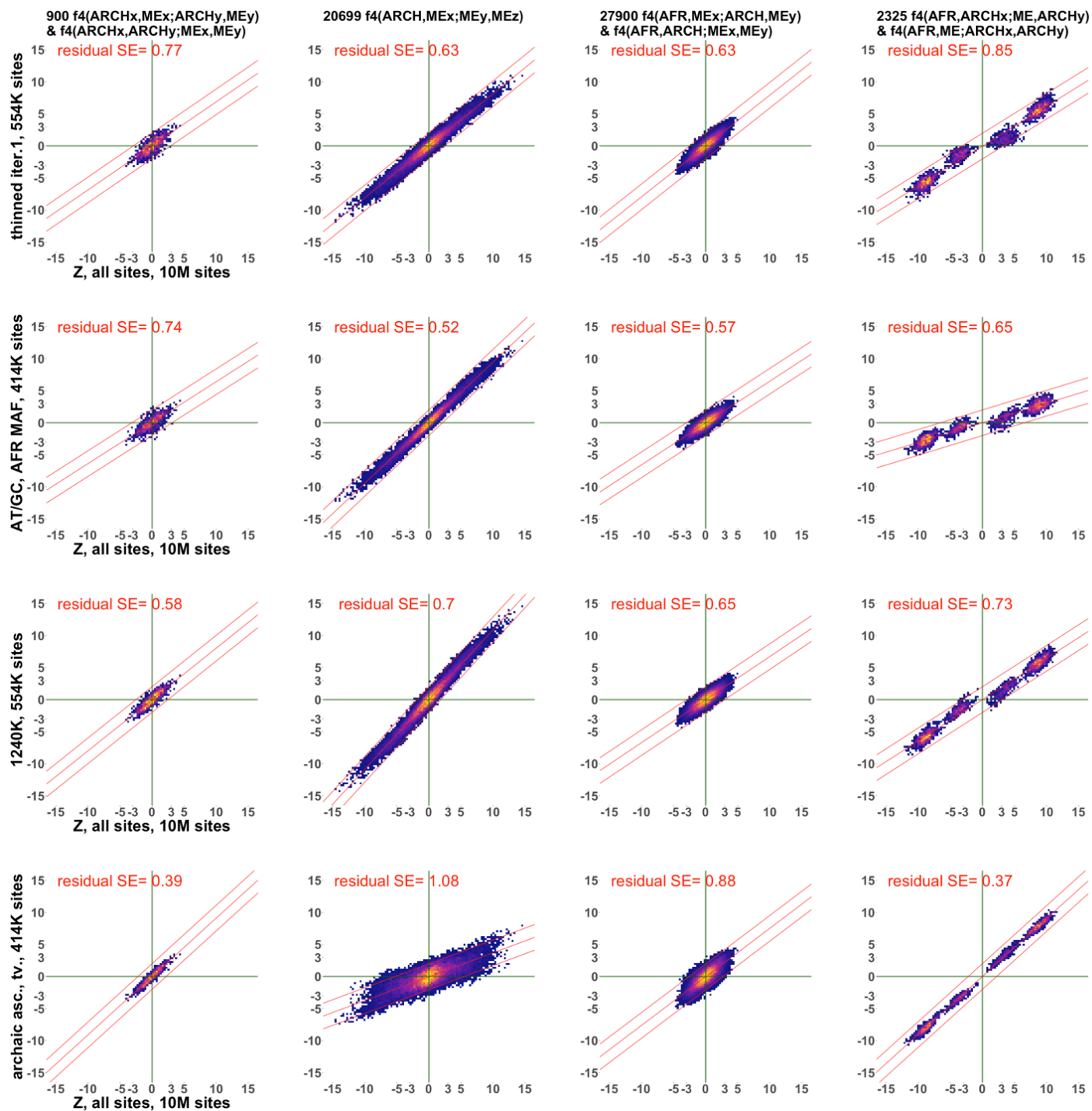

Supplement: S13 Fig — The class labels and numbers of statistics plotted are shown in the top row of each panel (a, b, and c). Instead of individual points, heatmaps illustrating point density are shown. Z-scores on all sites (ca. 10 million sites, as indicated on the x-axes) are compared to Z-scores on ascertained datasets on the y-axes. Ascertainment types and site counts are shown on the y-axes. All plots are based only on statistics with absolute Z-scores below 15 on all sites. A linear trend fitted to the data and lines representing ± 2 SE are shown in red. Residual SE values of those linear trends are shown in each plot in red. (PDF) [file pgen.1010931.s013.pdf]

94,297 stat.  $f_4(\text{AFRx}, \text{ARCH}; \text{AFRy}, \text{non-AFR})$

1240K

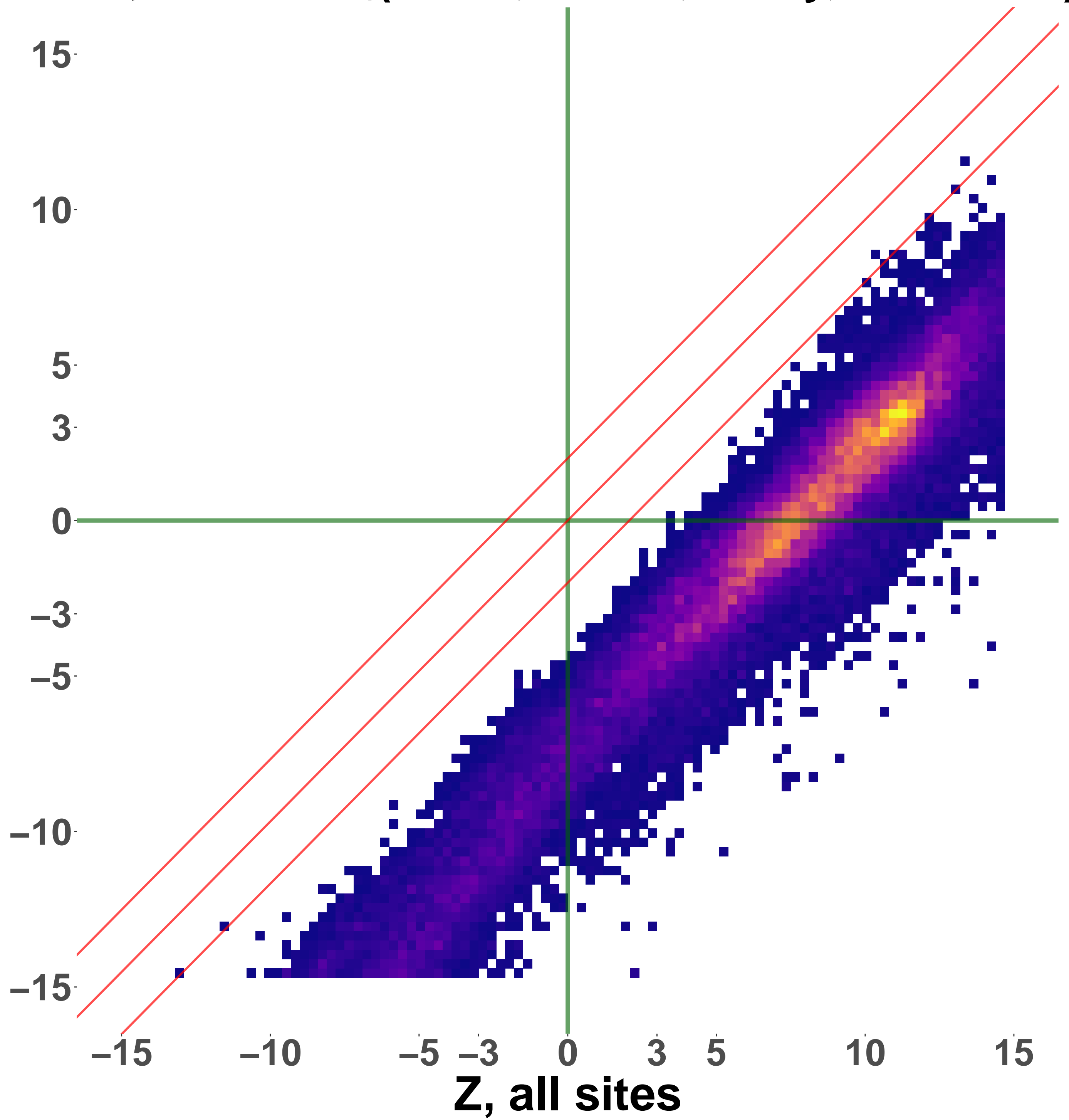

Supplement: S14 Fig — The following abbreviations are used: AFR, African populations; ARCH, archaic human individuals (Neanderthals and Denisovans); non-AFR, non-African populations. Instead of individual points, a heatmap illustrating point density is shown. Z-scores on all sites are compared to Z-scores on the 1240K dataset on the y-axis (site counts varied across population quadruplets since no missing data at the group level was allowed in each quadruplet). The plot includes only statistics with absolute Z-scores below 15 on all sites. A linear trend fitted to the data and lines representing ± 2 SE are shown in red. The intercept of the linear trend was set at 0. (PDF) [file pgen.1010931.s014.pdf]

**a**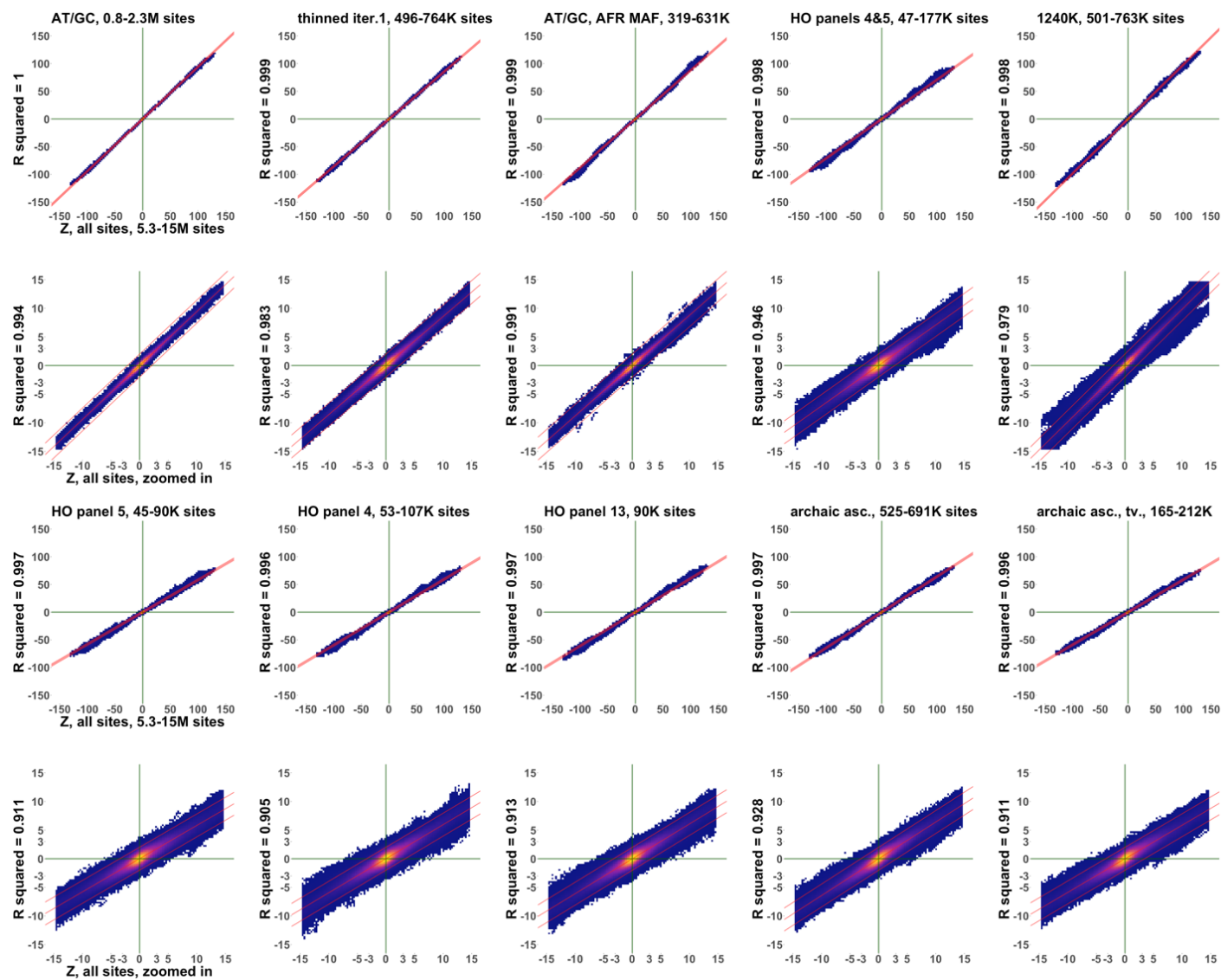

**b**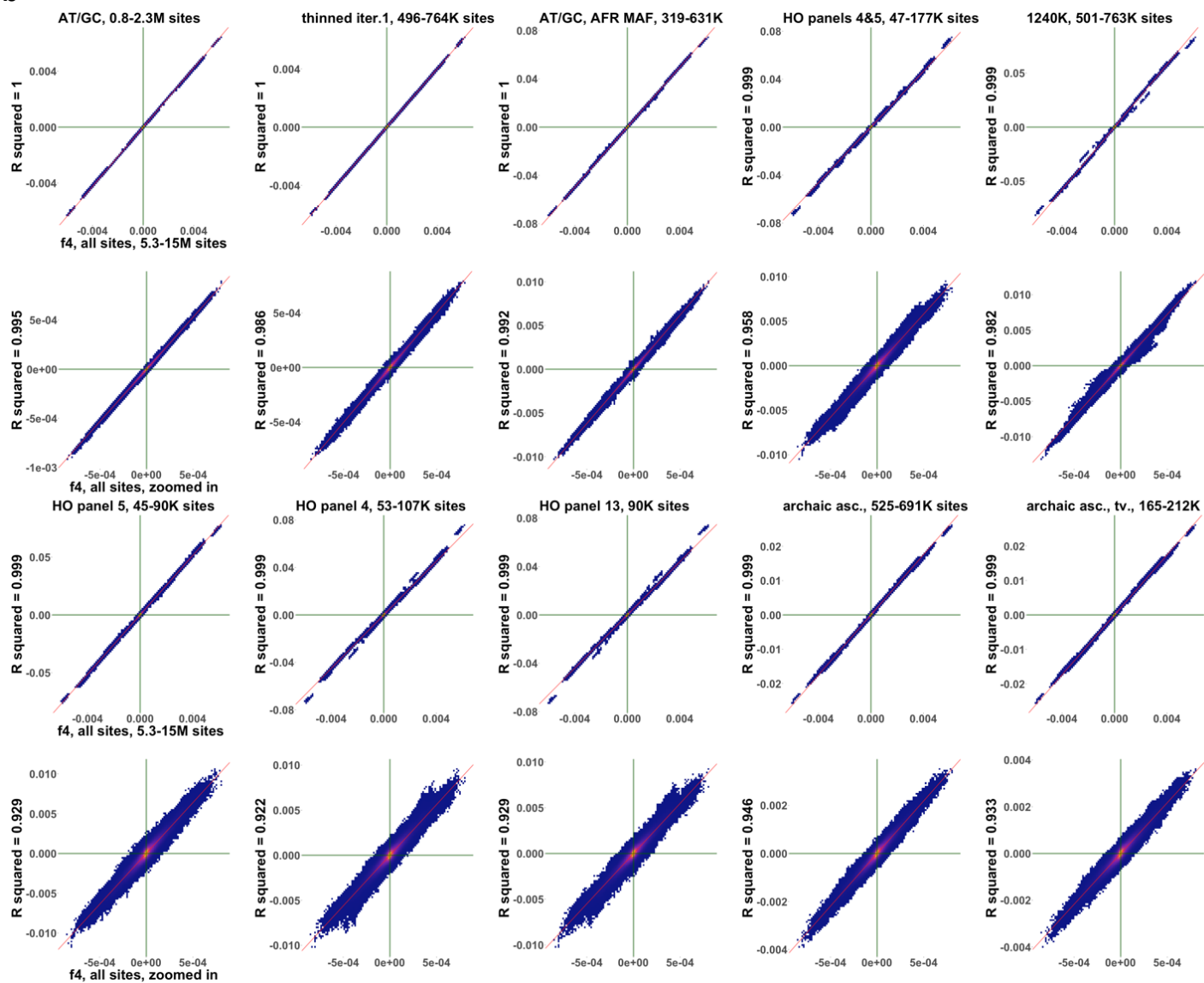

Supplement: S15 Fig — Scatterplots illustrating the effects of 10 ascertainment schemes on Z-scores (a) or f4-statistics (b) of two classes: 854,358 distinct statistics including up to three African groups and/or up to four East Asian groups, pooled with 749,700 distinct statistics including American/Siberian and/or European and/or Papuan groups. Instead of individual points, heatmaps illustrating point density are shown. Z-scores on all sites (from ca. 5.3 to 15 million sites, as indicated on the x-axes) are compared to Z-scores on ascertained datasets on the y-axes. Ascertainment schemes and site counts are shown in plot titles. The 2nd and 4th rows of plots in each panel represent close-up views on the origin of the plots: they are based on statistics with absolute Z-scores below 15 on all sites. Linear trends fitted to the data and lines representing ± 2 SE are shown in red. Squared Pearson correlation coefficients (R2) for these data are shown on the y-axes. First, linear trends were fitted to the sets of Z-scores or f4-statistics lying near the origin (having absolute Z-score below 15 on all sites), and then the same linear equations were re-fitted to complete sets of Z-scores or f4-statistics. Since we show both f4-statistics and Z-scores side by side here, R2 was used instead of residual SE as a measure of correlation. (PDF) [file pgen.1010931.s015.pdf]

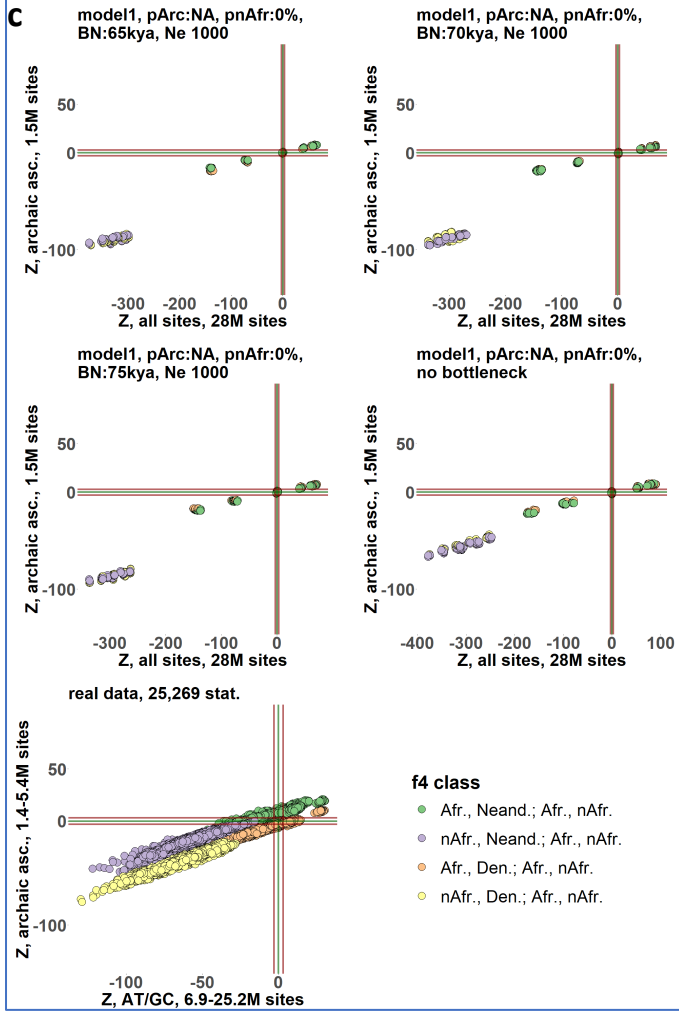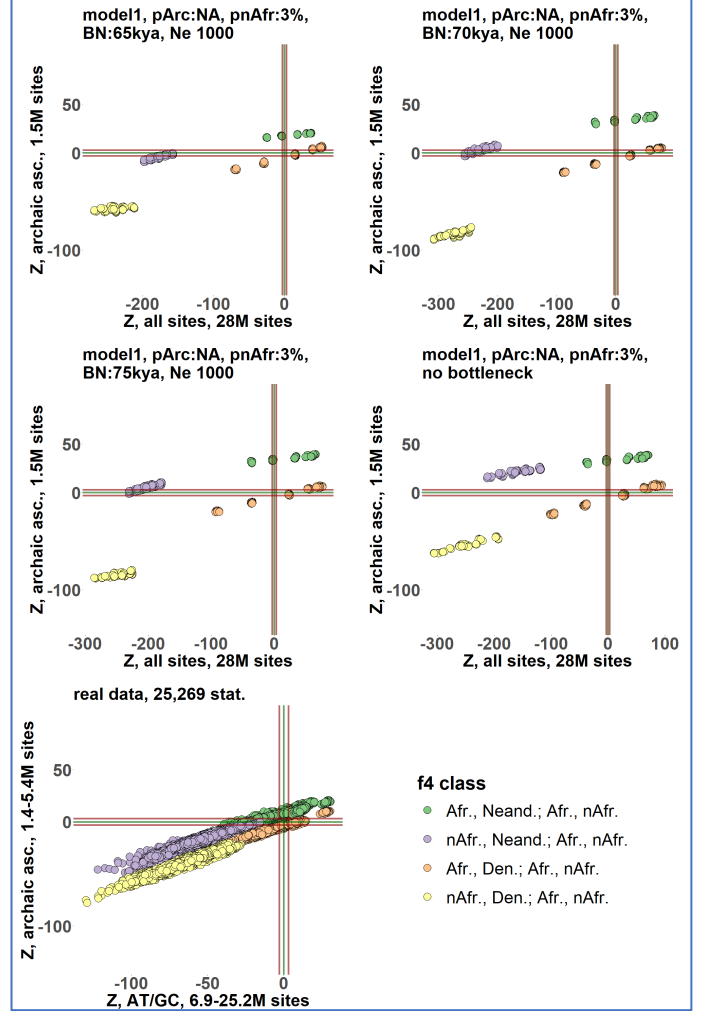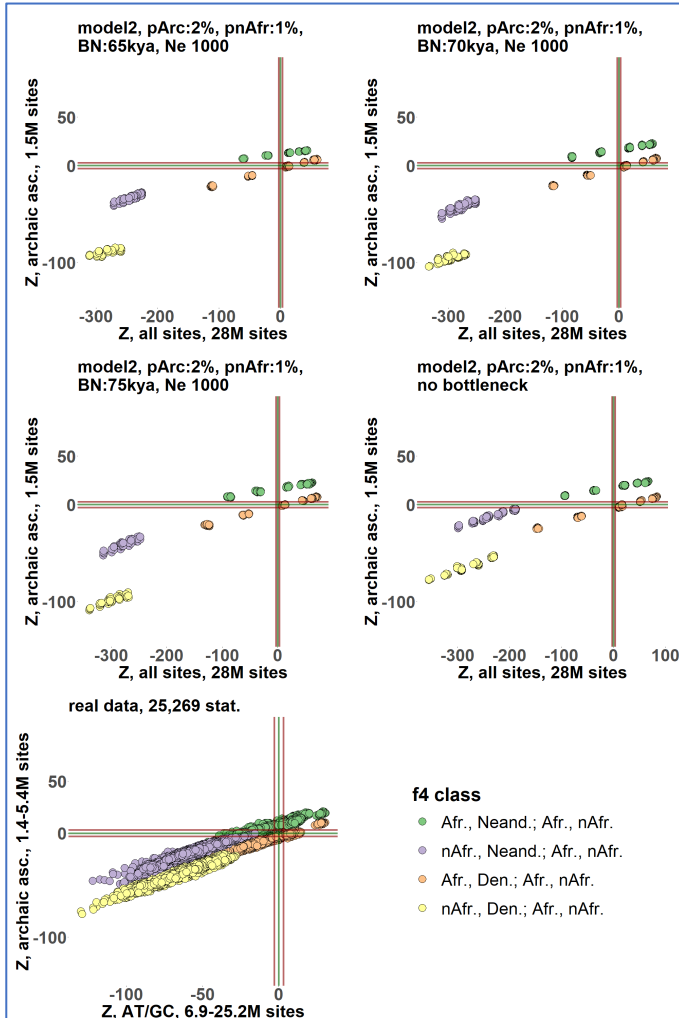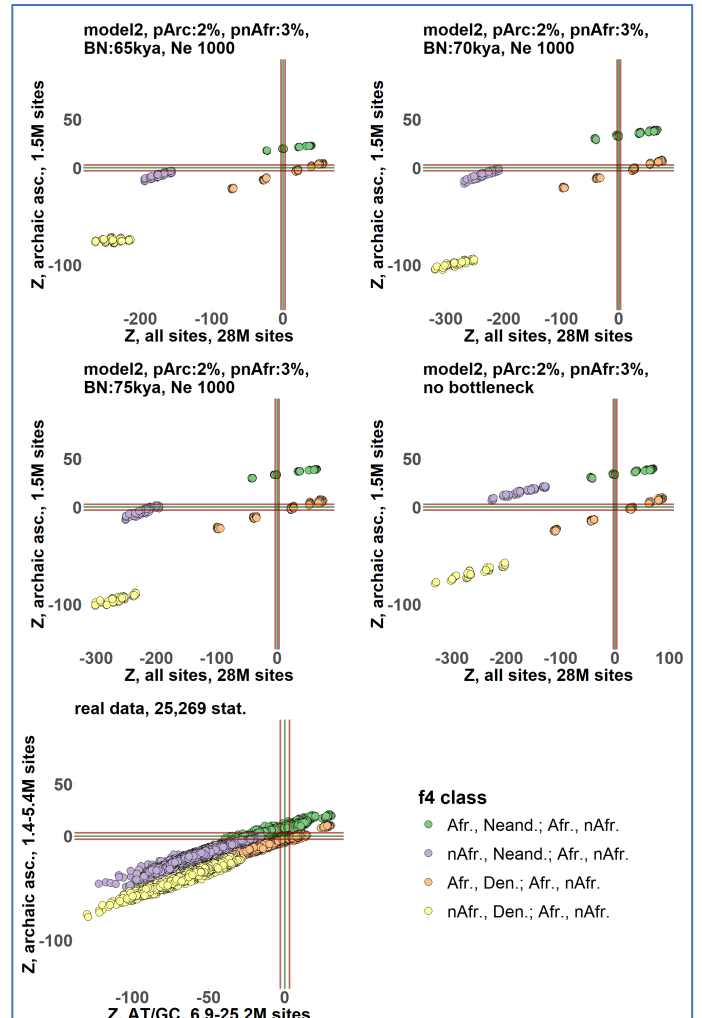

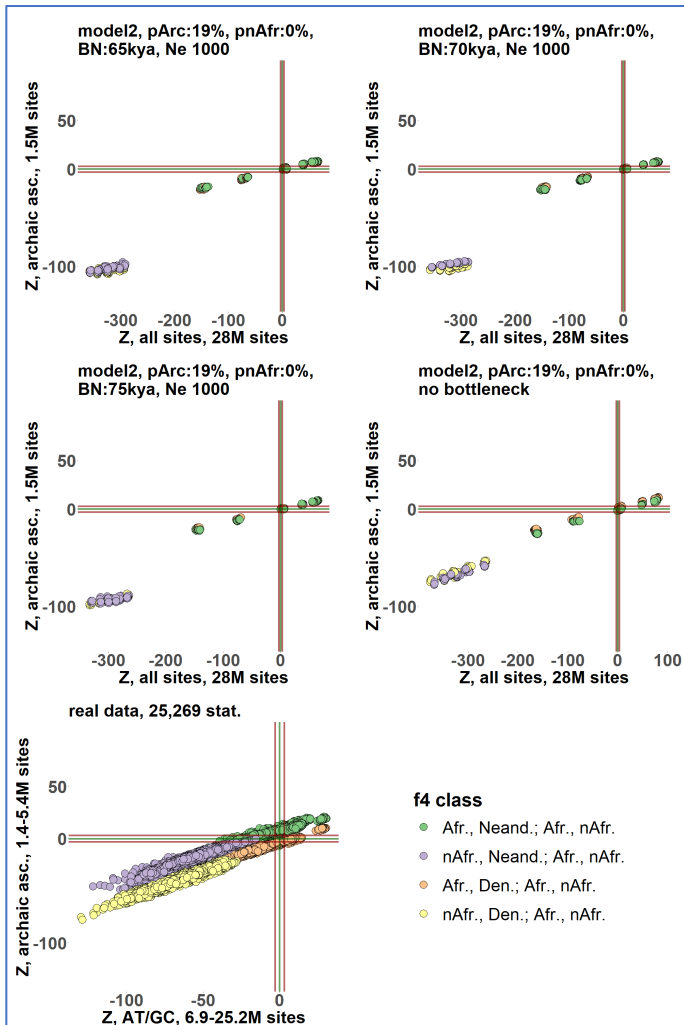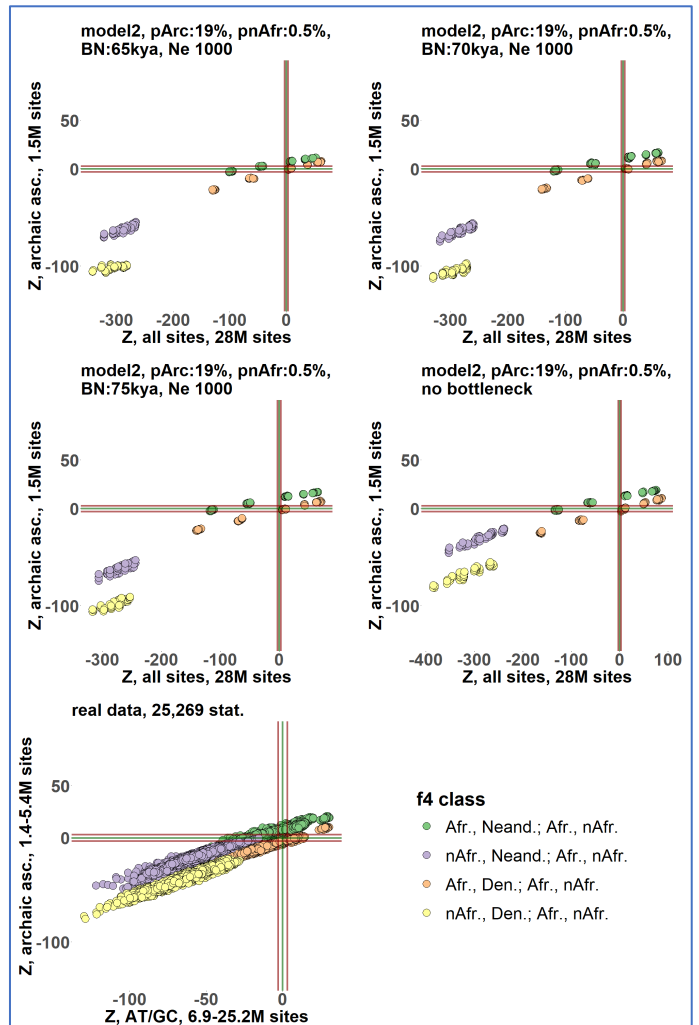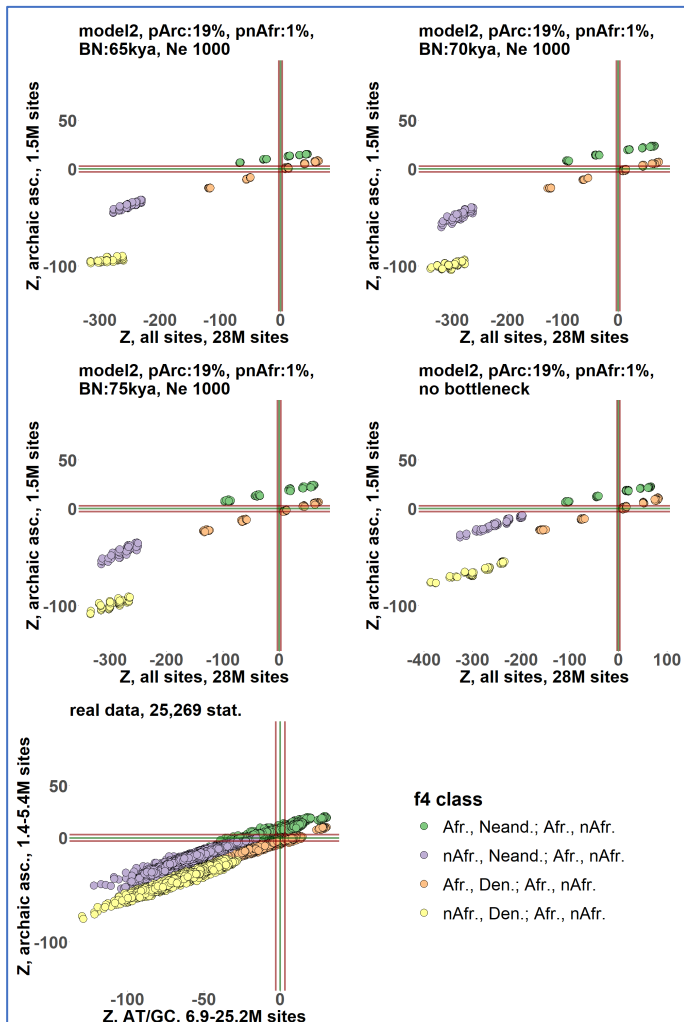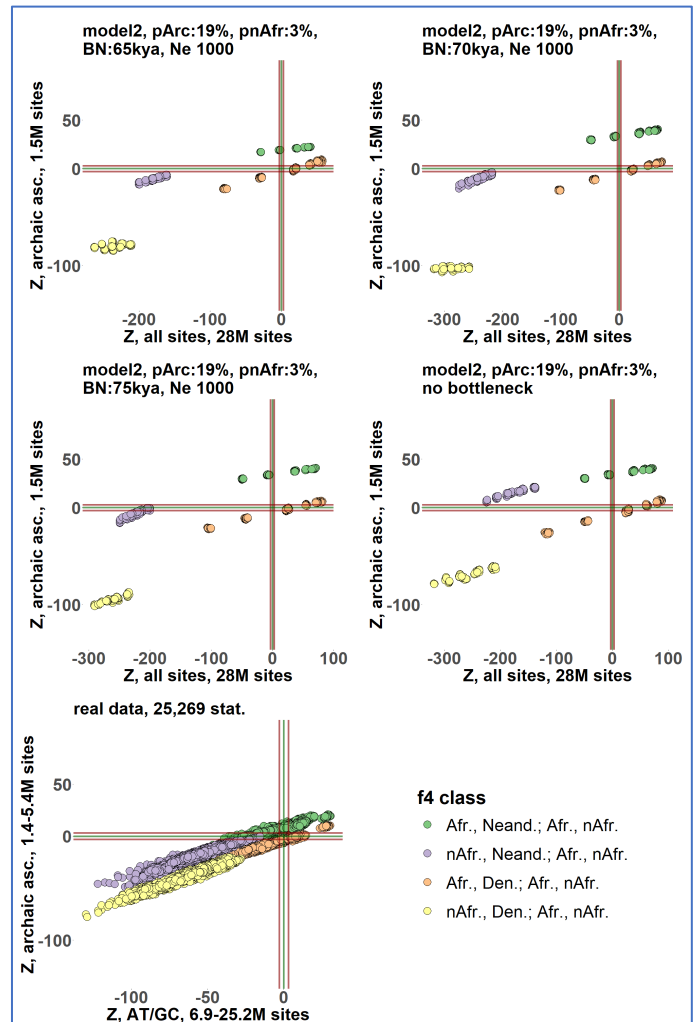

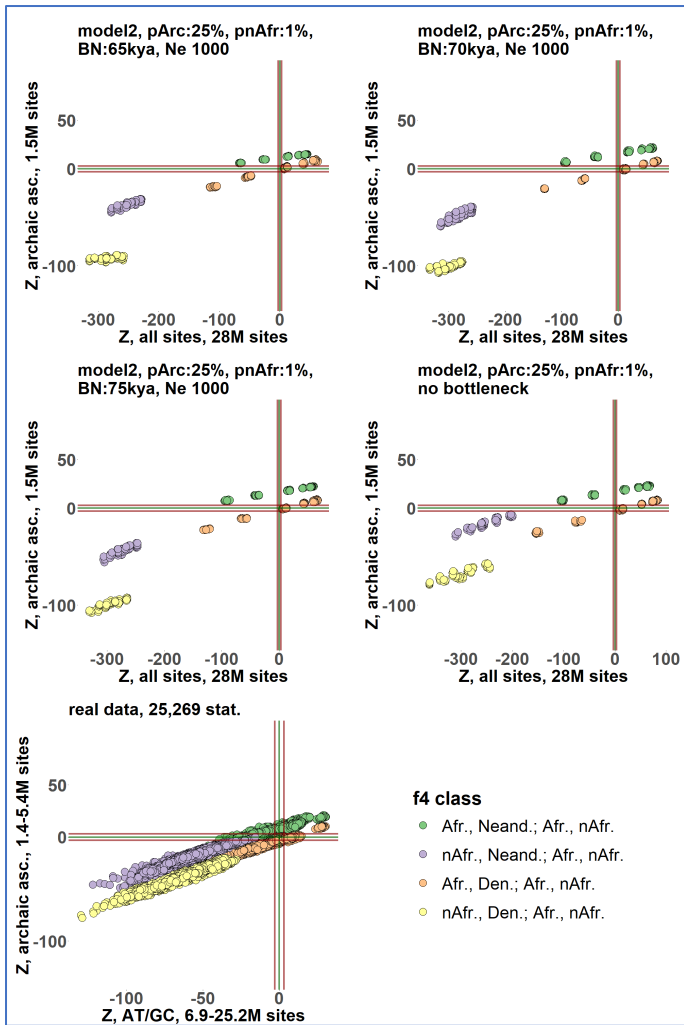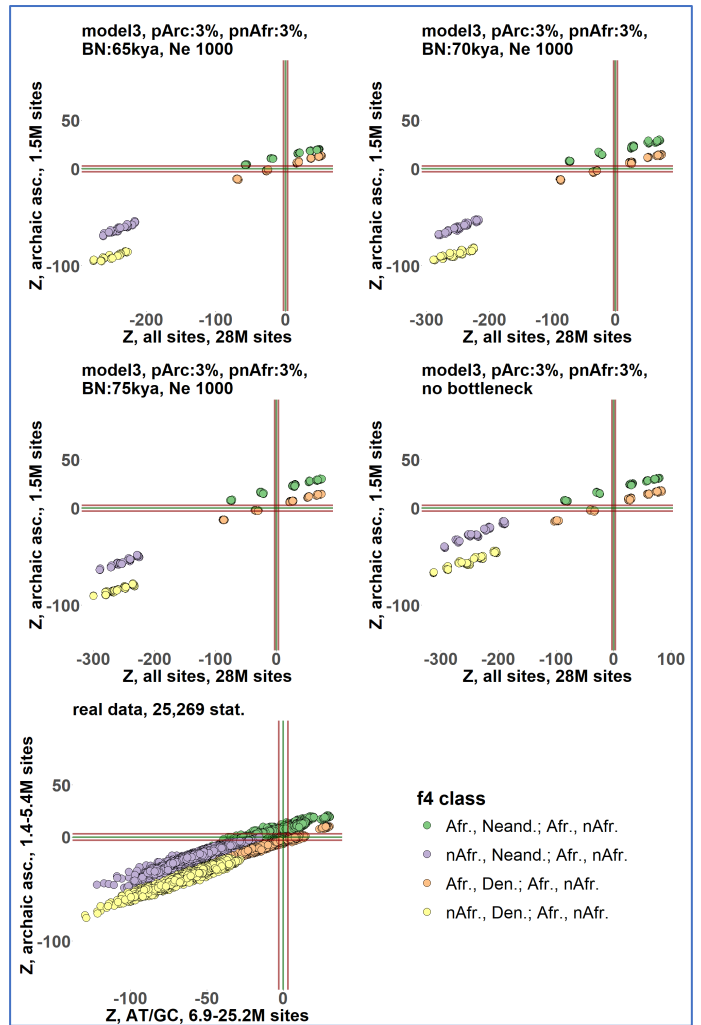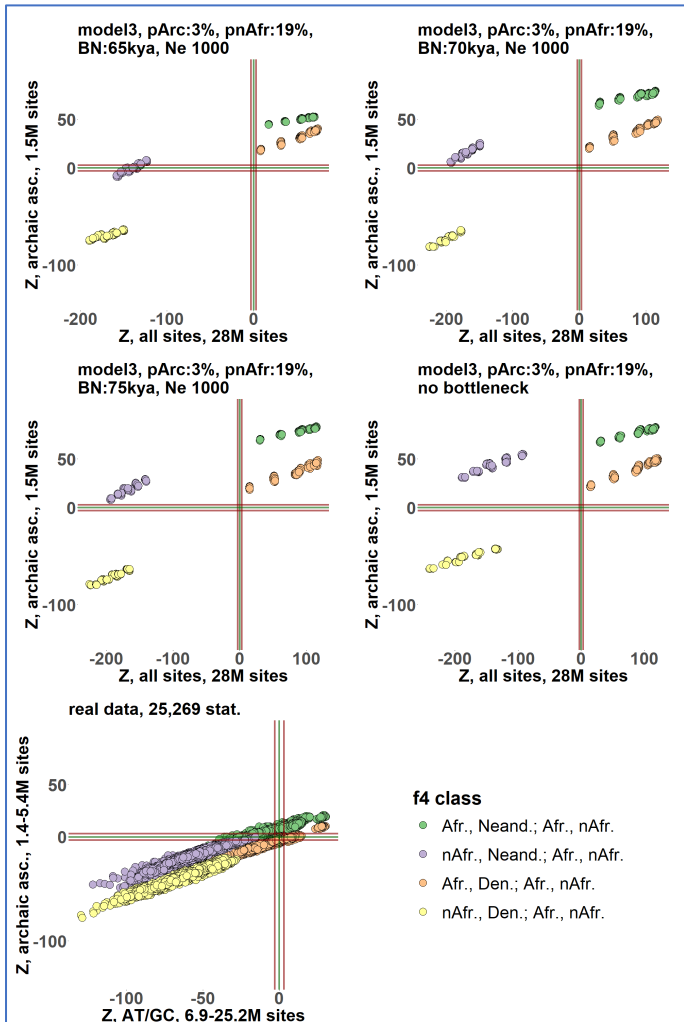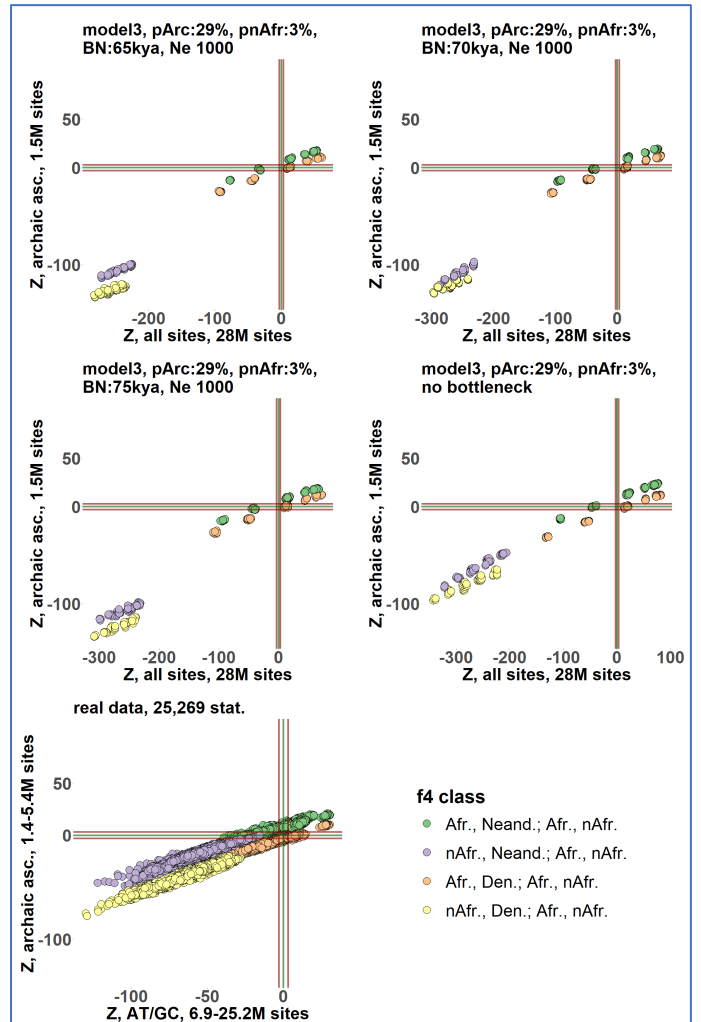

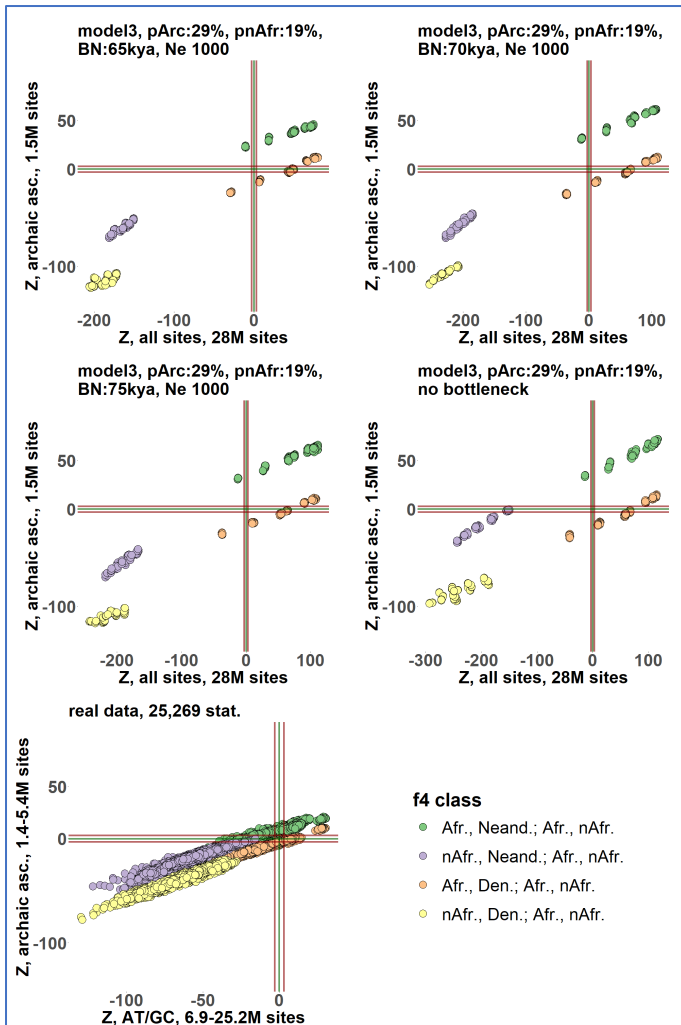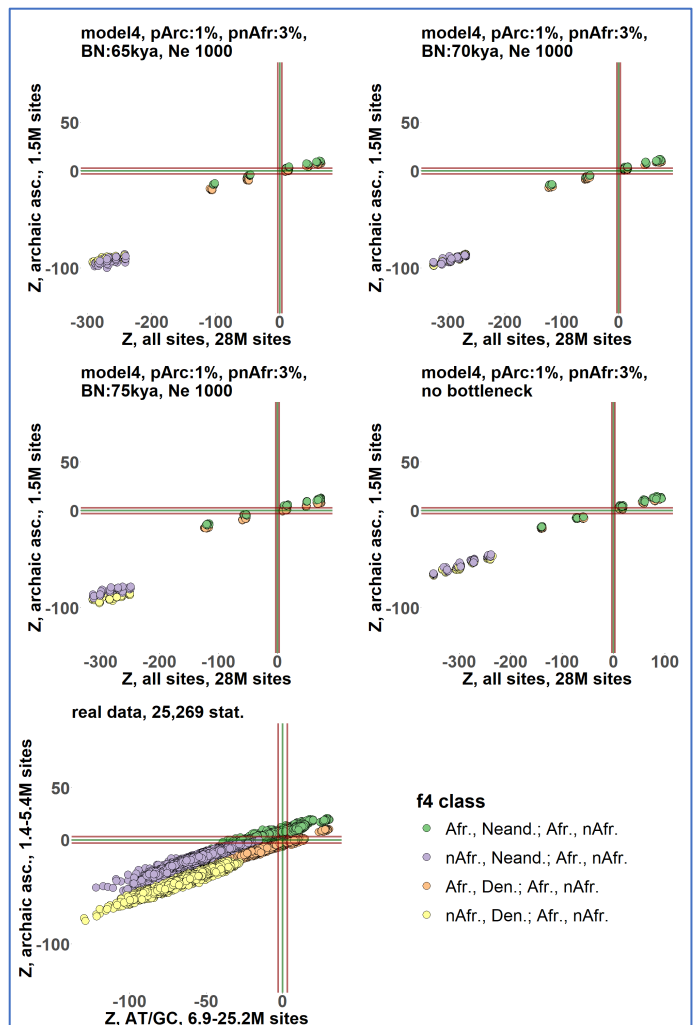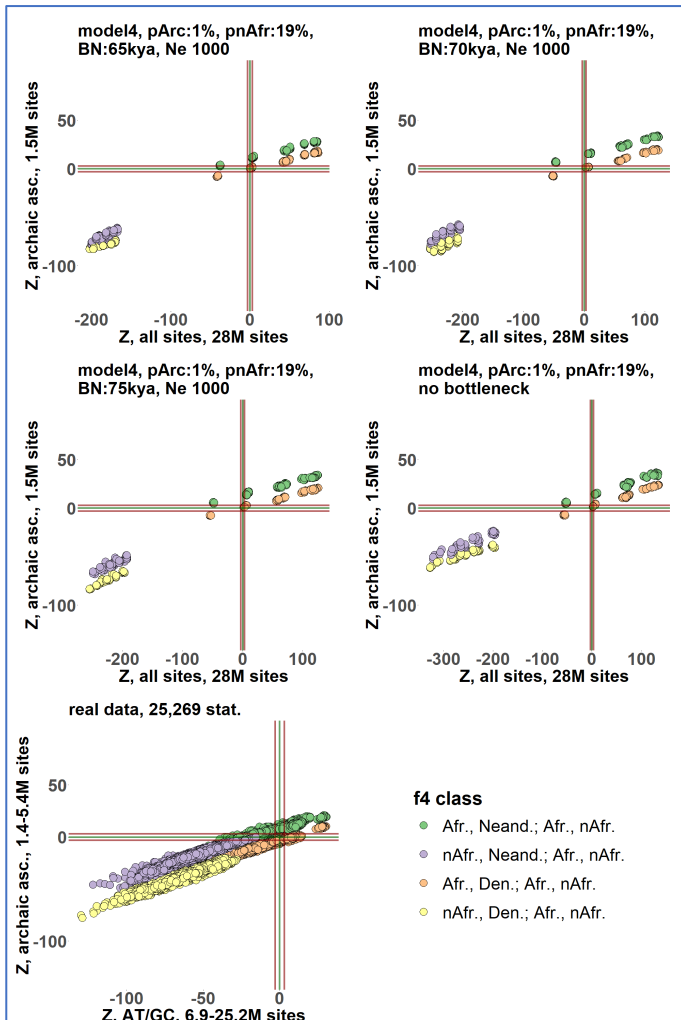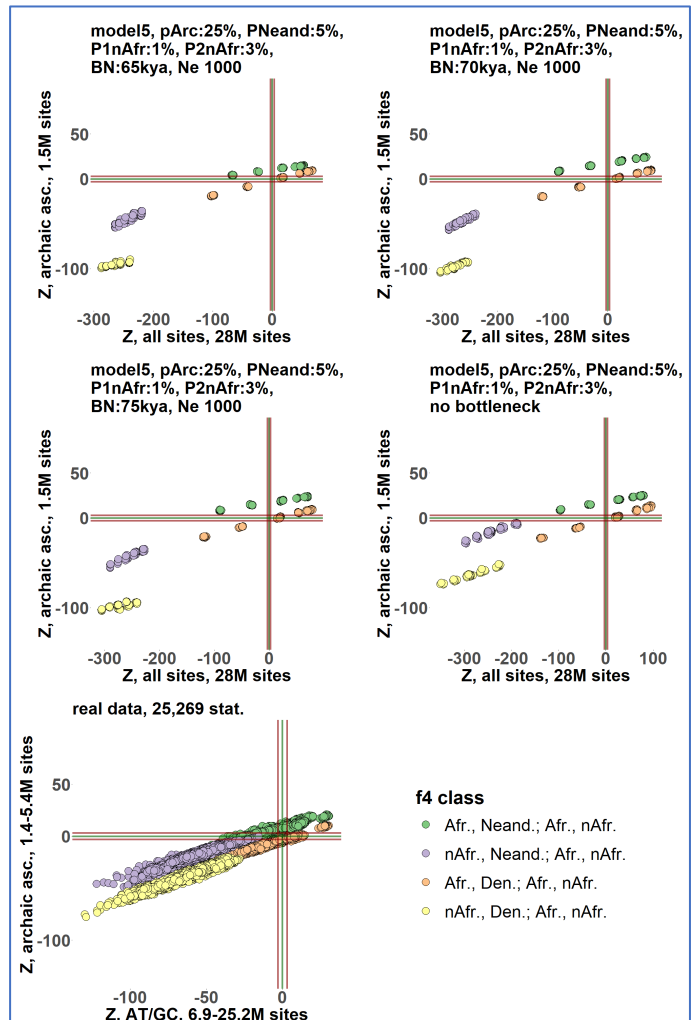

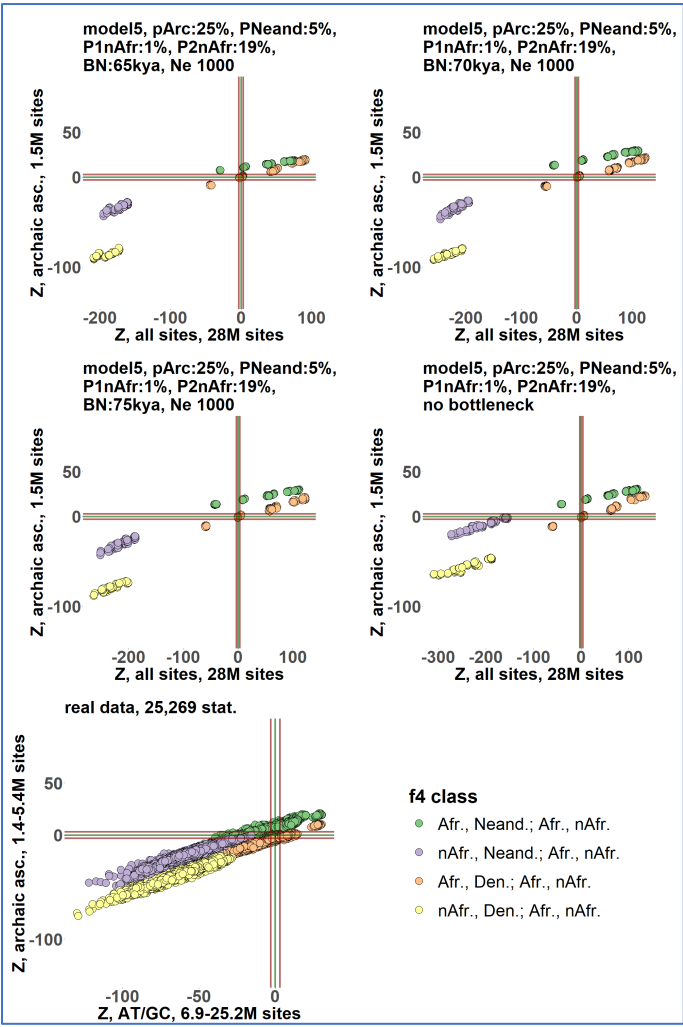

Supplement: S16 Fig — The effects of ascertaining SNPs polymorphic in archaic humans on real (a) and simulated data (b, c). We focused on two f4-statistic classes that are most strongly affected by any type of ascertainment on real data: f4(Africanx, Neanderthal or Denisovan; Africany, non-African) and f4(non-Africanx, Neanderthal or Denisovan; African, non-Africany). On real data, statistics from these classes were sampled randomly and were calculated on AT/GC sites and on archaic-ascertained sites (transitions and transversions), using all sites without missing data at the level of each quadruplet (i.e., using the “allsnps = TRUE” or “useallsnps: YES” setting). Papuans and Australians were excluded from the pool of AMH groups due to their Denisovan ancestry component, which was not simulated; and Africans with substantial non-African ancestry (S1 Table) were also removed to make the distinction between various classes of statistics clearer. Archaic ascertainment was performed either on a group composed of the Altai Neanderthal and Denisovan (panel a, left), or Vindija Neanderthal and Denisovan (panel a, right). A slightly different protocol was used for archaic ascertainment in other parts of this paper since it was performed on a group composed of both Neanderthal individuals and the Denisovan. Graphs illustrating five classes of simulated demographic histories are shown in panel b and scatterplots illustrating the effects of ascertaining SNPs polymorphic in the group composed of one “Neanderthal” and one “Denisovan” individual on genetic data simulated according to those histories are shown in panel c. The same “Neanderthal” and “Denisovan” individuals were used for ascertainment and for calculating f4-statistics, which is a non-optimal (Fig 4A) but inevitable approach in practice. On the graphs (b), the following abbreviations are used: Afr., Africans; nAfr., non-Africans; Den., Denisovan; Neand., Neanderthal. Alternative positions of the out-of-Africa bottleneck simulated at 65, 70 [file pgen.1010931.s016.pdf]
