# Supplementary material for: Modeling of African population history using f-statistics is biased when applying all previously proposed SNP ascertainment schemes
Source: PLoS Genet. 2023 Sep 7;19(9):e1010931. doi: 10.1371/journal.pgen.1010931 (PMC10508636; doi:10.1371/journal.pgen.1010931)
Supplement: S4 Fig — The search for optimal topologies was performed on three datasets: 1240K, AT/GC mutation types, and all sites. (a) The published 10-population model [9] with 8 admixture events is plotted, with parameter estimates obtained on the three datasets, and with corresponding worst f4-statistic residuals (WR) and log-likelihood scores (LL) shown above the graphs. Distinct populations are colored along with their ancestral lineages; for instance, the cluster of West African populations is colored in brown. (b) Density plots illustrating fits of ca. 10,000 distinct topologies per dataset (found with findGraphs [23]) in the LL vs. WR coordinates. Green vertical lines mark median LL of the highest-ranking newly found model fitted to bootstrap replicates of the dataset, and red lines mark 95th percentile of that distribution. Position of the published 10-population model in these coordinates is marked by the cyan dot. (c) Highest-ranking models (according to LL) with 8 admixture events found with findGraphs on each dataset. Populations and ancestral lineages are color-coded in the same way as in panel a. WR and LL of these models are shown above the plots. We also compared the fit (i.e., LL) of the highest-ranking newly found model with that of the published model on each dataset relying on a bootstrap resampling approach: comparison of two LL distributions on 500 resampled sets of SNP blocks [23]. For all three SNP sets, the difference in LL between the highest-ranking model found by the automated search and the published model was statistically significant, with empirical two-tailed p-values ranging from <0.002 to 0.032. In other words, it was shown for the three datasets explored that the published model fits significantly worse than the newly found highest-ranking models (however, this does not prove that the newly found models approximate the true population history better, see an analysis in Fig 1 from Maier et al. [23]). (PDF) [file pgen.1010931.s004.pdf]

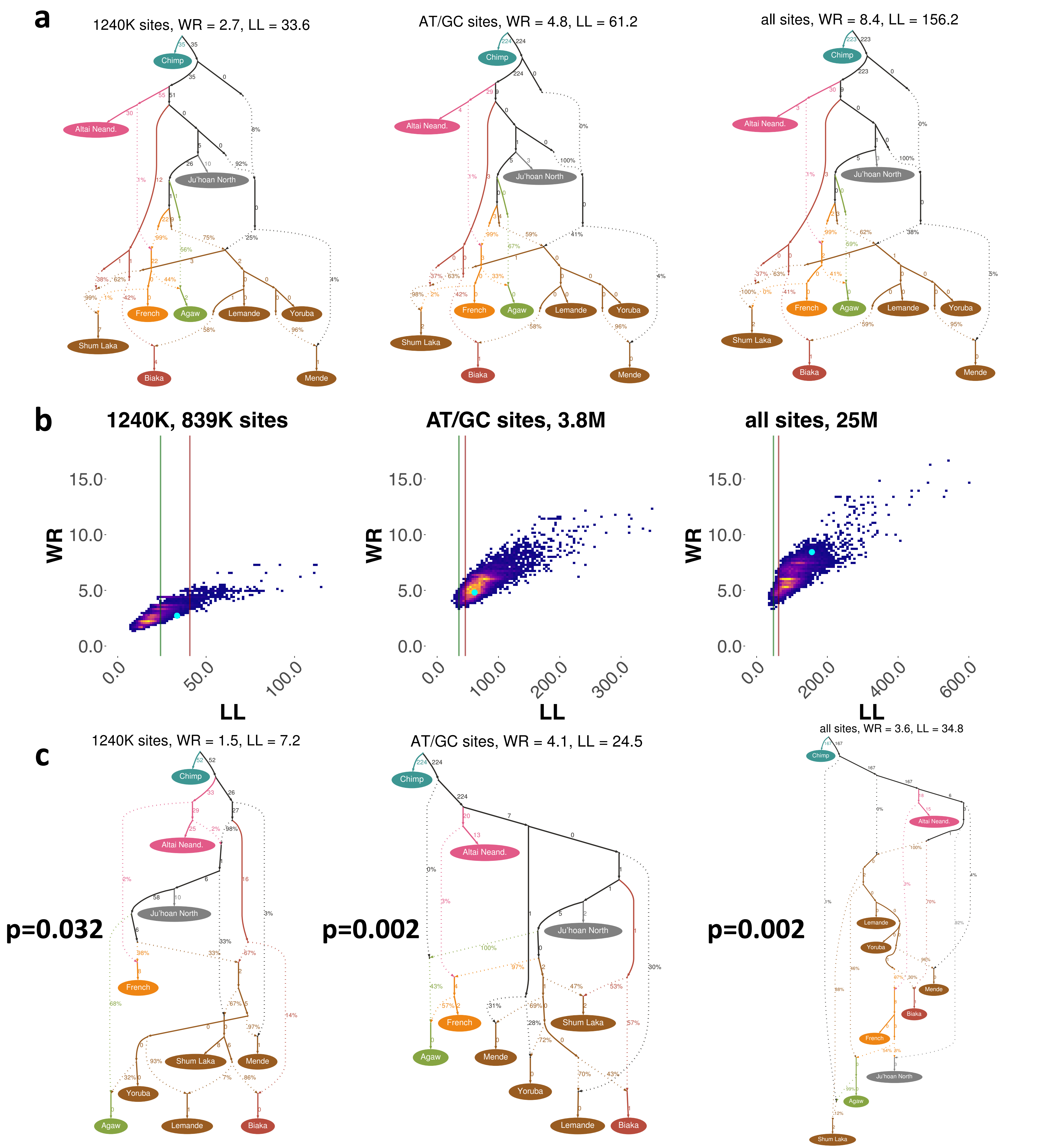
a
1240K sites, WR = 2.7, LL = 33.6
AT/GC sites, WR = 4.8, LL = 61.2
all sites, WR = 8.4, LL = 156.2
Chimp
Altai Neand.
Ju'hoan North
French
Agaw
Lemande
Yoruba
Shum Laka
Biaka
Mende
b
1240K, 839K sites
AT/GC sites, 3.8M
all sites, 25M
WR
LL
15.0
10.0
5.0
0.0
50.0
100.0
200.0
300.0
400.0
600.0
c
1240K sites, WR = 1.5, LL = 7.2
AT/GC sites, WR = 4.1, LL = 24.5
all sites, WR = 3.6, LL = 34.8
p=0.032
p=0.002
p=0.002
