# Supplementary material for: Modeling of African population history using f-statistics is biased when applying all previously proposed SNP ascertainment schemes
Source: PLoS Genet. 2023 Sep 7;19(9):e1010931. doi: 10.1371/journal.pgen.1010931 (PMC10508636; doi:10.1371/journal.pgen.1010931)
Supplement: S17 Fig — The effects of ascertaining SNPs polymorphic in archaic humans on real (a) and simulated data (b, c). We focused on 15 f4-statistic classes that are most strongly affected by archaic ascertainment on simulated data (see a list of classes in the legend for panel a). On real data, statistics from these classes were sampled exhaustively and were calculated on AT/GC sites and on archaic-ascertained sites (transitions and transversions), using all sites without missing data at the level of each quadruplet (i.e., using the “allsnps = TRUE” or “useallsnps: YES” setting). Papuans and Australians were excluded from the pool of AMH groups due to their Denisovan ancestry component, which was not simulated; and Africans with substantial non-African ancestry (S1 Table) were also removed to make the distinction between various classes of statistics clearer. Archaic ascertainment was performed either on a group composed of the Altai Neanderthal and Denisovan (panel a, left), or Vindija Neanderthal and Denisovan (panel a, right). A slightly different protocol was used for archaic ascertainment in other parts of this paper since it was performed on a group composed of both Neanderthals and the Denisovan. Graphs illustrating five classes of simulated demographic histories are shown in panel b and scatterplots illustrating the effects of ascertaining SNPs polymorphic in the group composed of one “Neanderthal” and one “Denisovan” individual on genetic data simulated according to those histories are shown in panel c. The same “Neanderthal” and “Denisovan” individuals were used for ascertainment and for calculating f4-statistics, which is a non-optimal (Fig 4A) but inevitable approach in practice. On the graphs (b), the following abbreviations are used: Afr., Africans; nAfr., non-Africans; Den., Denisovan; Neand., Neanderthal. Alternative positions of the out-of-Africa bottleneck simulated at 65, 70, or 75 kya (generation time = 25 years) are marked with red dots. Gene flows from ghost u [file pgen.1010931.s017.pdf]

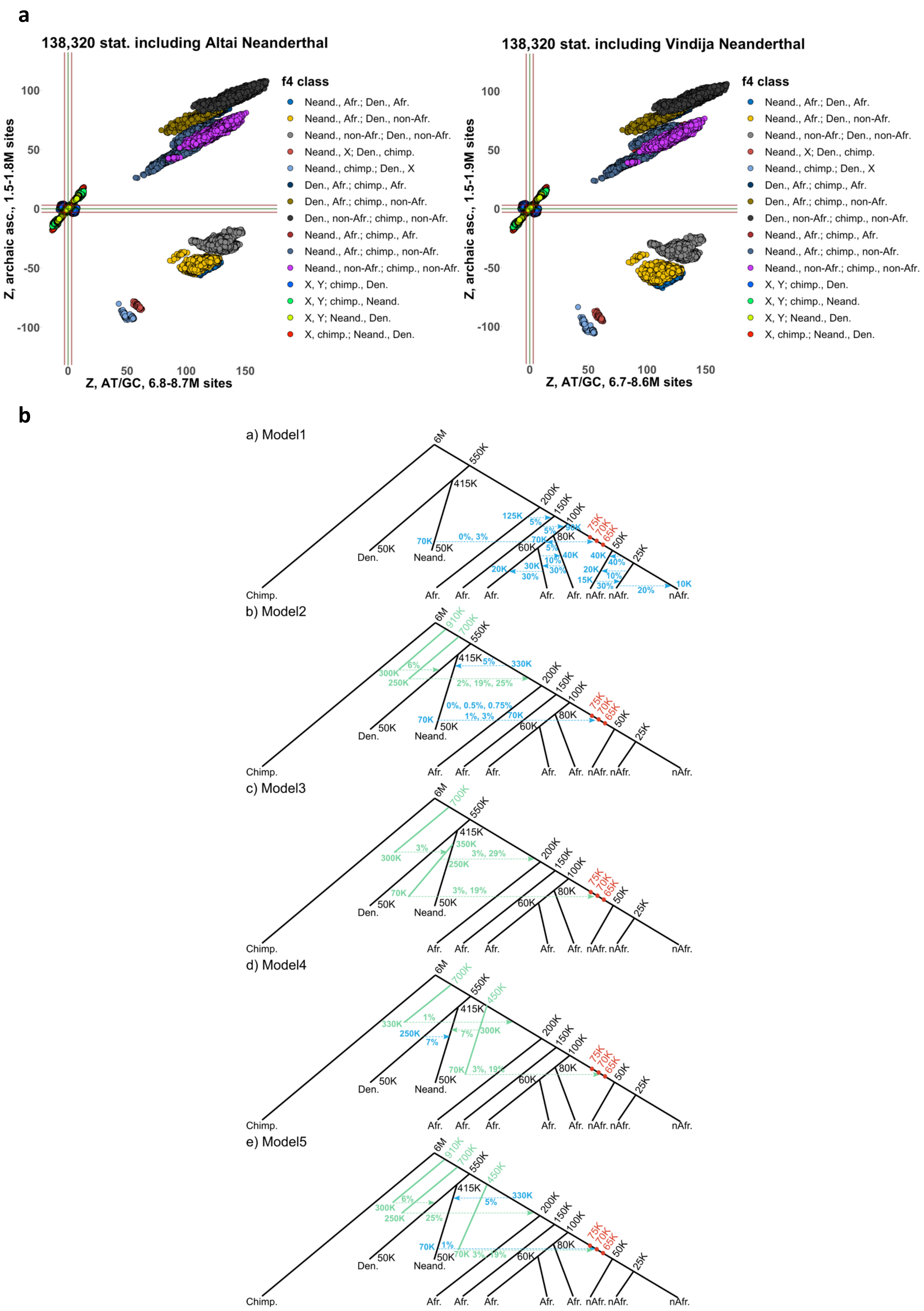
a
138,320 stat. including Altai Neanderthal
138,320 stat. including Vindija Neanderthal
Z, archaic asc., 1.5-1.8M sites
Z, archaic asc., 1.5-1.9M sites
Z, AT/GC, 6.8-8.7M sites
Z, AT/GC, 6.7-8.6M sites
f4 class
Neand., Afr.; Den., Afr.
Neand., Afr.; Den., non-Afr.
Neand., non-Afr.; Den., non-Afr.
Neand., X; Den., chimp.
Neand., chimp.; Den., X
Den., Afr.; chimp., Afr.
Den., Afr.; chimp., non-Afr.
Den., non-Afr.; chimp., non-Afr.
Neand., Afr.; chimp., Afr.
Neand., Afr.; chimp., non-Afr.
Neand., non-Afr.; chimp., non-Afr.
X, Y; chimp., Den.
X, Y; chimp., Neand.
X, Y; Neand., Den.
X, chimp.; Neand., Den.
b
a) Model1
b) Model2
c) Model3
d) Model4
e) Model5
Chimp.
Den.
Neand.
Afr.
nAfr.
6M
550K
415K
200K
150K
100K
80K
60K
50K
25K
75K
70K
65K
910K
700K
450K
330K
300K
250K
125K
90K
40K
30K
20K
15K
10K

c

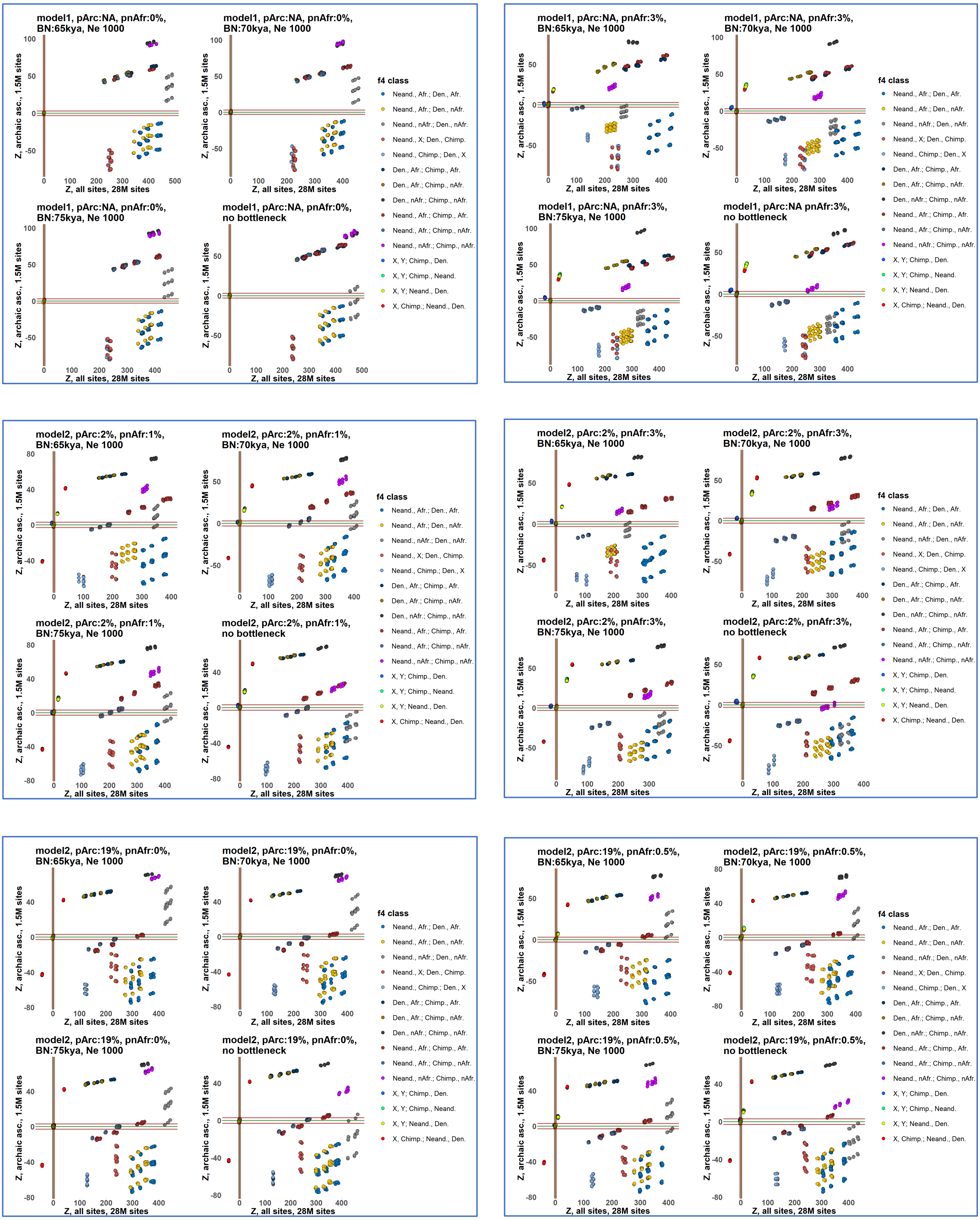

model1, pArc:NA, pnAfr:0%, BN:65kya, Ne 1000
model1, pArc:NA, pnAfr:0%, BN:70kya, Ne 1000
model1, pArc:NA, pnAfr:0%, BN:75kya, Ne 1000
model1, pArc:NA, pnAfr:0%, no bottleneck
Z, archaic asc., 1.5M sites
Z, all sites, 28M sites
f4 class
Neand., Afr.; Den., Afr.
Neand., Afr.; Den., nAfr.
Neand., nAfr.; Den., nAfr.
Neand., X; Den., Chimp.
Neand., Chimp.; Den., X
Den., Afr.; Chimp., Afr.
Den., Afr.; Chimp., nAfr.
Den., nAfr.; Chimp., nAfr.
Neand., Afr.; Chimp., Afr.
Neand., Afr.; Chimp., nAfr.
Neand., nAfr.; Chimp., nAfr.
X, Y; Chimp., Den.
X, Y; Chimp., Neand.
X, Y; Neand., Den.
X, Chimp.; Neand., Den.
model1, pArc:NA, pnAfr:3%, BN:65kya, Ne 1000
model1, pArc:NA, pnAfr:3%, BN:70kya, Ne 1000
model1, pArc:NA, pnAfr:3%, BN:75kya, Ne 1000
model1, pArc:NA pnAfr:3%, no bottleneck
model2, pArc:2%, pnAfr:1%, BN:65kya, Ne 1000
model2, pArc:2%, pnAfr:1%, BN:70kya, Ne 1000
model2, pArc:2%, pnAfr:1%, BN:75kya, Ne 1000
model2, pArc:2%, pnAfr:1%, no bottleneck
model2, pArc:2%, pnAfr:3%, BN:65kya, Ne 1000
model2, pArc:2%, pnAfr:3%, BN:70kya, Ne 1000
model2, pArc:2%, pnAfr:3%, BN:75kya, Ne 1000
model2, pArc:2%, pnAfr:3%, no bottleneck
model2, pArc:19%, pnAfr:0%, BN:65kya, Ne 1000
model2, pArc:19%, pnAfr:0%, BN:70kya, Ne 1000
model2, pArc:19%, pnAfr:0%, BN:75kya, Ne 1000
model2, pArc:19%, pnAfr:0%, no bottleneck
model2, pArc:19%, pnAfr:0.5%, BN:65kya, Ne 1000
model2, pArc:19%, pnAfr:0.5%, BN:70kya, Ne 1000
model2, pArc:19%, pnAfr:0.5%, BN:75kya, Ne 1000
model2, pArc:19%, pnAfr:0.5%, no bottleneck

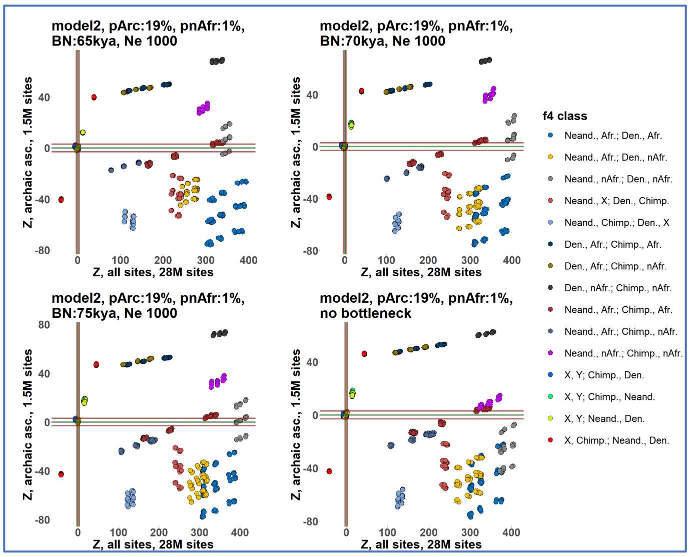
model2, pArc:19%, pnAfr:1%, BN:65kya, Ne 1000
model2, pArc:19%, pnAfr:1%, BN:70kya, Ne 1000
model2, pArc:19%, pnAfr:1%, BN:75kya, Ne 1000
model2, pArc:19%, pnAfr:1%, no bottleneck
Z, archaic asc., 1.5M sites
Z, all sites, 28M sites
f4 class
Neand., Afr.; Den., Afr.
Neand., Afr.; Den., nAfr.
Neand., nAfr.; Den., nAfr.
Neand., X; Den., Chimp.
Neand., Chimp.; Den., X
Den., Afr.; Chimp., Afr.
Den., Afr.; Chimp., nAfr.
Den., nAfr.; Chimp., nAfr.
Neand., Afr.; Chimp., Afr.
Neand., Afr.; Chimp., nAfr.
Neand., nAfr.; Chimp., nAfr.
X, Y; Chimp., Den.
X, Y; Chimp., Neand.
X, Y; Neand., Den.
X, Chimp.; Neand., Den.

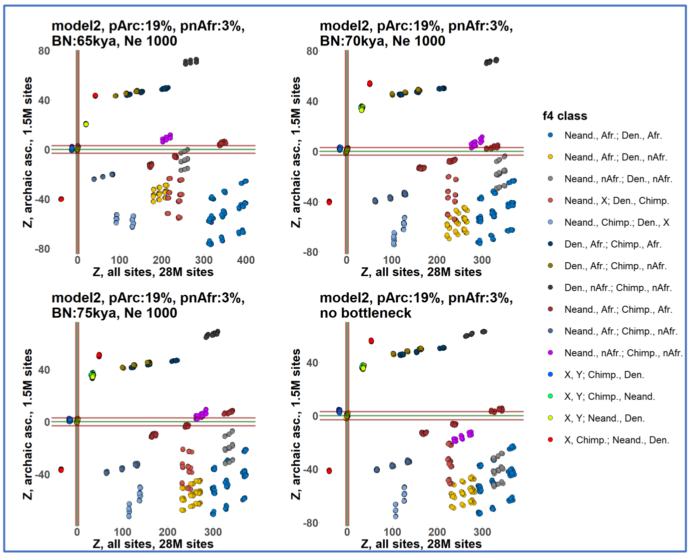
model2, pArc:19%, pnAfr:3%, BN:65kya, Ne 1000
model2, pArc:19%, pnAfr:3%, BN:70kya, Ne 1000
model2, pArc:19%, pnAfr:3%, BN:75kya, Ne 1000
model2, pArc:19%, pnAfr:3%, no bottleneck
Z, archaic asc., 1.5M sites
Z, all sites, 28M sites
f4 class
Neand., Afr.; Den., Afr.
Neand., Afr.; Den., nAfr.
Neand., nAfr.; Den., nAfr.
Neand., X; Den., Chimp.
Neand., Chimp.; Den., X
Den., Afr.; Chimp., Afr.
Den., Afr.; Chimp., nAfr.
Den., nAfr.; Chimp., nAfr.
Neand., Afr.; Chimp., Afr.
Neand., Afr.; Chimp., nAfr.
Neand., nAfr.; Chimp., nAfr.
X, Y; Chimp., Den.
X, Y; Chimp., Neand.
X, Y; Neand., Den.
X, Chimp.; Neand., Den.

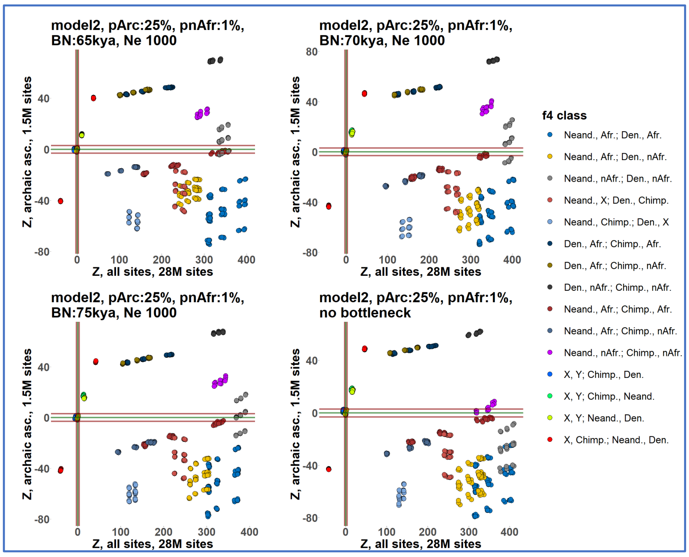
model2, pArc:25%, pnAfr:1%, BN:65kya, Ne 1000
model2, pArc:25%, pnAfr:1%, BN:70kya, Ne 1000
model2, pArc:25%, pnAfr:1%, BN:75kya, Ne 1000
model2, pArc:25%, pnAfr:1%, no bottleneck
Z, archaic asc., 1.5M sites
Z, all sites, 28M sites
f4 class
Neand., Afr.; Den., Afr.
Neand., Afr.; Den., nAfr.
Neand., nAfr.; Den., nAfr.
Neand., X; Den., Chimp.
Neand., Chimp.; Den., X
Den., Afr.; Chimp., Afr.
Den., Afr.; Chimp., nAfr.
Den., nAfr.; Chimp., nAfr.
Neand., Afr.; Chimp., Afr.
Neand., Afr.; Chimp., nAfr.
Neand., nAfr.; Chimp., nAfr.
X, Y; Chimp., Den.
X, Y; Chimp., Neand.
X, Y; Neand., Den.
X, Chimp.; Neand., Den.

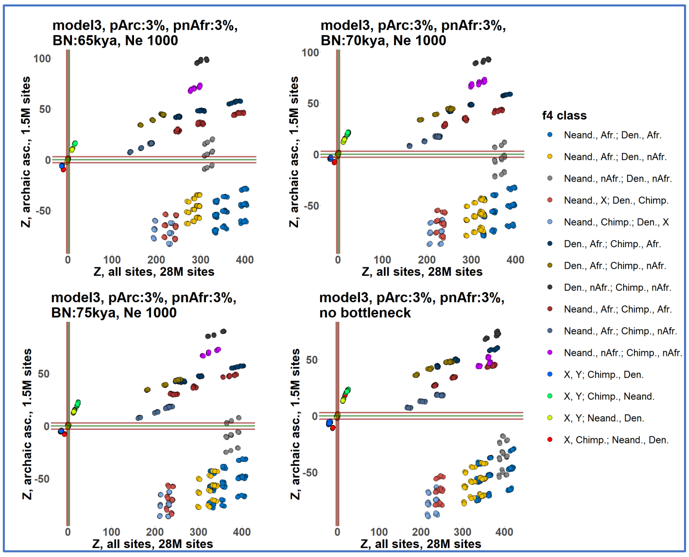
model3, pArc:3%, pnAfr:3%, BN:65kya, Ne 1000
model3, pArc:3%, pnAfr:3%, BN:70kya, Ne 1000
model3, pArc:3%, pnAfr:3%, BN:75kya, Ne 1000
model3, pArc:3%, pnAfr:3%, no bottleneck
Z, archaic asc., 1.5M sites
Z, all sites, 28M sites
f4 class
Neand., Afr.; Den., Afr.
Neand., Afr.; Den., nAfr.
Neand., nAfr.; Den., nAfr.
Neand., X; Den., Chimp.
Neand., Chimp.; Den., X
Den., Afr.; Chimp., Afr.
Den., Afr.; Chimp., nAfr.
Den., nAfr.; Chimp., nAfr.
Neand., Afr.; Chimp., Afr.
Neand., Afr.; Chimp., nAfr.
Neand., nAfr.; Chimp., nAfr.
X, Y; Chimp., Den.
X, Y; Chimp., Neand.
X, Y; Neand., Den.
X, Chimp.; Neand., Den.

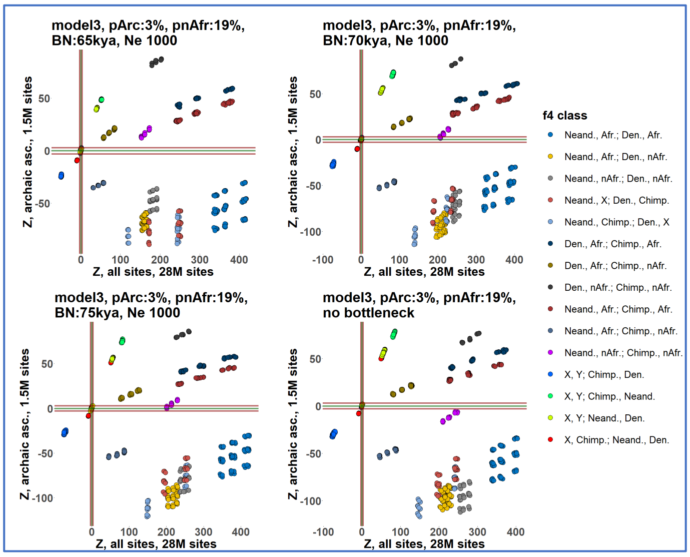
model3, pArc:3%, pnAfr:19%, BN:65kya, Ne 1000
model3, pArc:3%, pnAfr:19%, BN:70kya, Ne 1000
model3, pArc:3%, pnAfr:19%, BN:75kya, Ne 1000
model3, pArc:3%, pnAfr:19%, no bottleneck
Z, archaic asc., 1.5M sites
Z, all sites, 28M sites
f4 class
Neand., Afr.; Den., Afr.
Neand., Afr.; Den., nAfr.
Neand., nAfr.; Den., nAfr.
Neand., X; Den., Chimp.
Neand., Chimp.; Den., X
Den., Afr.; Chimp., Afr.
Den., Afr.; Chimp., nAfr.
Den., nAfr.; Chimp., nAfr.
Neand., Afr.; Chimp., Afr.
Neand., Afr.; Chimp., nAfr.
Neand., nAfr.; Chimp., nAfr.
X, Y; Chimp., Den.
X, Y; Chimp., Neand.
X, Y; Neand., Den.
X, Chimp.; Neand., Den.

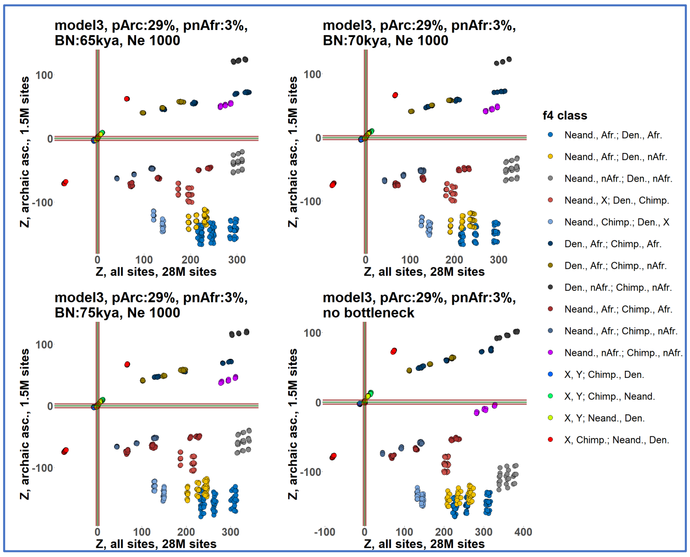
model3, pArc:29%, pnAfr:3%, BN:65kya, Ne 1000
model3, pArc:29%, pnAfr:3%, BN:70kya, Ne 1000
model3, pArc:29%, pnAfr:3%, BN:75kya, Ne 1000
model3, pArc:29%, pnAfr:3%, no bottleneck
Z, archaic asc., 1.5M sites
Z, all sites, 28M sites
f4 class
Neand., Afr.; Den., Afr.
Neand., Afr.; Den., nAfr.
Neand., nAfr.; Den., nAfr.
Neand., X; Den., Chimp.
Neand., Chimp.; Den., X
Den., Afr.; Chimp., Afr.
Den., Afr.; Chimp., nAfr.
Den., nAfr.; Chimp., nAfr.
Neand., Afr.; Chimp., Afr.
Neand., Afr.; Chimp., nAfr.
Neand., nAfr.; Chimp., nAfr.
X, Y; Chimp., Den.
X, Y; Chimp., Neand.
X, Y; Neand., Den.
X, Chimp.; Neand., Den.

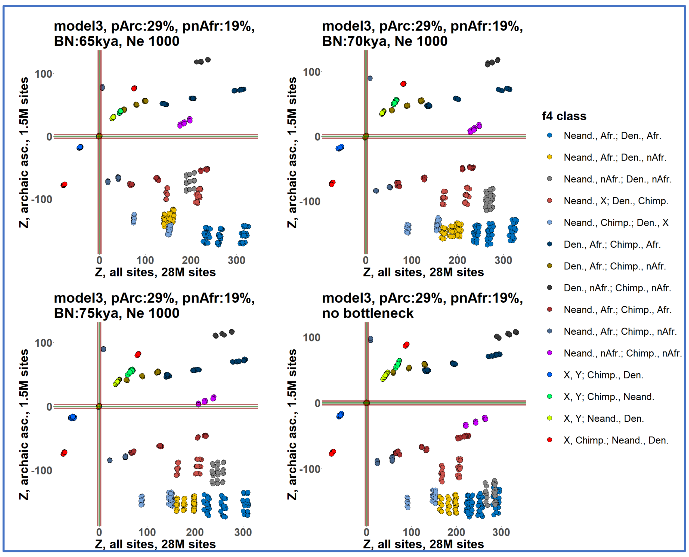
model3, pArc:29%, pnAfr:19%, BN:65kya, Ne 1000
model3, pArc:29%, pnAfr:19%, BN:70kya, Ne 1000
model3, pArc:29%, pnAfr:19%, BN:75kya, Ne 1000
model3, pArc:29%, pnAfr:19%, no bottleneck
Z, archaic asc., 1.5M sites
Z, all sites, 28M sites
f4 class
Neand., Afr.; Den., Afr.
Neand., Afr.; Den., nAfr.
Neand., nAfr.; Den., nAfr.
Neand., X; Den., Chimp.
Neand., Chimp.; Den., X
Den., Afr.; Chimp., Afr.
Den., Afr.; Chimp., nAfr.
Den., nAfr.; Chimp., nAfr.
Neand., Afr.; Chimp., Afr.
Neand., Afr.; Chimp., nAfr.
Neand., nAfr.; Chimp., nAfr.
X, Y; Chimp., Den.
X, Y; Chimp., Neand.
X, Y; Neand., Den.
X, Chimp.; Neand., Den.

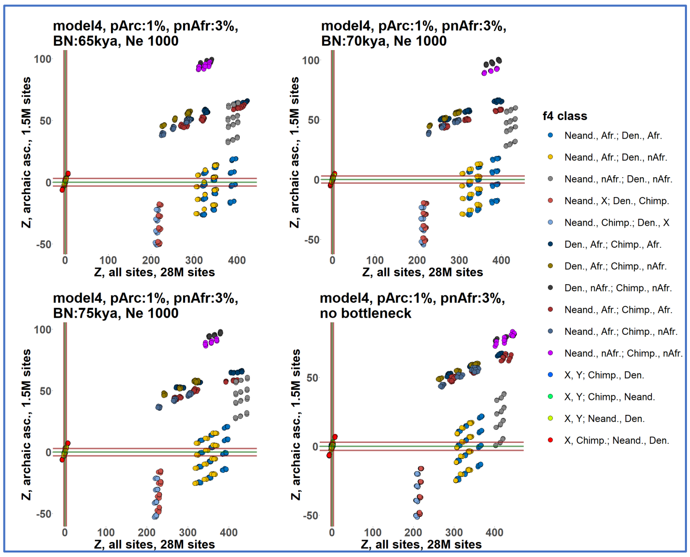
model4, pArc:1%, pnAfr:3%, BN:65kya, Ne 1000
model4, pArc:1%, pnAfr:3%, BN:70kya, Ne 1000
model4, pArc:1%, pnAfr:3%, BN:75kya, Ne 1000
model4, pArc:1%, pnAfr:3%, no bottleneck
Z, archaic asc., 1.5M sites
Z, all sites, 28M sites
f4 class
Neand., Afr.; Den., Afr.
Neand., Afr.; Den., nAfr.
Neand., nAfr.; Den., nAfr.
Neand., X; Den., Chimp.
Neand., Chimp.; Den., X
Den., Afr.; Chimp., Afr.
Den., Afr.; Chimp., nAfr.
Den., nAfr.; Chimp., nAfr.
Neand., Afr.; Chimp., Afr.
Neand., Afr.; Chimp., nAfr.
Neand., nAfr.; Chimp., nAfr.
X, Y; Chimp., Den.
X, Y; Chimp., Neand.
X, Y; Neand., Den.
X, Chimp.; Neand., Den.

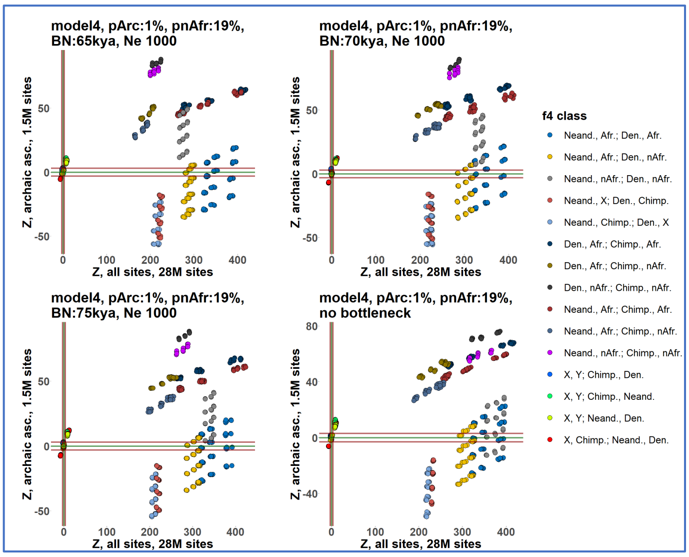
model4, pArc:1%, pnAfr:19%, BN:65kya, Ne 1000
model4, pArc:1%, pnAfr:19%, BN:70kya, Ne 1000
model4, pArc:1%, pnAfr:19%, BN:75kya, Ne 1000
model4, pArc:1%, pnAfr:19%, no bottleneck
Z, archaic asc., 1.5M sites
Z, all sites, 28M sites
f4 class
Neand., Afr.; Den., Afr.
Neand., Afr.; Den., nAfr.
Neand., nAfr.; Den., nAfr.
Neand., X; Den., Chimp.
Neand., Chimp.; Den., X
Den., Afr.; Chimp., Afr.
Den., Afr.; Chimp., nAfr.
Den., nAfr.; Chimp., nAfr.
Neand., Afr.; Chimp., Afr.
Neand., Afr.; Chimp., nAfr.
Neand., nAfr.; Chimp., nAfr.
X, Y; Chimp., Den.
X, Y; Chimp., Neand.
X, Y; Neand., Den.
X, Chimp.; Neand., Den.

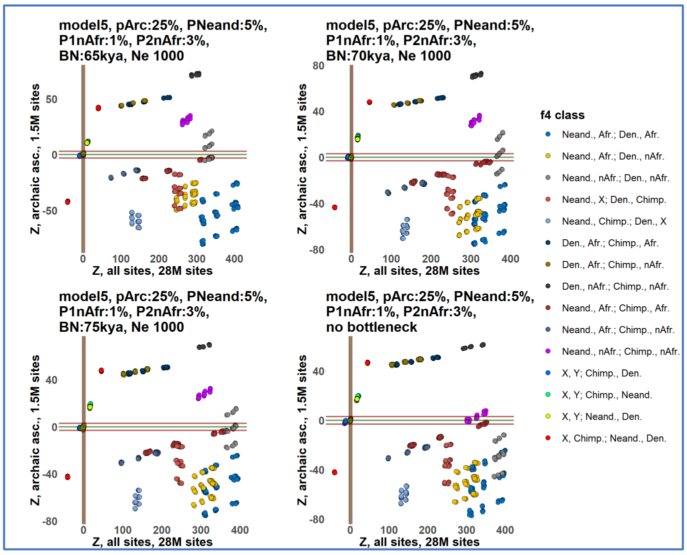
model5, pArc:25%, PNeand:5%, P1nAfr:1%, P2nAfr:3%, BN:65kya, Ne 1000
model5, pArc:25%, PNeand:5%, P1nAfr:1%, P2nAfr:3%, BN:70kya, Ne 1000
model5, pArc:25%, PNeand:5%, P1nAfr:1%, P2nAfr:3%, BN:75kya, Ne 1000
model5, pArc:25%, PNeand:5%, P1nAfr:1%, P2nAfr:3%, no bottleneck
Z, archaic asc., 1.5M sites
Z, all sites, 28M sites
f4 class
Neand., Afr.; Den., Afr.
Neand., Afr.; Den., nAfr.
Neand., nAfr.; Den., nAfr.
Neand., X; Den., Chimp.
Neand., Chimp.; Den., X
Den., Afr.; Chimp., Afr.
Den., Afr.; Chimp., nAfr.
Den., nAfr.; Chimp., nAfr.
Neand., Afr.; Chimp., Afr.
Neand., Afr.; Chimp., nAfr.
Neand., nAfr.; Chimp., nAfr.
X, Y; Chimp., Den.
X, Y; Chimp., Neand.
X, Y; Neand., Den.
X, Chimp.; Neand., Den.

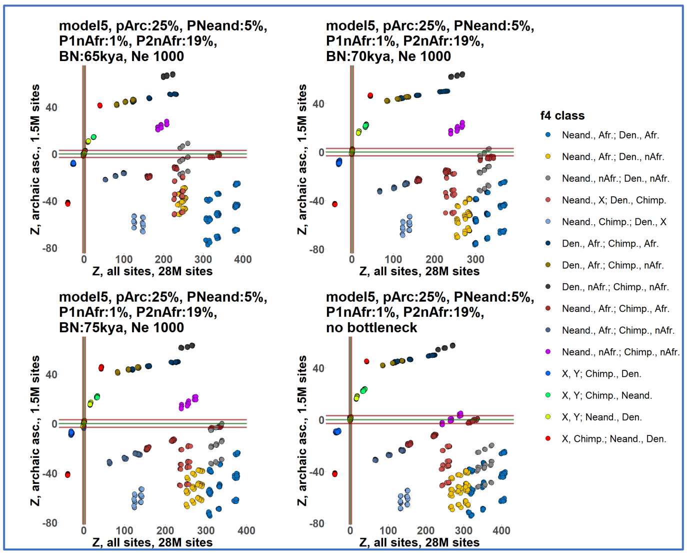
model5, pArc:25%, PNeand:5%, P1nAfr:1%, P2nAfr:19%, BN:65kya, Ne 1000
model5, pArc:25%, PNeand:5%, P1nAfr:1%, P2nAfr:19%, BN:70kya, Ne 1000
model5, pArc:25%, PNeand:5%, P1nAfr:1%, P2nAfr:19%, BN:75kya, Ne 1000
model5, pArc:25%, PNeand:5%, P1nAfr:1%, P2nAfr:19%, no bottleneck
Z, archaic asc., 1.5M sites
Z, all sites, 28M sites
f4 class
Neand., Afr.; Den., Afr.
Neand., Afr.; Den., nAfr.
Neand., nAfr.; Den., nAfr.
Neand., X; Den., Chimp.
Neand., Chimp.; Den., X
Den., Afr.; Chimp., Afr.
Den., Afr.; Chimp., nAfr.
Den., nAfr.; Chimp., nAfr.
Neand., Afr.; Chimp., Afr.
Neand., Afr.; Chimp., nAfr.
Neand., nAfr.; Chimp., nAfr.
X, Y; Chimp., Den.
X, Y; Chimp., Neand.
X, Y; Neand., Den.
X, Chimp.; Neand., Den.
