# Supplementary material for: Modeling of African population history using f-statistics is biased when applying all previously proposed SNP ascertainment schemes
Source: PLoS Genet. 2023 Sep 7;19(9):e1010931. doi: 10.1371/journal.pgen.1010931 (PMC10508636; doi:10.1371/journal.pgen.1010931)
Supplement: S13 Table — (PDF) [file pgen.1010931.s030.pdf]

S13 Table

Simulated topology	Population name or event name	Effective population size, diploid individuals	Sampling date, generations	Event date, generations
**16b Fig**	Chimpanzee	1,000	0	
	Denisovan	3,000	2,000	
	Neanderthal	3,000	2,000	
	African 1	22,500	0	
	African 2	22,500	0	
	African 3	22,500	0	
	African 4	22,500	0	
	African 5	22,500	0	
	Non-African 1	5,000	0	
	Non-African 2	5,000	0	
	Non-African 3	5,000	0	
**Fig 3a**	a0 (ancestor of a2 + na1 + na2)	16,914	N/A	
	African 1 (a1)	44,541	0	
	African 2 (a2)	46,139	0	
	eff. pop. size after the out-of-Africa bottleneck	1,506	N/A	
	Denisovan (d)	16,758	1,700	
	Neanderthal 1 (n1)	14,399	3,790	
	Neanderthal 2 (n2)	14,399	1,700	
	AMH (a1 + a2 + na1 + na2)	222,379	N/A	
	ancestral Neanderthal population	8,145	N/A	
	ancestral non-African population (na1 + na2)	8,821	N/A	
	non-African 1 (na1)	35,744	0	
	non-African 2 (na2)	14,763	0	
	Neanderthal + AMH ancestral population	86,161	N/A	
	super-archaic population	35,414	N/A	
	chimpanzee (outgroup)	1,000	N/A	
	root population (chimpanzee + archaic + AMH)	13,858	N/A	
	chimpanzee divergence			240,000
	divergence of the ghost population contributing 19% of ancestry in AMH			34,740
	gene flow from the ghost lineage to AMH			11,661
	divergence of the deeper source of Denisovan ancestry			29,215
	divergence of the Neanderthal lineage (n1+n2)			20,255
	divergence of the ghost Neanderthal lineage admixing into Denisovans			15,946
	gene flow from the ghost Neanderthal lineage to Denisovans			7,311
	divergence of the ghost AMH lineage admixing into non-Africans			14,184
	African 1 (a1) divergence			6,314
	divergence of the Neanderthal 1 (n1) and Neanderthal 2 (n2) lineages			4,013
	African 2 (a2) divergence			3,395
	out-of-Africa bottleneck			2,931
	gene flow from the Neanderthal 2 (n2) lineage to the AMH ghost lineage			2,820
	gene flow from the AMH ghost lineage to non-Africans			2,544
	divergence of the non-African 1 (na1) and non-African 2 (na2) lineages			1,976
