# Supplementary material for: Modeling of African population history using f-statistics is biased when applying all previously proposed SNP ascertainment schemes
Source: PLoS Genet. 2023 Sep 7;19(9):e1010931. doi: 10.1371/journal.pgen.1010931 (PMC10508636; doi:10.1371/journal.pgen.1010931)
Supplement: S1 Text — (PDF) [file pgen.1010931.s031.pdf]

S1 Text

Effects of ascertainment on real data: fits of complex admixture graphs

Lipson *et al*. (2020) constructed a complex admixture graph for Africans, including the Altai Neanderthal and chimpanzee. The model was built manually on a dataset derived from the 1240K panel, and a final model was also confirmed to fit the union of the Human Origins sub-panels 4 and 5 or archaic-ascertained sites. We aimed at reassessing these results in the light of *f*-statistic biases and took advantage of a new algorithm for automated search of the admixture graph space, *findGraphs* (Maier et al. 2023). Since the final model in that study is rather complex (12 populations including chimpanzee as an outgroup and 12 admixture events, which results in a space of up to 1.22×10^{42} topologies), we focused on a simpler intermediate model shown in that study. The simpler model consists of 10 groups and 8 admixture events (Lipson et al. 2020, Fig S3.25 in that study). The corresponding space of all possible graphs is still impressively large: up to 1.9×10^{26} topologies. To enable comparison of various ascertainment schemes on whole-genome shotgun data, the population composition of the dataset from Lipson *et al.* was slightly modified: the ancient South African hunter-gatherer group was replaced by a related group, present-day Ju|'hoan North from South Africa, and instead of the Shum Laka ancient group from Cameroon only one shotgun-sequenced individual from that group (I10871) was used.

We began by fitting the published 10-population model with 8 admixture events to three SNP sets without missing data at the group level: 1) 1240K, ca. 839,000 polymorphic sites; 2) AT/GC mutation types, ca. 3.8 million polymorphic sites; and 3) unascertained data, ca. 25 million polymorphic sites. The published model had widely different fits on these three datasets (**S4a Fig**): The worst f_4-statistic residual (WR) of the published admixture graph model was 2.7 SE on the 1240K dataset, 4.8 SE on AT/GC sites, and 8.4 SE on unascertained data.

Next, we attempted to find alternative well-fitting models in the same complexity class (i.e., 10 groups and 8 admixture events) on each of the three datasets and refitted them on the other two datasets. The *findGraphs* topology search algorithm was started 10,000 times from

random graphs with chimpanzee set as an outgroup, and that procedure was repeated for each SNP set. For simplicity, only one graph with the best log-likelihood score (LL) was taken from each *findGraphs* run. The fits (both LL and WR) of the resulting collections of ca. 10,000 distinct newly found admixture graph topologies are summarized in **S4b Fig**. Fits of these graphs on the 1240K panel and on all sites are correlated poorly (Pearson's *R* for LL ranging from 0.35 to 0.46), and the fits on AT/GC sites and on all sites are correlated better (*R* for LL ranging from 0.58 to 0.73, **S5a Fig**); this situation resembles our observations on collections of much simpler admixture graphs (**Figs 1 and 2, Table 1**). Among thousands of topologies inferred on the 1240K dataset, 63% fit the 1240K data well (WR <3 SE), but all of them do not fit the unascertained dataset (WR range from 4.6 to 21 SE, **S5b Fig**). The converse analysis also reveals concerns: of 24 topologies found that fit the unascertained dataset relatively well (WR between 3 and 4 SE), 18 topologies fit the 1240K dataset worse (WR >4 SE) despite its much smaller size, while 6 topologies fit both datasets with WR between 3 and 4 SE. These results are in line with those for the simpler graphs in **Figs 1 and 2**, where topologies that fit the unascertained data and do not fit the ascertained data by a wide margin (and *vice versa*) are highlighted. Similar results were obtained for a smaller published intermediate graph (Lipson et al. 2020, Fig S3.24) with 7 groups and 4 admixture events (**S5c and S5d Figs**).
