# Supplementary material for: Modeling of African population history using f-statistics is biased when applying all previously proposed SNP ascertainment schemes
Source: PLoS Genet. 2023 Sep 7;19(9):e1010931. doi: 10.1371/journal.pgen.1010931 (PMC10508636; doi:10.1371/journal.pgen.1010931)
Supplement: S2 Text — (PDF) [file pgen.1010931.s032.pdf]

S2 Text

An overview of f_4-statistic biases caused by ascertainment

The following group of f_4-statistics was explored exhaustively: statistics including Africans (unadmixed with West Eurasians according to Fan *et al*. 2019, see group annotations in **S1 Table**) and/or archaic humans and/or Mediterranean/Middle Eastern groups (abbreviated as "Med/ME"). This group of ca. 1.37 million statistics was subdivided into 14 classes depending on the composition of the population quadruplet: for instance, all statistics including three African groups and an archaic individual; or two African groups, an archaic individual and a Med/ME group, etc. In the analyses below we focused on f_4-statistics that do not deviate from 0 too far (absolute Z-score on unascertained data is <15 SE) since in practice it is often important if an f_4-statistic is consistent with 0 on a certain set of sites but deviates from 0 by >3 SE on another dataset, and especially if it changes its sign.

Several f_4-statistic classes were affected by ascertainment, as illustrated by distributions of absolute Z-score differences (**S12 Fig**) and by scatterplots of Z-scores on unascertained vs. ascertained data (**S13 Fig**). The unascertained dataset ("all sites") was used as a baseline in all cases. By far the worst performing f_4-statistic class was f_4(African$_x$, archaic; African$_y$, Med/ME). All statistics belonging to this class are biased in the case of the 1240K ascertainment since none of them lie on the diagonal (**Fig 6**); and all eight non-random ascertainment schemes explored in this analysis, including archaic ascertainment schemes, demonstrated similar patterns (**S12 Fig**). This observation is in line with the results on the simulated history chosen as a case study: there statistics f_4("African 1", "archaic"; "African 2", "non-African") are affected by all types of ascertainments tested (in the presence of an archaic to non-African gene flow, see **S6b Fig**).

The following three f_4-statistic classes were also affected by bias, but were less problematic than f_4(African$_x$, archaic; African$_y$, Med/ME) according to residual standard deviation of linear model (abbreviated as "residual SE" and expressed in the same units as f_4-statistic Z-scores): 1) f_4(African$_x$, African$_y$; African$_z$, archaic), 2) f_4(African$_x$, African$_y$; African$_z$, Med/ME) (**Fig 6**), 3)

f_4(African$_w$, African$_x$; African$_y$, African$_z$) (**S12 and S13 Figs**). Notably, removal of variants rare in Africans helped to mitigate the bias in the case of the latter three f_4-statistic classes almost completely (**Figs 6, S12, and S13**). Archaic ascertainment helped to shift modes of the |Z-score difference| distributions towards 0 for all the classes except for f_4(African$_x$, archaic; African$_y$, Med/ME), however long distribution tails, i.e. a high noise level, observed for 7 f_4-statistic classes such as f_4(African$_x$, African$_y$; African$_z$, archaic) and f_4(African$_w$, African$_x$; African$_y$, African$_z$) (**S12 and 13 Figs**) suggest that this ascertainment scheme performs worse in practice than the AFR MAF ascertainment.

Next, we demonstrated that among ca. 94,000 randomly selected statistics f_4(African$_x$, archaic; African$_y$, non-African), all deviate from the diagonal by at least 2 SE under the 1240K ascertainment (**S14 Fig**; all continental-scale populations of non-Africans in the SGDP dataset were tested, including such groups as Papuans and the Upper Paleolithic Ust'-Ishim individual, but excluding Med/ME groups tested above). We also demonstrated on exhaustive collections of f_4-statistics that do not include archaic individuals or African groups only (statistics including various combinations of African and East Asian groups pooled with statistics including various combinations of American/Siberian and/or European and/or Papuan groups) that the effect of the AFR MAF ascertainment is not distinguishable from that of random dataset thinning (**S15 Fig**). In contrast, the 1240K ascertainment, HO panels 4, 5, 13, 4+5, and archaic ascertainment resulted in a fraction of f_4-statistic Z-scores deviating from the diagonal by > 2 SE (**S15 Fig**).

According to our mass comparison of ascertainment schemes on 27 f_4-statistic classes (**S7 Table**), the AFR MAF ascertainment outperformed all other ascertainment schemes explored, including archaic ascertainment, and the same result was observed when exploring admixture graph model fits (**S2 Fig, Tables 1 and S3-S5**). For this comparison we disregarded f_4-statistics with |Z| > 15 SE on all sites, and thus removed all extreme outliers in the f_4(Neanderthal, X; Denisovan, Y) statistic classes that emerge due to the fact that the same pair of individuals, Neanderthal and Denisovan, were used both for ascertainment and for calculating *f*-statistics, as we show on simulated (**Figs 3b and S17c**) and real data (**S17a Fig**). The existence of these outliers (i.e., statistics changing their sign from highly positive to highly negative, **S17 Fig**) serves as another argument in favor of the AFR MAF ascertainment over archaic ascertainment (see,

e.g., **S11a Fig**) since the former is applicable to a wider range of population sets. And due to the paucity of high-coverage archaic genomes (Meyer et al. 2012, Prüfer et al. 2014, Mafessoni et al. 2020), it is often unavoidable that the same individuals are used for both ascertainment and for calculating *f*-statistics or for fitting admixture graphs.

On simulated data we focused on two types of f_4-statistics that, as we found empirically, are most affected by ascertainment: f_4("African 1", "archaic individual"; "African 2", "non-African 1 or 2") (**S6b Fig**) and f_4("non-African 1", "archaic individual"; "African 1 or 2", "non-African 2") (**S6c Fig**). In the absence of Neanderthal gene flow to non-Africans, the Z-scores of the former statistics are not affected substantially by any ascertainment scheme we applied: Z-scores across nearly all simulation iterations, population combinations, and ascertainment schemes remain significantly negative (<–3 SE). However, in the presence of the Neanderthal gene flow the Z-scores for f_4("African 1", "archaic individual"; "African 2", "non-African 1 or 2") are shifted from significantly positive to nearly 0, or from 0 to significantly negative under the following ascertainment schemes: HO one-panel (ascertained on an individual from the "African 2" group), AFR MAF, and archaic ascertainment(**S6b Fig**). This result suggest that the universally strong bias in this statistic class observed under all ascertainment schemes tested on real data is due to the Neanderthal to non-African gene flow.
