# Supplementary material for: Modeling of African population history using f-statistics is biased when applying all previously proposed SNP ascertainment schemes
Source: PLoS Genet. 2023 Sep 7;19(9):e1010931. doi: 10.1371/journal.pgen.1010931 (PMC10508636; doi:10.1371/journal.pgen.1010931)
Supplement: S3 Text — (PDF) [file pgen.1010931.s033.pdf]

S3 Text

Mechanisms of bias in selected f_4-statistics

To understand mechanisms underlying the biases that we documented, several individual f_4-statistics were analyzed in detail. We considered a statistic of the most biased class f_4(African$_x$, archaic; African$_y$, non-African), specifically f_4(Altai Neanderthal, Biaka; Mbuti, Saharawi) (Saharawi is an African group with a low proportion of sub-Saharan African ancestry [1]). In **S9 Table** f_4-statistic values and Z-scores are shown for the unascertained site set and across nine ascertainment schemes.

In **S10 Table** the statistic f_4(Altai Neanderthal, Biaka; Mbuti, Saharawi) is explored across the derived allele frequency (DAF) spectrum and on six site sets: 1) unascertained data; 2) AT/GC sites; 3) 1240K; 4) global MAF; 5) AFR MAF (considering Africans unadmixed with non-Africans, **S1 Table**); and 6) archaic ascertainment. Alleles were classified as derived or ancestral according to the pseudohaploid chimpanzee genome, and DAF was defined either on the African meta-population (unadmixed with non-Africans) or on various non-African meta-populations. Since results were similar for those non-African meta-populations, results for the European meta-population only are shown in **S10 Table**. For simplicity, sites were stratified by DAF into three bins: nearly fixed ancestral (DAF ≤5%), intermediate frequency (DAF 5-95%), and nearly fixed derived (DAF ≥95%). No sites with missing allele frequencies were allowed at the group level in Altai Neanderthal, Biaka, Mbuti, Saharawi and at the meta-population level in Africans.

First, we consider results on unascertained data and on AT/GC mutation classes, which are nearly identical (**S10 Table**). As expected for a relatively complex demographic history with gene flows and bottlenecks [2], the statistic f_4(Altai Neanderthal, Biaka; Mbuti, Saharawi) is highly variable across the three DAF bins (**S10 Table**). On sites with intermediate DAF in Africans, the statistic is highly positive, and on much more numerous sites with nearly fixed ancestral or derived alleles the statistic is mildly negative. Since sites with nearly fixed ancestral or derived variants predominate in the unascertained dataset (**S10 Table**), the statistic

approaches 0 on all sites (**S9 Table**). All the types of non-random ascertainment explored here increase dramatically the proportion of sites with intermediate DAF: it reaches 7.8% for unascertained data or AT/GC sites and varies between 22.5% and 98.7% for non-randomly ascertained sites. The proportion of nearly fixed sites, especially those with nearly fixed derived alleles, drops dramatically under non-random ascertainment: from 28-31% to 0.1-8.4% (**S10 Table**). Since the statistic f_4(Altai Neanderthal, Biaka; Mbuti, Saharawi) is highly variable across the DAF spectrum, discarding a great majority of nearly fixed sites shifts its value. As we showed on simulated data above (**Fig 3b**), the fact that archaic human lineages do not represent a true outgroup for AMH makes the class of statistics f_4($African_x$, archaic; $African_y$, non-African) especially problematic, i.e., biased under all ascertainment types tested in this study (**Fig 5a**).

But distortion of the DAF spectrum is not the only effect that non-random ascertainment schemes have. Within each DAF bin, ascertained sites and all sites show different f_4-statistic values (**S10 Table**). For instance, in the intermediate bin (DAF in Africans) the statistic f_4(Altai Neanderthal, Biaka; Mbuti, Saharawi) equals 0.0064 for the 1240K sites and 0.0021 for all sites (Z-scores = 14 and 6, respectively). The same is true for the nearly fixed bins and for DAF based on the European meta-population (**S10 Table**). Global MAF ascertainment (removal of variants that are rare across all 350 individuals, i.e., that have MAF <5%) produces a comparable shift in the same direction (**S10 Table**). Among individuals in the SGDP dataset, 73% are non-Africans (**S1 Table**), and variants common in non-Africans are favored by this ascertainment scheme. In other words, the global MAF ascertainment does not shift average DAF across all four populations involved in the statistic in the same way: both average $DAF_{Altai\text{-}Biaka}$ and especially average $DAF_{Mbuti\text{-}Saharawi}$ in the intermediate DAF bin move in the negative direction under ascertainment, which reflects relative paucity of derived variants in the Neanderthal and relative excess of derived variants in Saharawi under this ascertainment (**S10 Table**).

As stated above, similar effects are observed under the 1240K ascertainment (**S10 Table**), and we argue that the same explanation holds for the global MAF and 1240K ascertainment schemes. Although ca. 59% of sites on the Human Origins array were ascertained on African individuals (San, Yoruba, and Mbuti), the 1240K panel is a complex construct where

approximately half of sites are derived from the Illumina 650Y and Affymetrix 50k arrays [3], themselves products of complex ascertainment based mostly on Eurasian populations. Thus, derived variants common in non-Africans but rare in Africa and in archaic humans are overrepresented on the 1240K panel, which skews f_4-statistics within the DAF bins. In contrast, under archaic ascertainment average $DAF_{Altai\text{-}Biaka}$ moves in the positive direction in the intermediate and "nearly fixed ancestral" bins, and those bins account for >90% of ascertained sites in this case (**S10 Table**). Archaic ascertainment by definition favors derived variants common in archaic humans but does not favor derived variants common in AMH, thus $DAF_{Altai\text{-}Biaka}$ becomes highly positive in these bins, and the resulting f_4-statistic becomes highly negative (**S9 and S10 Tables**).

We also considered statistics that are among the most distant outliers in two other biased classes: f_4(Fulani, Ju|'hoan North; Igbo, Ogiek) of the f_4($African_w$, $African_x$; $African_y$, $African_z$) class, and f_4(Burmese, Dinka; Ju|'hoan North, Sengwer) of the f_4($African_x$, $African_y$; $African_z$, East Asian) class. These two statistics show similar patterns: the bias in Z-scores detected under the 1240K and global MAF ascertainment is much smaller or non-existent under the AFR MAF and archaic ascertainments (**S9 Table**) despite the depletion of nearly fixed derived sites common for all these datasets (**S11 and S12 Tables**). In the case of the statistics f_4(Fulani, Ju|'hoan North; Igbo, Ogiek) and f_4(Burmese, Dinka; Ju|'hoan North, Sengwer), the 1240K and global MAF ascertainments also have very similar effects within DAF bins, especially within the "nearly fixed ancestral" bin where both statistics move in the negative direction (**S11 and S12 Tables**). A smaller effect with the same direction is observed in the intermediate DAF bin. Thus, the shift in these f_4-statistics can be explained by unequal enrichment for derived variants across the four populations. Since the AFR MAF and archaic ascertainment schemes do not favor derived variants of non-African origin, the bias is much smaller or non-existent in those cases (**S9 Table**).

References

1. Fan S, Kelly DE, Beltrame MH, Hansen MEB, Mallick S, Ranciaro A, et al. African evolutionary history inferred from whole genome sequence data of 44 indigenous African populations. Genome Biol. 2019;20: 82. doi: 10.1186/s13059-019-1679-2.

2. Martin SH, Amos W. Signatures of introgression across the allele frequency spectrum. Mol Biol Evol. 2020;38: 716–726. doi: 10.1093/molbev/msaa239.
3. Fu Q, Hajdinjak M, Moldovan OT, Constantin S, Mallick S, Skoglund P, et al. An early modern human from Romania with a recent Neanderthal ancestor. Nature. 2015;524: 216–219. doi: 10.1038/nature14558.
