# Supplementary material for: Modeling of African population history using f-statistics is biased when applying all previously proposed SNP ascertainment schemes
Source: PLoS Genet. 2023 Sep 7;19(9):e1010931. doi: 10.1371/journal.pgen.1010931 (PMC10508636; doi:10.1371/journal.pgen.1010931)
Supplement: S4 Text — (PDF) [file pgen.1010931.s034.pdf]

S4 Text

Archaic ascertainment explored on simulated genetic data

We simulated various scenarios of gene flow between archaic humans and AMH. Whole human genomes evolving via mutation, recombination, and drift were simulated using *msprime* v.0.7.4 [1]. We simulated five different topologies that are not intended to cover the whole diversity of possible topologies, and different gene flow intensities were tested for all the topologies (see a complete list of parameters tested in **S16b and S17b Figs**). We also varied the time and intensity of the out-of-Africa bottleneck (**S16b and S17b Figs**). Each simulation included "chimpanzee", "Denisovan" and "Neanderthal" populations composed of one sampled diploid individual each, five paraphyletic "African" groups (10 diploid individuals sampled per group) with intra-African gene flows, and three monophyletic "non-African" groups (10 diploid individuals sampled per group) connected with gene flows too (see detailed illustrations of model parameters in **S16b and S17b Figs** and in **S13 Table**). The simulated African and non-African groups diverged 100 kya (given a generation time of 25 years). For all simulated histories, SNPs polymorphic in a group composed of the Neanderthal and Denisovan individuals (two individuals in total) were taken as the ascertained set. f_4-statistics including the same Neanderthal and Denisovan individuals, and those including at least one of these archaic individuals and chimpanzee were calculated for the simulated groups, and results for the five simulated topologies are shown in **S16c and S17c Figs**.

Under the simplest simulation scenario (labelled as "model 1 pArc:NA pnAfr:0%"), with no gene flow from the Neanderthal lineage to the common ancestor of non-Africans, several classes of statistics that can be described as f_4(Neanderthal, X; Denisovan, Y) change their sign under archaic ascertainment from highly positive to highly negative (**S17c Fig**). The bias here emerges due to the ascertainment procedure itself since the same pair of individuals, Neanderthal and Denisovan, were used both for ascertainment and for calculating *f*-statistics. Introducing a single archaic gene flow to non-Africans (3% of their ancestry derived from the Neanderthal branch at 70 kya) affects the patterns of outliers under archaic ascertainment

(**S17c Fig**, "model 1 pArc:NA pnAfr:3%"), and it becomes more similar to that observed on real data (**S17a Fig**). f_4-statistics calculated on real data (AT/GC sites vs. archaic ascertainment) did not include Papuans and Australians since results for those populations would be affected by the Denisovan admixture that was not simulated; they also do not include Africans having substantial non-African admixture (**S1 Table**), to make distinction between f_4-statistic classes clearer. Few differences from the pattern observed on real data are as follows: 1) statistics f_4(Neanderthal, African; chimpanzee, non-African) change their sign on simulated data but do not change their sign on real data; 2) all statistics f_4(Neanderthal, non-African$_x$; Denisovan, non-African$_y$) change their sign on simulated data, but some do not change their sign on real data (**S17a Fig**).

Next, we simulated a model of archaic introgression proposed by Durvasula and Sankararaman [2] with 4 relevant gene flows (labelled as "model 2"). Here we tested 7 combinations of super-archaic ancestry proportion in AMH and Neanderthal ancestry proportion in non-Africans, in addition to four settings for the out-of-Africa bottleneck (**S17b Fig**). For large proportions of super-archaic ancestry in AMH (19%, the upper bound found in the original study, or 25%), the results on simulated and real data differed for more than two classes of statistics (**S17c Fig**). For a small proportion of super-archaic ancestry in AMH (2%, the lower bound found in the original study), the results on simulated (**S17c Fig**) and real data (**S17a Fig**) differ for two classes of statistics: statistics f_4(Neanderthal, African; chimpanzee, non-African) change their sign on simulated data under archaic ascertainment but do not show this behavior on real data; and statistics f_4(Neanderthal, X; Denisovan, chimpanzee) and f_4(Neanderthal, chimpanzee; Denisovan, X) form two distant clusters on simulated data, but are clustered closely together on real data (**S17c Fig**, "model 2, PArc 2%, PnAfr 3%").

Next, we tested three alternative simulated graphs, outcomes of an automated admixture graph inference procedure on real data (AT/GC sites, see Methods for a description of the *findGraphs* protocol). The best-scoring topology on real data (labelled as "model 3") includes two gene flows from a divergent Neanderthal-related lineage (splits from the Neanderthal lineage *sensu stricto* at 350 kya in our simulations): a flow into the common ancestor of AMH at 250 kya and another flow into the common ancestor of non-Africans at 70 kya (**S17b Fig**). If

both gene flow proportions remain low (3%), this topology reproduces the pattern of outliers observed on real data closely (**S17c Fig**, "model 3, PArc 3%, PnAfr 3%"): statistics f_4(Neanderthal, African; chimpanzee, non-African) do not change their sign, and statistics f_4(Neanderthal, X; Denisovan, chimpanzee) and f_4(Neanderthal, chimpanzee; Denisovan, X) cluster together. However, results for one class of statistics still disagree on simulated (**S17c Fig**) and real data (**S17a Fig**): many statistics f_4(X, Y; chimpanzee, Denisovan) change their sign on real data, but do not do that on simulated data. We also tested two other topologies that emerged among the best outcomes of the automated graph inference procedure on real data, labelled as "model 4" and "model 5" (**S17b Fig**), and in those cases patterns of outliers differ from those observed on real data for several classes of f_4-statistics.

Our observations on a narrow region of parameter space suggest that neither the simplest model of archaic introgression in AMH, i.e., the low-level pulse-like gene flow from Neanderthals to non-Africans, nor the more complex model proposed by Durvasula and Sankararaman [2], are able to predict the behavior of f_4-statistics under archaic ascertainment. These results on simulated data also suggest that since archaic humans do not represent a true outgroup for AMH (due to various gene flows between archaic humans and AMH), archaic ascertainment should be used with caution for reconstructing population history of AMH.

References

1. Kelleher J, Etheridge AM, McVean G. Efficient coalescent simulation and genealogical analysis for large sample sizes. PLOS Comput Biol. 2016;12: 1–22. doi: 10.1371/journal.pcbi.1004842.
2. Durvasula A, Sankararaman S. Recovering signals of ghost archaic introgression in African populations. Sci Adv. 2020;6: eaax5097. doi: 10.1126/sciadv.aax5097.
