# Supplementary material for: Modeling of African population history using f-statistics is biased when applying all previously proposed SNP ascertainment schemes
Source: PLoS Genet. 2023 Sep 7;19(9):e1010931. doi: 10.1371/journal.pgen.1010931 (PMC10508636; doi:10.1371/journal.pgen.1010931)
Supplement: Attachment — Submitted filename: rebuttal_2023_07_09.pdf [file pgen.1010931.s035.pdf]

As all three reviewers pointed out, the authors conducted an exhaustive analysis to investigate and characterize the ascertainment bias and its impact on inferring evolutionary history of African populations. All reviewers also have suggested that the manuscript as currently written is difficult to follow, both at the level of the text and the figure. They have made a number of suggestions to improve the readability of the manuscript. A revision with improved readability for a readership with general knowledge of genetics will be considered.

Reviewer's Responses to Questions

Comments to the Authors:
Please note here if the review is uploaded as an attachment.

Reviewer #1: Flegontov et al present a very detailed study of the impact of ascertainment on f4 statistics. This is an interesting and important study because f4 statistics are very commonly used in population genetic demography reconstruction in humans and many other taxa. They're particularly commonly used with ancient DNA, which is often sequenced based on a SNP-capture approach, due to the low quality data available. The authors are particularly concerned about ancient DNA from arid and hot regions, such as Africa, where shotgun ancient DNA is particularly unlikely to succeed and capture approaches are especially necessary.

Unfortunately, one of the authors primary findings is that the commonly used 1240K capture array is particularly bad at analyzing African populations. The authors do an absolutely heroic amount of work to explore a number of different ascertainment schemes both using empirical data and simulations. This leads them to having a wide range of suggestions depending on the data at hand, but also pointing out that ascertained data may ultimately make it very difficult to analyze some kinds of African population history---it's very difficult to alleviate the bias with any kind of ascertainment scheme.

We thank the reviewer for this favorable assessment of our work!

One question I have regarding the authors suggestions for future research is whether they are suggesting creating a new SNP array, or subsetting an existing one based on the schemes described here, or some mixture of the two. Not a major point per se, but I'd just like some clarity from the authors.

We do highlight the advantages of including a new SNP enrichment reagent (see the last paragraph in Discussion, lines 775-789) based on the "African MAF" ascertainment scheme we proposed because that would solve most bias issues and also statistical power issues, although balanced against this is the cost or producing such a SNP enrichment reagent and capturing libraries with it. Subsetting (e.g., using Human Origins panels 4 and 5 combined) performs relatively well in terms of bias, but has limited power to reject alternative models since the number of SNPs in these panels is small (see lines 352-354).

I don't find the analyses presented to be objectionable, and they largely seem to make sense to me. My most major concern with the manuscript is that I suspect it will be very hard to read for someone who isn't deeply versed in the lore of f-statistics. I think that for general readability and to reach a broader audience, it would be useful if every analyses had a brief introduction to explain exactly what the f-statistic represents and how it helps answer the question at hand. I think that will also help readers gain a little bit more familiarity with what is going on and provide a little more intuition as to what might be going on.

We have added these explanations throughout the text, and added two paragraphs introducing *f*-statistics (lines 89-126) and admixture graphs (lines 213-233) in the Introduction.

For myself, I don't understand the analysis described on line 449. What's the predictor and the response variable in the linear trend for f4 statistic z-scores? I suspect this is a thing a reader that is intimately familiar with f4 statistics will understand immediately but will be lost on a more general audience.

The predictor variable is simply Z-score for an f_4-statistic on unascertained data, and the response variable is Z-score for the same f_4-statistic on ascertained data. A linear relationship is expected between f_4-statistics on non-ascertained and ascertained data.

We have rephrased for clarity, see lines 572-578:

“For this analysis we used residual standard deviation ("residual SE") of a linear trend as a way of measuring correlation between f_4-statistic Z-scores on all sites and under ascertainment. We found this metric more convenient than the squared Pearson correlation coefficient (R^2) since it is expressed in the same units as Z-scores and thus is an intuitive way of representing deviation of f_4-statistic sets on ascertained data from those on unascertained data. We note that it reflects both bias introduced by ascertainment and variance generated by random site sampling.”

To highlight the fact that in all cases we are talking about various measures of linear correlation (residual SE or R^2) between f_4-statistics, or their Z-scores, or admixture graph fits on unascertained vs. ascertained data, we have modified the text and figure legends in many places.

Similarly, it might be worth noting that the f4 statistic for treeness (line 425) is basically the ABBA-BABA test that detected Neandertal admixture in humans, to help orient readers.

Thanks for this suggestion! We have mentioned that in the Introduction and in Results, see lines 98-100 and 545-547:

“The f_4-statistic is identical to the ABBA/BABA statistic, also known as the *D*-statistic (Green et al. 2010, Durand et al. 2011), up to a normalization factor and is a test for treeness.”

“f_4-statistics are tests for treeness that are essentially the same (Patterson et al. 2012) as the ABBA-BABA test (*D*-statistic, Green et al. 2010, Durand et al. 2011) which was used to detect Neanderthal admixture in non-Africans (Green et al. 2010).”

Overall, I do really like the paper and it's an amazing amount of work. I'm always happy to see more thorough explorations of the properties of f-statistics, as I feel they were somewhat under explored in early work. However, the readability of the manuscript leaves a lot to be desired, and I suggest the authors make an effort to make the manuscript a bit more accessible to people aren't experts in f-statistics.

We tried to follow this recommendation to make the manuscript more accessible to non-expert audience: added paragraphs introducing *f*-statistics and admixture graphs, updated all the sections, harmonized the notations of ascertainment schemes, and explained all the abbreviations in the legends and in the text.

I have a couple of other minor comments.

1) The authors use a criterion on the size of the "worst residuals" (WR) from a graph to determine whether to reject the graph or not. Are the properties of this approach known? Does the test have the same size for all topologies with the same number of leaves, or does the internal structure of a topology impact the size of the test? I suppose my concern is that there may be different power and size for different topologies, making it somewhat difficult to interpret the exhaustive enumeration over topologies that the authors performed.

We relied on worst residuals of admixture graphs (Z-score of an *f*-statistic which is fitted the worst by the model, see lines 290-302 in the updated manuscript for a definition) since this is a metric very often used in practice for assessing fits of admixture graph models to data (see our study focused on admixture graphs, Maier et al. 2023 “On the limits of fitting complex models of population history to *f*-statistics” *eLife*). By convention, graphs with WR above 3 standard errors are considered fitting the data poorly (see, e.g., Lazaridis et al. 2014, Lipson and Reich 2017, Flegontov et al. 2019, Wang et al. 2021, Changmai et al. 2022). The question that you raised is valid, and we are not aware of its detailed exploration on simulated data. However, since our study is oriented by design more towards practice than theory, we would prefer to keep this difficult issue out of the scope of the paper, and just to use a conventional metric while simultaneously flagging that this metric has limitations (see lines 300-302).

2) Fig 1 is very complicated. I do think it actually conveys a lot of useful information, but it's pretty hard to parse. I think one easy fix is to try to move the 1240K WR example panel out of the middle. It took me a long time to realize that that panel wasn't "special" and was just like the other panels outside the middle.

We have removed the panel out of the middle and made further changes to simplify the figure (see Figure 2 in the revised manuscript). Following suggestions by two reviewers,

we have placed the scatterplots as numbered panels below the main plot and removed the admixture graph examples as not particularly informative.

3) I wonder if Fig 2e can be simplified by presenting it with a log scale on the y axis? That way there may not be a need to have 3 different panels with different y axes, which makes it very weird to look at.

We have simplified this figure panel by presenting it with a square root scale and dropping the bins corresponding to sites with fixed derived and fixed ancestral alleles in the root population. Now we show one panel instead of 3, for derived allele counts from 1 to 19. Complete results are presented in Suppl. Fig. 8.

4) Figure 3 seems to just be missing.

Thanks for spotting this! It was just a figure numbering issue. We have corrected the figure citation, and now it points to Suppl. Fig. 10.

I prefer to sign my reviews. My name is Joshua Schraiber

Reviewer #2: The authors present a major study comparing how different ascertainment schemes affect the results given by f4 statistics and reconstructed admixture graphs when including genomic data of individuals from Africa. The manuscript shows the impact of using different ascertainment schemes on whole-genome sequencing data from SGDP, ancient humans and archaic hominins. These analysis show that some ascertainment schemes, such as using polymorphic variants based on data from three archaic hominins, give poorer results compared to other ascertainment schemes that include using particular panels or using variants that are common on many populations in Africa. Then, the authors perform simulations under a realistic demographic scenario to show that there are few biases in the inferred admixture graph when using a scheme similar to the Human Origins SNP panel or using a scheme that includes common variants from Africa.

This is a manuscript that describes an important problem to investigate the past history of Africa. The paper describes very well thought experiments and the authors have made a great effort to understand the impact of ascertainment schemes on the inference of admixture graphs.

We thank the reviewer for this favorable assessment of our work!

There are a couple of things that I think would be helpful for readers of this paper: 1) Describe with a little bit more detail the main metric used to analyze how the ascertainment

schemes bias demographic reconstructions. Explaining with detail what are "the worst f4-statistic residuals (WR) for graphs" would be useful for readers to assess why particular ascertainment biases could be problematic for studies of past population history.

We relied on worst f_4-statistic residuals of admixture graphs or WR since this is a metric very often used in practice for assessing fits of admixture graph models to data (see our study focused on admixture graphs, Maier et al. 2023 "On the limits of fitting complex models of population history to *f*-statistics" *eLife*). By convention, graphs with WR above 3 standard errors are considered to fit the data poorly (see, e.g., Lazaridis et al. 2014, Lipson and Reich 2017, Flegontov et al. 2019, Wang et al. 2021, Changmai et al. 2022). To aid the reader, we have added definitions of the WR and LL metrics to the legend for Figure 1 (formerly Suppl. Fig. 1) and in the opening paragraphs of the Results section, lines 290-308:

"WR, also referred to as "admixture graph Z-score", is one of two key metrics of admixture graph fit used in this study: it is the Z-score measuring deviation between observed and expected values of an f_4-statistic that is predicted most poorly by the admixture graph being tested (Lipson 2020, Maier et al. 2023). WR is measured in standard error (SE) intervals, and, by convention, admixture graphs with WR below 3 SE are considered to fit the data well (see, e.g., Lazaridis et al. 2014, Lipson and Reich 2017, Flegontov et al. 2019, Wang et al. 2021, Changmai et al. 2022). Thus, WR is typically used in the literature to assess absolute fit of admixture graph models to data (e.g., Bergström et al. 2020b, Lazaridis et al. 2014, Lipson and Reich 2017, Flegontov et al. 2019, Wang et al. 2021, Changmai et al. 2022), and it is used for model ranking in some cases (strictly speaking, WR it is just an approximation of absolute model fit, which is hard to calculate since many *f*-statistics for a given population set are correlated, Maier et al. 2023). Log-likelihood score (LL score or simply LL) is another metric that is dependent on deviations of all *f*-statistics (for populations included in the model) from their predicted values and on their covariance, and thus more accurately reflects model fit to data (Lipson and Reich 2017, Maier et al. 2023). However, unlike WR measured in SE units, LL is not easily comparable across admixture graph complexity classes, population sets, and SNP sets (but comparable across topologies of the same complexity on the same set of SNPs and populations), and thus WR is used as the primary admixture graph fit metric in this study."

2) I think it would be very helpful to use consistent abbreviations and notations in the Figures and manuscript that identify all the ascertainment schemes used in the analysis performed in the real data. This would make it easier to follow the results presented by the authors. I would also suggest using a notation and abbreviation for the simulation section that is consistent with the section on the analysis of real data.

In some figures (e.g., Figs. 2a, 5) classes of ascertainment schemes are labelled (e.g., all Human Origins panels ascertained on single individuals, or all ascertainments based on a minor allele frequency threshold), while in other cases results for individual ascertainment schemes are presented (Figs. 1, 2b-h, 6, Table 1). The notations are necessarily a bit different in these cases.

To harmonize the notation for ascertainment schemes, we have relabeled the plots in all relevant main and supplementary figures and modified the tables. Here is a list of changes in the figures and tables: AT GC => AT/GC; other panels => 1000K & 2200K; 1240K comp. => 1240K components; arch. asc. => archaic asc.; arch.transv. => archaic asc., transv.; MAF 5% => MAF; MAF AFR => AFR MAF, and other changes. We have also relabeled all the plots in Fig. 5 and Suppl. Fig. 11. The grouping and coloring schemes for ascertainments were also changed in the latter figures to make them identical to those in Fig. 1a and Suppl. Figs. 2 and 3. Now the same colors and groupings are used for visualizing effects of ascertainment on both admixture graphs fits and f_4-statistics.

The complete list of annotation schemes applied to real data is presented at the beginning of Results, and in the legends for Fig. 2. In the revised manuscript, we have added abbreviated names of ascertainment schemes to that list and harmonized their notation throughout the main and supplementary text, figure and table legends. We have also modified the notation of ascertainment schemes on simulated data to harmonize it with that on real data (see Figs. 3 and 4, Suppl. Figs. 6a-f, 8-10, 16c, and 17c, Suppl. Table 6). However, some ascertainment schemes on simulated data do not have exact counterparts on real data.

Additionally I have a few comments on the manuscript.

Line 115.- "However, evidence is accumulating that supports archaic admixture in Africans (Chen et al. 2020, Hubisz et al. 2020)" This paper contests that claim (https://www.biorxiv.org/content/10.1101/2022.03.23.485528v3.abstract). I think it would be good to mention it since it provides good evidence contesting that sentence.

Thanks for this suggestion! We have rephrased our sentences and cited this important paper, see lines 178-187:

"Existing recommendations for a bias-free SNP enrichment panel also rely on the assumption that archaic humans are nearly perfect outgroups with respect to all AMH, and the expectation that the low-level archaic admixture in non-Africans (Green et al. 2010, Reich et al. 2010) subsequently carried back into Africa to a small extent (Prüfer et al. 2017, Chen et al. 2020) does not contribute substantial bias. But evidence is accumulating in favor of long-lasting population structure in Africa or introgression from an unsampled deeply-diverging archaic group to a common ancestor of AMH (Hammer et al. 2011, Ragsdale and Gravel 2019, Speidel et al. 2019, Durvasula and Sankararaman 2020, Hubisz et al. 2020, Ragsdale et al. 2023), and it remains unclear how this complex demographic history affects the performance of archaic ascertainment."

Lines 164-167.- Can the authors briefly explain what the motivation is behind using this ascertainment scheme?

We tested the performance of several SNP panels comprising the Human Origins SNP array for the following reasons. They are rarely used in practice as standalone panels for analyses, but they were designed as "clean" forms of ascertainment in the paper where they were introduced (Patterson et al. 2012), and we wanted to check if this holds. See an additional explanation we have added to the Discussion, lines 749-755:

"We tested several of the panels comprising the Affymetrix Human Origins SNP array (the largest of them), each ascertained as sites heterozygous in a high-coverage human genome from a selected population, since they were proposed to be "clean" forms of ascertainment in the publication where they were introduced (Patterson et al. 2012). HO panels are rarely used in practice individually because of their small size, which is especially problematic for ancient individuals with high rates of missing data, and our results confirm that this practice is justified."

Legend on Supp Fig. 1.- "Suppl. Fig. 1. Scatterplots illustrating the effects of the 1240K ascertainment on LL and WR for exhaustive collections of simple admixture graphs." It would be useful for the reader to define LL and WR in the text before pointing to this figure. It would be good to give a clear explanation of LL on the main text.

To aid the reader, in the revised manuscript we have added definitions of the WR and LL admixture graph fit metrics to the legend for Figure 1 (formerly Suppl. Fig. 1) and in the opening paragraphs of the Results section, lines 290-308:

"WR, also referred to as "admixture graph Z-score", is one of two key metrics of admixture graph fit used in this study: it is the Z-score measuring deviation between observed and expected values of an f_4-statistic that is predicted most poorly by the admixture graph being tested (Lipson 2020, Maier et al. 2023). WR is measured in standard error (SE) intervals, and, by convention, admixture graphs with WR below 3 SE are considered to fit the data well (see, e.g., Lazaridis et al. 2014, Lipson and Reich 2017, Flegontov et al. 2019, Wang et al. 2021, Changmai et al. 2022). Thus, WR is typically used in the literature to assess absolute fit of admixture graph models to data (e.g., Bergström et al. 2020b, Lazaridis et al. 2014, Lipson and Reich 2017, Flegontov et al. 2019, Wang et al. 2021, Changmai et al. 2022), and it is used for model ranking in some cases (strictly speaking, WR it is just an approximation of absolute model fit, which is hard to calculate since many *f*-statistics for a given population set are correlated, Maier et al. 2023). Log-likelihood score (LL score or simply LL) is another metric that is dependent on deviations of all *f*-statistics (for populations included in the model) from their predicted values and on their covariance, and thus more accurately reflects model fit to data (Lipson and Reich 2017, Maier et al. 2023). However, unlike WR measured in SE units, LL is not easily comparable across admixture graph complexity classes, population sets, and SNP sets (but comparable across topologies of the same complexity on the same set of SNPs and populations), and thus WR is used as the primary admixture graph fit metric in this study.

Line 190-193.- "the worst f4-statistic residuals (WR) for graphs including one archaic human, three African groups, and one African group with ca. 60% of non-African ancestry (Fan et al. 2019) are poorly correlated on all sites and 1240K sites (R = 0.31-0.35)." Can you

devote more space to explain the metric WR? This metric is key on the first set of analysis and the manuscript would be easier to follow if it is defined explicitly.

We relied on worst f_4-statistic residuals of admixture graphs or WR since this is a metric very often used in practice for assessing fits of admixture graph models to data (see our study focused on admixture graphs, Maier et al. 2023 "On the limits of fitting complex models of population history to *f*-statistics" *eLife*). By convention, graphs with WR above 3 standard errors are considered to fit the data poorly (see, e.g., Lazaridis et al. 2014, Lipson and Reich 2017, Flegontov et al. 2019, Wang et al. 2021, Changmai et al. 2022). To aid the reader, in the revised manuscript we have added a definition of the WR metric to the legend for Figure 1 (formerly Suppl. Fig. 1) and in the opening paragraphs of the Results section, see lines 290-308.

"Suppl. Fig. 2. Two alternative approaches for visualizing the effect of ascertainment bias on admixture graph fits illustrated using one population combination, "Denisovan, Khomani San, Mbuti, Dinka, Mursi"." What are the two alternative approaches? I think it would be good to mention this very explicitly.

We have clarified this in the legend for Suppl. Fig. 2 (now Suppl. Fig. 1):

"Two alternative approaches for measuring the effect of ascertainment bias on admixture graph fits are illustrated using one population combination, "Denisovan, Khomani San, Mbuti, Dinka, Mursi": 1) residual standard deviation (residual SE) of linear trends and 2) squared Pearson correlation coefficient (R^2) for two admixture graph fit metrics (worst f_4-statistic residuals, WR, or log-likelihood scores, LL) calculated on unascertained vs. ascertained data."

Figure 1.- I find the admixture graphs added on the figure not particularly helpful. I do not think they are helpful to understand the main point of this Figure.

We agree, and we have removed these graphs for clarity (see Figure 2 in the revised manuscript).

Figure 3 is missing from the manuscript.

Thanks for spotting this! It was just a figure numbering issue. We have corrected the figure citation, and now it points to Suppl. Fig. 10.

Reviewer #3: The paper presents an important and comprehensive study of bias caused by SNP ascertainment for commonly used statistics and inference methods in human (ancient) population genetics, specifically methods to measure and interpret human population structure in light of human history.

I think the study is extremely thorough, and the content certainly important enough for the readership of this journal, in my view.

The text is well written and (mostly) guides the reader sufficiently through the complex experiments and results. I have not much to criticise about the actual experiments and content of the study.

We thank the reviewer for this favorable assessment of our work!

My main criticism concern the Figures, some of which are too overloaded and not sufficiently clear. What follows are suggestions on how to improve them.

Figure 1:
I think the chaotic layout does not work and obfuscates the main figure. My suggestion would be to make the main figure (R-squared of WR against various ascertainment panels) panel a), and then place selected WR scatter plots (perhaps three representative ones ?) orderly below as panels b-d. Panels e-g could then be the three trees that are shown. I am not sure whether they are needed though. In any case, _all_ sub-panels must respect a minimum font size of 5 for any text in them, when rescaled to a full width figure. To link the WR-scatter plots with the points in panel a, the authors used purple lines. I suggest to remove them for clarity, and instead mark the selected points with little numbers or symbols which could then be used in the scatter plots and trees to link them with panel a.

We have modified the figure as suggested (see Figure 2 in the revised manuscript). We decided to remove the examples of admixture graphs since they are less important than the WR scatterplots. We have also modified Suppl. Fig. 1 in a similar way.

Caption: The terms WR and LL need to be introduced right in the caption. I think it is I not sufficient to just introduce these key terms in the main text. It is already challenging enough to go through all the technical details, and it should not be made harder by having to look up these terms first when looking at the figure for the first time. I think introducing them once in the Caption of Figure 1 may be sufficient for the subsequent figures as well.

In the revised manuscript we introduce these terms in the opening paragraphs of the Results section (lines 290-308) and in the legend for Figure 1 (formerly Suppl. Fig. 1): "WR, also known as admixture graph Z-score, is the residual of an f_4-statistic that is fitted the worst by the admixture graph model. Log-likelihood score of an admixture graph model (LL, Maier et al. 2023) reflects deviation of all relevant *f*-statistics from their values predicted under the model and their covariance."

We have also modified all the main and suppl. figure/table legends spelling out the WR and LL abbreviations everywhere.

As an additional comment on this subject: I like Supplementary Figure 1, which tells a clear story and is quick to understand. Perhaps one should consider moving it into the main text.

We agree, and we have moved this figure to the main text (see Figure 1).

Figure 2:

My general comment on Figure 2 is to separate it into multiple figures. It's just too much, and they describe separate enough things to justify splitting them apart. My suggestion is to put 2a+b into one figure, and c-f into another one.

We have split the figure, as suggested, into Figs. 3 and 4.

Specific comments on subpanels of Figure 2:
a) The dozen or so dotted lines are a distraction, and I would suggest considering simply a linear, or partially linear, or logarithmic time scale on the y axis. The actual year numbers (which are given in unnecessary 4-5 significant digits) can be better included into Supplementary Table 13, which already lists the population sizes.

We have now used a partially linear scale on the y-axis (see Fig. 3a in the revised manuscript). The values on these axes are in generations, not in years. We have removed the dotted lines connecting demographic events and exact dates on the y-axis and also removed effective population sizes from the panel. For a full list of parameter values see Suppl. Table 13.

b) Too small. Again, check the font sizes, they should not be smaller than 5-6pt when scaled on full-width. Perhaps move the legend above the plots, next to panel a) or so, to have more width for the 8 charts. One could also consider ditching too of the trees or so, for example the ones without Neanderthal admixture, which could then simply be described as a special case of the trees with admixture.

We have moved the legend and increased font sizes throughout this panel (the smallest size is now 6pt), see Fig. 3b. We preferred to keep all the graphs in this panel, but we added more understandable titles for the four sub-panels, explaining the differences between the four admixture graphs.

c) I could not follow panel c at all. Neither in the caption nor in the main text it is sufficiently explained what the labels on the left mean. For example, "HO-1 non-OG groups (all asc. Inds)" is cryptic and not explained. When the figure is first introduced in the text, it says in lines 366-368 "As illustrated by distributions of true admixture graph WRs in Fig. 2c, ‘blindly’ ascertaining on individuals or sets of groups randomly sampled across the graph almost guarantees rejecting the true historical model by a wide margin". If that is referring to

the two red box plots labeled "HO-1 OG (one ind.)" and "HO-1 non-OG groups (one ind.)", that is pure guess-work on my end, because it is not explained. The caption lists various ascertainment schemes for panel c) but with no obvious link to the abbreviations used in the labels. Please expand this, perhaps it gets easier when splitting up that figure as I suggested.

Different types of simulated populations were used for Human Origins-like ascertainment (that is selecting sites heterozygous in a randomly selected individual from one population): the root of the simulation, the outgroup with a large or small effective size, or the other (non-outgroup) populations. In addition to that, either the individual used for ascertainment was a sole representative of its group in the fitted graph, or the whole group was included in the fitted graph, or the ascertainment group was not included in the fitted model at all. These details are now reflected in the plot labels on the right and on the y-axis. We have updated the legend for this figure panel extensively (now Fig. 4a) and have defined all the abbreviations in this legend.

d) is fine.

Now it is Fig. 4b.

e) I found a bit unclear why it is needed and what it tells me. What I see is that the derived allele frequency spectra for the "true root" are i) substantially different from any spectrum in the leaves, and ii) that the leave spectra are a lot noiser than the root spectrum. But there is very little difference between the differently grouped leave spectra, and in my view the text doesn't really help to guide the reader through this figure. I think if the authors would like to keep this panel as a main figure, the text needs to describe it properly and tell the reader what to look at and what how to interpret it.

We agree that this analysis is not as informative as suggested in the first version of our manuscript, that's why we have rephrased the main text (lines 513-517):

"Another way of looking at this phenomenon is through derived allele frequency (DAF) spectra. Ascertainment schemes resulting in relatively unbiased fits of true models (WR <4 SE, Fig. 4c) are most often based on populations where the DAF spectrum of sites that were polymorphic at the root is preserved relatively well (see a full version of this plot in Suppl. Fig. 8)."

See also the modified legend for Fig. 4c.

f) is fine.

This panel has been removed since the same boxplots are now shown in Fig. 4a.

Figure 3:

Was completely missing from the version of the article that was made available to me. I could not review it.

Thanks for spotting this! It was just a figure numbering issue. We have corrected the figure citation, and now it points to Suppl. Fig. 10.

Figure 4:
The titles of the three panels use the naming scheme introduced in the text, for example AFR2, ARCH 1, ME1. In the caption, this is translated to more concrete F4-statistic-classes. I would suggest to use the F4-form from the caption also as title in the plots to make things easier for the reader.

Figure 5:
Same point as with Figure 4: I suggest to avoid things like F4(AFR3,ARCH1) and instead write F4(AfrX, AfrY; AfrZ, Archaic) or so in the title.

Following these two comments, we have modified the notation of *f4*-statistics throughout the text, main figures, and all suppl. figures and tables. Now the notation follows the following format: f_4(African$_x$, African$_y$; African$_z$, archaic). The only exception is Suppl. Fig. 12 where full notation of f_4-statistic classes on the y-axis would compromise readability, in our view. There we preserved the original notation of the style f_4(African 3, archaic 1), however we have provided additional explanations in the legend and in the y-axis labels.

More minor comments:

Supp Fig 1: Again, please explain what WR and LL is. Those abbreviations are explained only in the main text, and in fact after the first mention of Supp Fig 1. Also, the legend of Supp Fig 1 mentions "LL" while I think that is not actually shown, is it?

Indeed, no LL was shown, it was our mistake. In the revised manuscript, we have explained the LL and WR abbreviations in the opening paragraphs of the Results section (lines 290-308) and in the figure legend (now Figure 1): "WR, also known as admixture graph Z-score, is the residual of an f_4-statistic that is fitted the worst by the admixture graph model. Log-likelihood score of an admixture graph model (LL, Maier et al. 2023) reflects deviation of all relevant *f*-statistics from their values predicted under the model and their covariance."

We have also modified all the main and suppl. figure/table legends spelling out the WR and LL abbreviations everywhere.

Table 1: Since this is a main table: Why showing so many different population quintuplets? Aren't three or four representative quintuplets sufficient? And do we need three digits in percent numbers?

To simplify the table, we have collapsed five columns containing only zeros (or just one non-zero value) into one. We believe that it is worth showing not just three or four, but all the remaining population quintuplets to support the summary statistics in the rightmost columns; and those statistics are used to rank ascertainment schemes in the main text. We have switched to two-digit precision in Table 1, as suggested, and also in Suppl. Tables 3 to 6.

Fig 2a: In the caption, it would help if we could know what 'd', 'n', 'a' and so on stand for.

We have explained the abbreviations in this panel (now Fig. 3a).

L 665 "one individual sampled at the end of the simulation" What does "end of the simulation" mean here?

It means at present in the simulation time. We have clarified that at all places in the main text.

Suppl Fig 17b. Too cluttered in my view. Consider moving at least the population sizes into a table?

These are not population sizes, but dates of admixture and divergence events. To simplify Suppl. Figs 17b and 18b (now 16b and 17b), we have decided to show in the first sub-panel (Model1) parameters that are the same across all five simulations and omit them from the other sub-panels (Models2-5). For a full list of parameters see Suppl. Table 13.
